# Supplementary material for: TXNIP mediates LAT1/SLC7A5 endocytosis to limit amino acid uptake in cells entering quiescence
Source: EMBO J. 2025 Oct 20;44(23):7119–53. doi: 10.1038/s44318-025-00608-9 (PMC12669767; doi:10.1038/s44318-025-00608-9)
Supplement: Supplementary file 1 — Appendix [file 44318_2025_608_MOESM1_ESM.pdf]

Appendix for
'TXNIP mediates LAT1/SLC7A5 endocytosis to limit amino acid uptake in cells entering quiescence'.

Table of contents:

Appendix Figure S1……………………1
Appendix Figure S2……………………3
Appendix Figure S3……………………5
Appendix Figure S4……………………7
Appendix Figure S5……………………9
Appendix Patient Information………....11
Appendix Table S1…………………….12
Appendix Table S2…………………….13
Appendix Table S3…………………….17
Appendix Table S4…………………….19
Appendix Table S5…………………….20
Appendix References............………...20

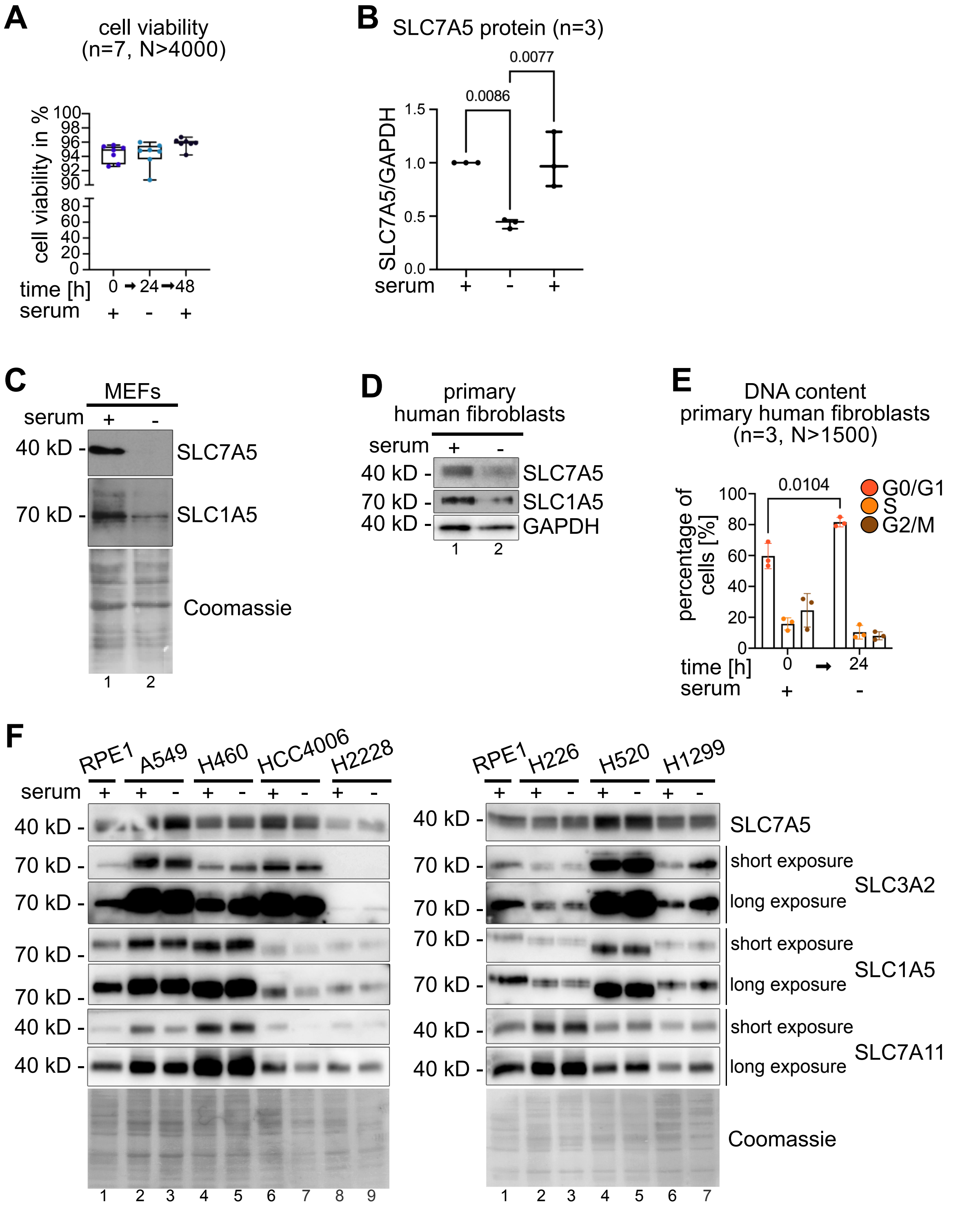

Appendix Figure S1

Appendix Figure S1.

(A) Cell viability determined with CASY counter, before and after serum starvation and after re-addition of serum (n=7, N>4000 cells). **(B)** WB quantification of SLC7A5 under serum-supplemented (+serum) or serum-starved (-serum) conditions, normalized to GAPDH (n=3, paired t-test). **(C)** Immortalized MEFs were grown in growth medium with serum (+ serum) or serum starved for 24 h. Total cell lysates were analyzed by SDS-PAGE and WB with the indicated antibodies. **(D, E)** Primary human fibroblasts were grown in growth medium (+ serum) or serum starved (-serum) for 24 h. **(D)** Total cell lysates were analyzed by SDS-PAGE and WB with the indicated antibodies. **(E)** Cells were harvested, permeabilized and fixed. The DNA content was analyzed by PI staining and FACS (n=3, N>1500 cells, two-way ANOVA, Tukey's multiple comparisons test). **(F)** Total cell lysates of the indicated cell lines were analyzed by SDS-PAGE and WB with the indicated antibodies.

A

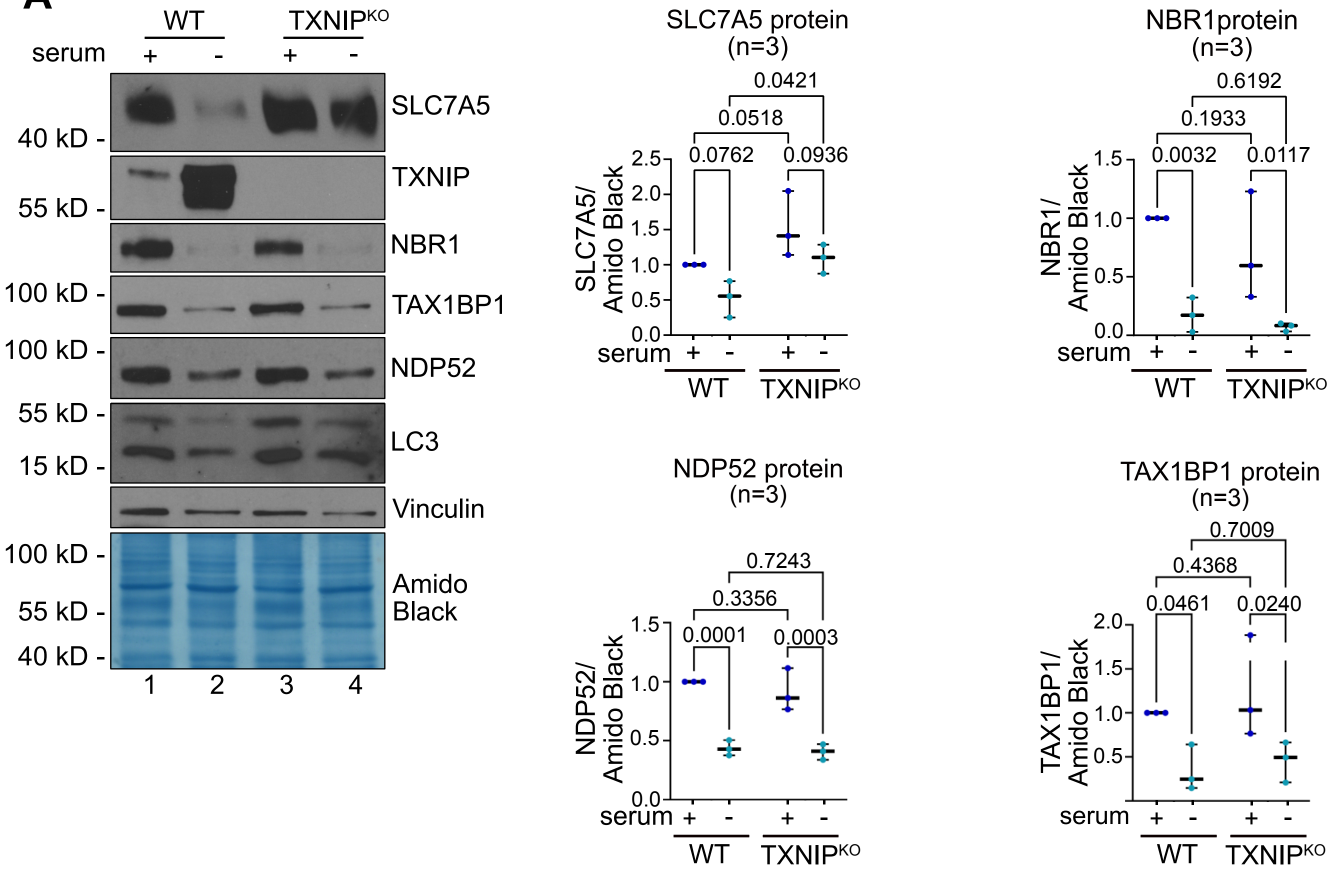

B

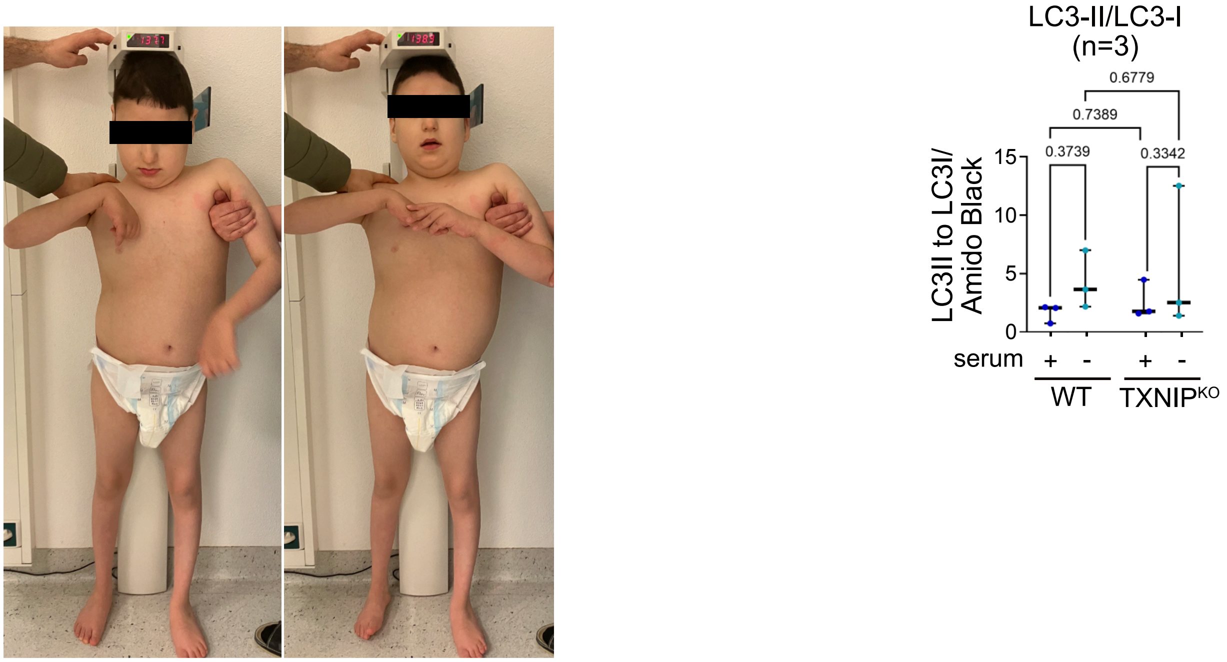

Appendix Figure S2

Appendix Figure S2.

(A) WT and TXNIP^{KO} cells were grown in growth medium (+ serum), or serum starved for 24 h (- serum). Total cell lysates were analyzed by SDS-PAGE and WB with the indicated antibodies. WB quantification of SCL7A5, NBR1, TAX1BP1, NDP62 and LC3 normalized to amido black (n=3, two-way ANOVA, Tukey multiple comparison test). **(B)** Photographs of the patient carrying the TXNIP variant c.642_643insT (p.Ile215Tyrfs*59) at 11 years of age. Marked muscular hypotonia is evident, and the patient is unable to stand unassisted.

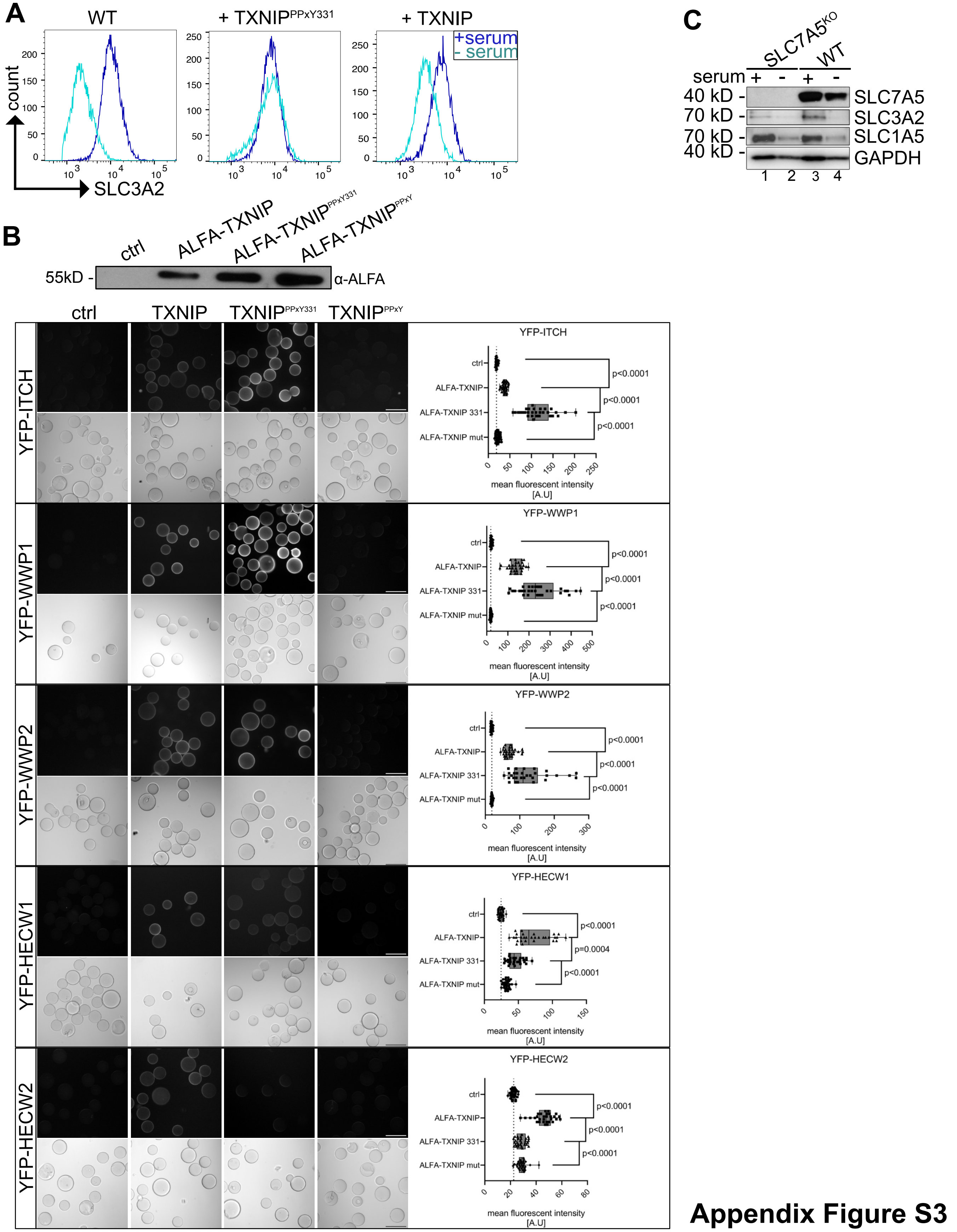

Appendix Figure S3

Appendix Figure S3.

(A) The cell surface was stained with anti-SLC3A2 and anti-mouse Alexa-488. FACS was used to detect SLC3A2 surface staining. A representative histogram is shown. **(B)** SDS-PAGE and WB analysis of immobilized ALFA-TXNIP (and the indicated PPxY mutants) used in the assay. Representative bright-field and fluorescence microscopy images of YFP-ITCH, YFP-WWP1, YFP-WWP2, YFP-HECW1 and YFP-HECW2 bound to ALFA-TXNIP (and the indicated PPxY mutants) immobilized on beads. Quantifications of the YFP-HECT type fluorescence signal (n=40 beads). Data is represented as single data-points including the mean and the 25-75 percentile indicated by the box. **(C)** Total cell lysates of RPE1 SLC7A5^{KO} and wild-type cells that were grown in growth medium (+ serum) or were serum starved for 24 h (- serum) were analyzed by SDS-PAGE and WB with the indicated antibodies

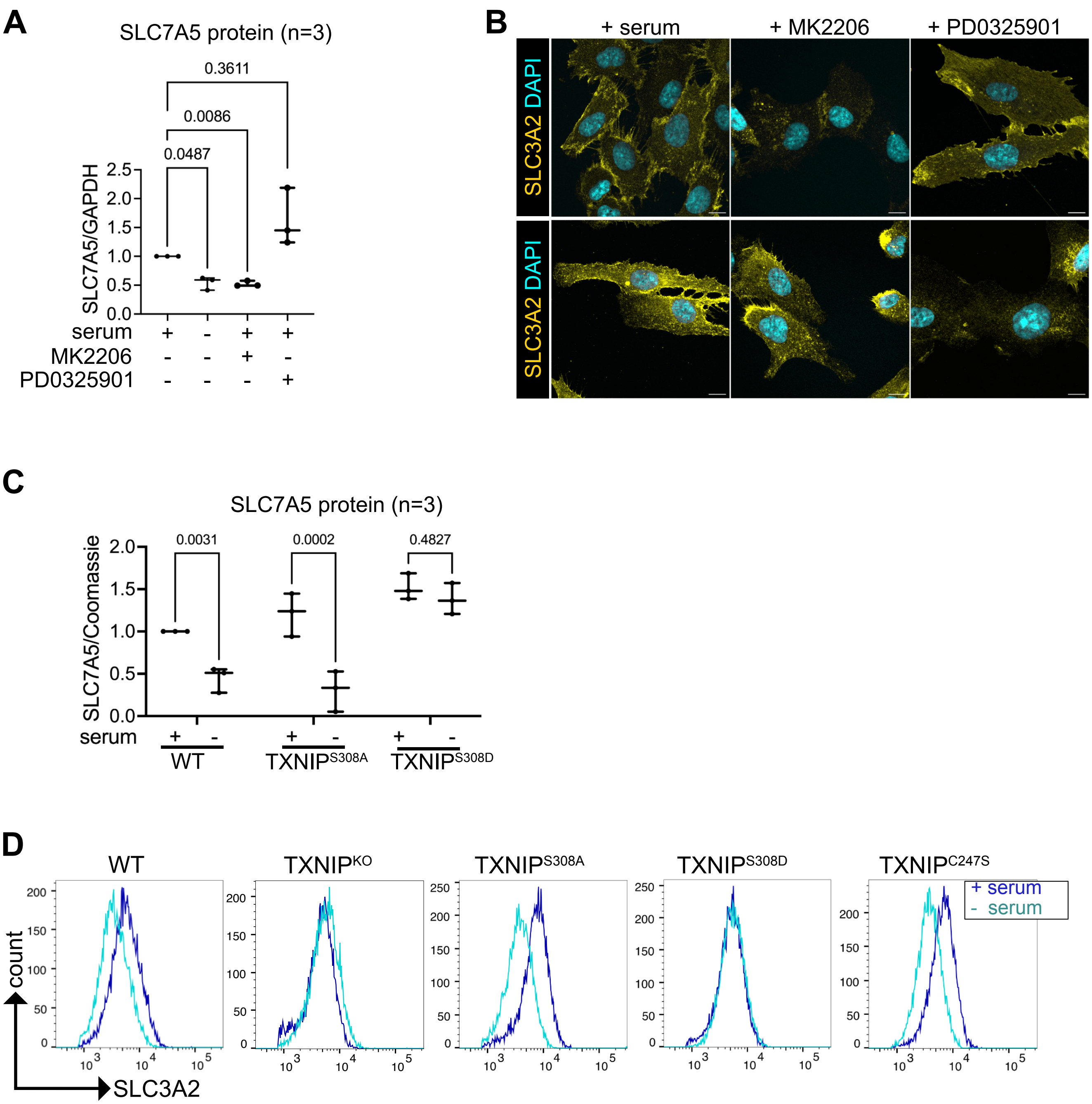

Appendix Figure S4

Appendix Figure S4.

(A) WB quantification of SLC7A5 protein levels, normalized to GAPDH (n=4, two-way ANOVA, Sidak's multiple comparisons test) of cells before or after serum starvation or incubation with 1 µM MK2206 or 5 µM PD0325901. **(B)** Indirect IF of PFA fixed cells cultured upon standard cell culture conditions or incubation with 1 µM MK2206 or 5 µM PD0325901 (PD) stained for SLC3A2 (green, upper row) or SLC1A5 (yellow, lower row), and DAPI (cyan). Cells were analysed by confocal microscopy. The images show a single plane of a Z-stack. Scale bar = 10 µm **(C)** WB quantification of SLC7A5 protein levels, normalized to Coomassie of RPE1 WT cells and TXNIP KO cells, reconstituted with $TXNIP^{S308A}$ or $TXNIP^{S308D}$ before or after 24h of serum starvation. (n=3, two-way ANOVA, Sidak's multiple comparisons test) **(D)** representative histograms of SLC3A2 cells surface FACS of the indicated cells.

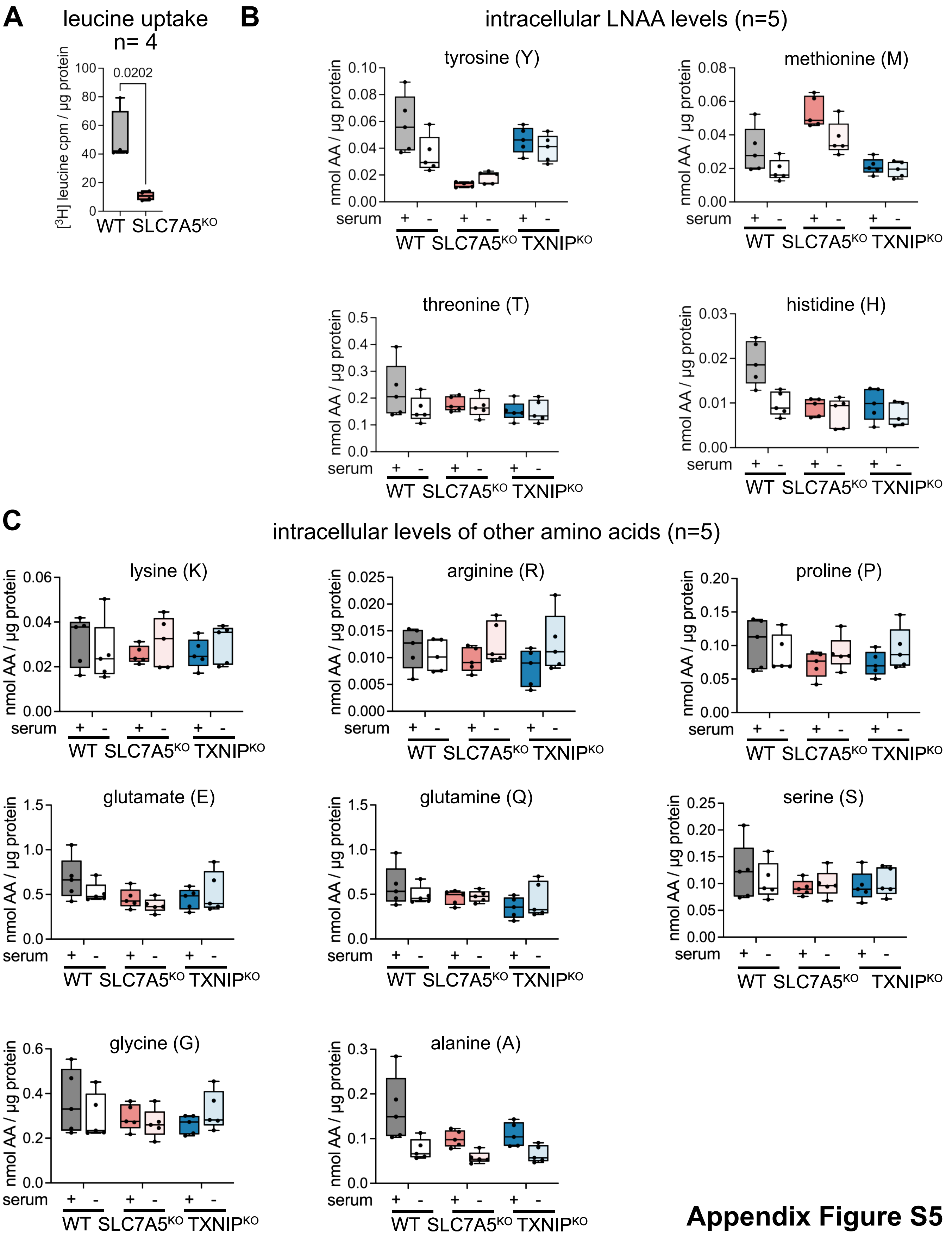

Appendix Figure S5

Appendix Figure S5.

(a) WT and SLC7A5 KO cells were incubated with [3 H]-leucine for 15 min, washed and lysed. Cell lysates were analysed by scintillation counting. cpm values were normalized to total protein content (n=4, paired t-test). **(B, C)** WT, SLC7A5 KO and TXNIP KO cells were grown in growth medium (+ serum) or serum starved for 24 h (- serum). Mass spectrometry analysis of free AAs, normalized to total protein content (nmol AA / μ g protein, n=5).

Appendix Patient information:

The patient is a boy, born in 2014 as the first child of healthy, consanguineous parents of Turkish origin. During pregnancy, the mother was diagnosed with polyhydramnios. At 38 + 6 weeks of gestation, the baby was in a breech position, leading to a caesarean section. At birth, he weighed 3880 g (P90), measured 55 cm in length, and had a head circumference of 38 cm.

On the seventh day of life, he exhibited floppiness, recurrent hypoglycaemia, and lactic acidosis, prompting his transfer from the birth hospital to a tertiary care centre. During the first three days there, his lowest recorded blood glucose level was 30 mg/dl, lactate levels were approximately 6.5 mmol/l, and pH was 7.11. Subsequently, he developed hypertriglyceridemia, with triglyceride levels reaching 364 mg/dl. Initially stable, he began experiencing elevated pCO_2 levels (up to 70 mmHg due to bradypnea) and metabolic acidosis on day 10. A glucose infusion (10 mg/kg/min) stabilized his glucose and lactate concentrations, though lactate remained elevated at around 3-4 mmol/l. Regardless, his muscular hypotonia persisted. On day 12, a skin punch biopsy for a fibroblast culture was performed.

By day 20, glucose and lactate levels had stabilized with regular feeding, allowing his transfer back to a peripheral hospital. During infancy, his blood glucose concentrations were within standard range (Appendix Table 1), but the boy experienced recurrent hypoglycaemia in response to metabolic stress, e.g., infections. He exhibited psychomotor developmental delays and, from 18 months of age, experienced increasing epileptic seizures (up to 3-4 per month), which were managed with levetiracetam, topiramate, and lamotrigine. Currently, he remains metabolically stable but presents with significant developmental delay, muscular hypotonia, and autistic features (see Appendix Fig. S2B).

Appendix Table S1. AA, glucose and lactate levels in blood plasma of the TXNIP deficient patient over the indicated years.

AA [µmol/l]	06/2014	07/2014	Reference values	02/2016	03/2018	11/2021	09/2022	06/2025	Reference values
leucine (L)	60.51	175.52	46 - 109	346.58	226.89	212.26	141.50	133.8	45 - 155
isoleucine (I)	13.38	91.56	26 - 53	173.06	132.25	129.59	82.64	70.19	26 - 94
valine (V)	99.97	148.00	57 - 262	529.46	376.67	389.20	229.86	246.43	57 - 270
phenylalanine (F)	46.45	41.50	23 - 110	84.41	54.64	53.02	56.75	43.40	23 - 70
tyrosine (Y)	38.54	52.02	11 - 112	110.43	70.10	51.76	50.47	40.56	11 - 120
tryptophan (W)	25.21	51.44	n.a	76.35	45.51	40.21	41.48	44.58	n.a
proline (P)	191.78	197.21	52 - 277	264.22	228.78	214.37	275.47	230.68	51 - 185
alanine (A)	517.23	456.10	99 - 380	354.58	350.13	214.18	456.10	574.22	99 - 350
methionine (M)	12.14	24.43	3 - 41	36.19	18.77	10.83	12.30	10.04	3 - 29
Fischer's ratio (FR)	2.05	4.46	2.10 - 4	5.38	5.90	6.98	4.23	5.36	2.10 - 4
**glucose [mg/dl]**	34	71	> 50	88 *	78	86	103	93	> 50
**lactate [mg/dl]**	68.30	46	5 - 22	24.33 *	50.5	26	56	50	5 - 22

* The glucose and lactate values were collected on a different day than the AA levels.

Appendix Table S2. Summary of antibodies, reagents and software used in this study.

**Reagents/resource**	**Reference or source**	**Identifier or catalog number**	**Additional information**
**Antibodies**			
SLC1A5/ASCT2	Merck	#abn73, RRID:AB_10807715	WB (1:1000)
SLC1A5/ASCT2 (V501)	Cell Signaling	#5345, RRID:AB_10621427	WB (1:1000)
SLC1A5/ASCT2	Sigma-Aldrich	#HPA035240, RRID:AB_10604092	IF (1:50)
SLC7A5/LAT1 (polyclonal)	Cell Signaling	#5347S, RRID:AB_10695104	WB (1:2000)
SLC7A5/LAT1 (E9O4D) (monoclonal)	Cell Signaling	#32683	WB (1:2000)
SLC7A5/LAT1 (D10)	Santa Cruz	#sc-374232, RRID:AB_10988206	WB (1:100)
SLC3A2/4F2hc (D6O3P)	Cell Signaling	#13180S, RRID:AB_2687475	WB (1:1000)
SLC3A2/4F2hc	BD Biosciences	#556074, RRID:AB_396341	IF, FACS (1:100)
SLC2A1/Glut1	Merck	#07-1401, RRID:AB_1587074	WB (1:2000), IF (1:200)
SLC38A2/SNAT2	MBL International	#BMP081, RRID:AB_10597880	WB (1:100)
SLC7A11/xCT (D2M7A)	Cell Signaling	#12691, RRID:AB_2687474	WB (1:1000)
TXNIP (D5F3E)	Cell Signaling	#14715S, RRID:AB_2714178	WB (1:2000)
p-S6K^{T389} (108D2)	Cell Signaling	#9234S, RRID:AB_2269803	WB (1:1000)
S6K	Cell Signaling	#9202S, RRID:AB_331676	WB (1:1000)
p-S6$^{S240/244}$	Cell Signaling	#2215S, RRID:AB_331682	WB (1:2000)
S6 (54D2)	Cell Signaling	#2317S, RRID:AB_2238583	WB (1:1000)
p-EIF2α	Cell Signaling	#3398 RRID:AB_2096481	WB (1:1000)
EIF2α	Cell Signaling	#5324 RRID:AB_10692650	WB (1:1000)
p-ERK1/2$^{T202/Y204}$ (D13.14.4E)	Cell Signaling	#4370S, RRID:AB_2315112	WB (1:1000)
ERK1/2	Cell Signaling	#9102S, RRID:AB_330744	WB (1:1000)
p27^{Kip1} (D69C12)	Cell Signaling	#3686S, RRID:AB_2077850	WB (1:1000)
p-AKT^{S473} (D9E)	Cell Signaling	#4060S, RRID:AB_2315049	WB (1:1000)

p-AKT^{T308}	Cell Signaling	#9275, RRID:AB_329828	WB (1:1000)
AKT	Cell Signaling	#9272S, RRID:AB_329827	WB (1:1000)
ALFA	NanoTag Biotechnologies	#N1581, RRID:AB_3075997	WB (1:1000), IF (1:100)
Transferrin Receptor (Tfr)	DSHB	#G1/221/12, RRID:AB_2201506	WB (1:1000)
EGF Receptor (EGFR) (1005)	Santa Cruz	#sc-03-G	WB (1:500)
GAPDH (D4C6R)	Cell Signaling	#97166S, RRID:AB_2756824	WB (1:5000)
LAMP1	DSHB	#H4A3, RRID:AB_2296838	IF (1:50)
LAMP1 (D2D11)	Cell Signaling	#9091, RRID:AB_2687579	IF (1:800)
NEDD4L	Abcam	# ab46521, RRID:AB_2149325	WB (1:1000)
NEDD4	Cell Signaling	#5344S, RRID:AB_10560514	WB (1:1000)
WWP1	Cell Signaling	#70140S, RRID:AB_3662810	WB (1:1000)
WWP2	Cell Signaling	#41182S, RRID:AB_3662809	WB (1:1000)
ITCH	Cell Signaling	#12117S, RRID:AB_2797822	WB (1:1000)
GFP	Roche Life Science	(Roche Cat# 11814460001, RRID:AB_390913)	WB (1:1000)
Ubiquitin (P4D1)	Santa Cruz	#3936S, RRID:AB_628423	WB (1:500)
Goat anti-mouse IgG-peroxidase	Sigma-Aldrich	#A4416, RRID:AB_258167	WB (1:5000)
Goat anti-rabbit IgG-peroxidase	Sigma-Aldrich	#A0545, RRID:AB_257896	WB (1:5000)
Alexa Fluor 488 goat anti-rabbit IgG	Invitrogen	#A11008, RRID:AB_143165	IF (1:500)
Alexa Fluor 488 goat anti-mouse IgG	Invitrogen	#A11001, RRID:AB_2534069	IF (1:500)
Alexa Fluor 568 goat anti-mouse IgG	Invitrogen	#A11031, RRID:AB_144696	IF (1:500)
Alexa Fluor 568 goat anti-rabbit IgG	Invitrogen	#A11011, RRID:AB_143157	IF (1:500)
NBR1	Santa Cruz Biotechnology	Cat# sc-130380, RRID:AB_2149402	WB (1:1000)
P62	Cell Signaling	Cat# 23214, RRID:AB_2798858	WB (1:1000)
TAX1BP1	Sigma-Aldrich	Cat# HPA024432, RRID:AB_1857783	WB (1:1000)

NDP52	Sigma-Aldrich	Cat# HPA023195, RRID:AB_1845916	WB (1:1000)
Vinculin	Cell Signaling	Cat# 4650, RRID:AB_10559207	WB (1:1000)
**Chemicals**			
Blasticidin	InvivoGen	#ant-bl-5b	
High glucose Dulbecco's modified Eagle's medium (DMEM)	Sigma-Aldrich	#D6429	
FSB	Sigma-Aldrich	#S0615	
Trypsin-EDTA solution	Sigma-Aldrich	#T4174	
MK2206	Enzo Life Sciences	#ENZ-CHM164-0005	
PD0325901	Absource	#S1036	
JPH203	Selleckchem,	#S8667	
chloroquine	Sigma-Aldrich	#C6628	
dynasore hydrate	Sigma-Aldrich	#D7693	
penicillin/streptomycin	Sigma-Aldrich	#P0781	
WesternBright Chemiluminescence substrate solution (Advansta)	Biozym	#541005X	
Lipofectamin LTX reagent	Thermo Scientific	#15338-100	
polyethylenimine (PEI)	Polyscience	#23966-100	
Polybrene	Sigma-Aldrich	#107689	
DAPI	Sigma-Aldrich	#D9542	
Mowiol	Sigma-Aldrich	#81381	
saponin	BioChemika	#84510	
RNAse A	Thermo Scientific	#EN0531	
[$^{14}C_5$]-L-glutamine	Hartmann Analytic	#MC1124	
[4,5-^{3}H]-L-Leucine	Hartmann Analytic	#MT672E	
[$^{14}C_5$]-L-Isoleucine	Hartmann Analytic	#MC174	
glutamine-free DMEM	Sigma-Aldrich	#D6546	
DMEM without L-methionine, L-cysteine and L-glutamine	Sigma-Aldrich	Car #D0422	
AA free DMEM	USBiological	#D9811-01	
2-NBDG	AAT Bioquest	#36702	
phloretin	Sigma-Aldrich	#P7912	
stable isotope-labeled canonical AA mix composition	Cambridge Isotope Laboratory	#MSK-CAA-1	
Cycloheximide	Sigma	Cat. # C7698	
anti-ALFA selector ST magnetic beads	NanoTag Biotechnologies	Cat. # N1516	
**Critical commercial assays**			

Micro BCA Protein Assay Kit	Thermo Scientific	#23235	
RNeasy Mini Kit	Quiagen	#74104	
LunaScript RT Super Mix Kit	NEB	#E3010	
Biozym Blue S'Green qPCR Kit	Biozym	#F-415S	
**Software, algorithm**			
Image J		Version 1.53t, RRID:SCR_003070	
Affinity Designer	Serfi	Version 1.10.5, RRID:SCR_016952	
GraphPad Prism 9		Version 9.4.1, RRID:SCR_002798	
Benchling		RRID:SCR_013955	
Flow Jo		Version 10.8.1, RRID:SCR_008520	
ModFit LT		RRRID:SCR_016106	
Primer3		Version 4.1.0, RRID:SCR_003139	
PrimerBank		RRID:SCR_006898	
CHOPCHOP		RRID:SCR_015723	
ZEISS ZEN Digital Imaging for Light Microscopy		Version 3.5, RRID:SCR_013672	
PrimerQuest^{TM} Tool	Integrated DNA Technologies, IDT		
ICE Analysis online tool	Synthego, USA	Synthego, v2.0	
IncuCyte S3 software	Sartorius	Version 2022B	
Wave Pro software	Agilent Technologies	Version 10.2.1.4	
Proteome Discoverer	Thermo Fischer Scientific	Version 3.1	

Appendix Table S3: List of oligonucleotides used in this study

**Name**	**Vector backbone**	**Origin**
TXNIP	pCCL-EIF1α-BlastiR-DEST	This study
ALFA-TXNIP	pCCL-EIF1α-BlastiR-DEST	This study
SLC7A5-ALFA	pCCL-EIF1α-BlastiR-DEST	This study
$TXNIP^{S308A}$/ ALFA-$TXNIP^{S308A}$	pCCL-EIF1α-BlastiR-DEST	This study
$TXNIP^{S308D}$/ALFA-$TXNIP^{S308D}$	pCCL-EIF1α-BlastiR-DEST	This study
$TXNIP^{C247S/}$ALFA-$TXNIP^{C247S}$	pCCL-EIF1α-BlastiR-DEST	This study
TXNIP p.Ile215TyrfsTer59 /ALFA-TXNIP p.Ile215TyrfsTer59	pCCL-EIF1α-BlastiR-DEST	This study
TXNIP p.Gln58HisGly59Ter	pCCL-EIF1α-BlastiR-DEST	This study
ALFA-$TXNIP^{PPxY331AAxA,PPxY375AAxA}$	pCCL-EIF1α-BlastiR-DEST	This study
ALFA-TXNIPPPCY331AACA	pCCL-EIF1α-BlastiR-DEST	This study
EGFP-TXNIP	pCCL-EIF1α-BlastiR-DEST	This study
mCherrry	pCCL-EIF1α-BlastiR-DEST	This study
pVSV-G		Clonetech, #631530
psPAX2		Gift from Stephan Geley
pDONR-ALFA-MCS		Generated by Michael Widerin
pDONR-MCS-ALFA		Generated by Michael Widerin
pDONR-221		Invitrogen, #12536017
pCCL-EIF1α-BlastiR-DEST		Gift from Stephan Geley
pSpCas9(BB)-2A-GFP (PX458)		Addgene, #48138
YFP-NEDD4	pCR3.1	(Woelk *et al*, 2006), (Gahlot *et al*, 2024)
YFP-ITCH	pCR3.1	(Woelk *et al.*, 2006), (Gahlot *et al.*, 2024)

YFP-WWP1	pCR3.1	(Woelk *et al.*, 2006), (Gahlot *et al.*, 2024)
YFP-WWP2	pCR3.1	(Woelk *et al.*, 2006), (Gahlot *et al.*, 2024)
YFP-HECW1	pCR3.1	(Woelk *et al.*, 2006), (Gahlot *et al.*, 2024)
YFP-HECW2	pCR3.1	(Woelk *et al.*, 2006), (Gahlot *et al.*, 2024)

Appendix Table S4: Primers for PCR-based genetic modifications and cloning

Name	Forward primer	Reverse primer
ALFA-TXNIP	tAAgcagAAttcAatggtgatgttcAAgAAgatc	TGCTTAgtcgacTCACTGCACATTGTTGTTGA
attB-TXNIP	CAAAAAAGCAGGCTCCGCCACCATGGTGATGTTCAAGAAGATCAAGTC	CAAGAAAGCTGGGTCTCACTGCACATTGTTGTTGAGG
SLC7A5-ALFA	TAAGCAGTCGACatggcgggtgcgggcccg	TGCTTAGAATTCtgtctcctgggggaccac
Gateway	GGGGACAAGTTTGTACAAAAAAGCAGGCTCC	GGGGACCACTTTGTACAAGAAAGCTGGGTC
TXNIP^{S308A}	gcagAAcatccGCGatggccag	gttcggctggccatCGCggatgttctgc
TXNIP^{S308D}	gcagAAcatccGACatggccagccg	gttcggctggccatGTCggatgttctgctg
TXNIP^{C247S}	tattatctcagggacaAGCgcatcatggcgtggc	ccacgccatgatgcGCTtgtccctgagatAAtatgattgcc
TXNIPp.Ile215TyrfsTer59	agctgccTattgtggcccgccacacttac	gtAAgtgtggcgggccacAAtAggcagct
TXNIP^{PPxY331AAxA}	ataccccagAAgctGCAGCAtgcGCAatggatgtcattcc	caggAAtgacatccatTGCgcaTGCTGCagcttctggggtatc
TXNIP^{PPxY375AAxA}	ttcAAgttcatgccaGCAGCAactGCAactgaggtggatccctgc	gcagggatccacctcagtTGCagtTGCTGCtggcatgAActtgAA
ALFA-TAG	caggggtattgacatccaccagat	atctggtggatgtcAAtacccctg
TXNIPp.Gln58HisGly59*	gtgctttggatgcaTTgatcccagcagtgc	gcactgctgggatcAAtgcatccaaagcac
GFP-TXNIP	taagcagaattcAatggtgatgttcaagaagatc	TGCTTAgtcgacTCACTGCACATTGTTGTTGA
mCherry	cAAAAAGCAGGCTCCACCatggtgagcaagggcgaggaggat	caagaaagctgggtcAATcagctcgtccatgcc

Appendix Table S5: qPCR Primers used in this study

Gene	Forward primer	Reverse primer	Source
RPLP0	TGGTCATCCAGCAGGTG TTCGA	ACAGACACTGGCAAC ATTGC GG	(Krzystek-Korpacka *et al*, 2016)
SLC7A5	ATCGGGAAGGGTGATG TGTCCAAT	CAAAGAGGCCGCTGT ATAATGCCA	(Krzystek-Korpacka *et al.*, 2016)
ARRDC1	TAGTGGAGGAGGGTTA CTTCAAC	TCTGGGATGCTGTTC AGGTTC	PrimerBank ID (65288282c1)
ARRDC2	GATTTTGCGTTCTGAGG CTG	GCTTTCACCTTGTCG AATAGC	IDT
ARRDC3	TCTTGCCTCCACCTCTTT ATTC	TCGGAACCCACATCA ACTTG	Krzystek-Korpacka *et al.*, 2016)
ARRDC4	GTTCCTCTCGTCTGATT GTTCC	ATTGGCGACCATGTG TCGAAT	(Kaira *et al*, 2013)
TXNIP	GAGTACCTGCGCTATGA AGAC	TTTGAAGGATGTTCC CAGAGG	PrimerBank ID (65288282c1)

Appendix References

Gahlot P, Kravic B, Rota G, van den Boom J, Levantovsky S, Schulze N, Maspero E, Polo S, Behrends C, Meyer H (2024) Lysosomal damage sensing and lysophagy initiation by SPG20-ITCH. *Mol Cell* 84: 1556–1569 e1510

Kaira K, Sunose Y, Ohshima Y, Ishioka NS, Arakawa K, Ogawa T, Sunaga N, Shimizu K, Tominaga H, Oriuchi N *et al* (2013) Clinical significance of L-type amino acid transporter 1 expression as a prognostic marker and potential of new targeting therapy in biliary tract cancer. *BMC Cancer* 13: 482

Krzystek-Korpacka M, Hotowy K, Czapinska E, Podkowik M, Bania J, Gamian A, Bednarz-Misa I (2016) Serum availability affects expression of common house-keeping genes in colon adenocarcinoma cell lines: implications for quantitative real-time PCR studies. *Cytotechnology* 68: 2503–2517

Woelk T, Oldrini B, Maspero E, Confalonieri S, Cavallaro E, Di Fiore PP, Polo S (2006) Molecular mechanisms of coupled monoubiquitination. *Nat Cell Biol* 8: 1246–1254
