# Supplementary figures and images for: TXNIP mediates LAT1/SLC7A5 endocytosis to limit amino acid uptake in cells entering quiescence
Source: EMBO J. 2025 Oct 20;44(23):7119–53. doi: 10.1038/s44318-025-00608-9 (PMC12669767; doi:10.1038/s44318-025-00608-9)

Figure 1C

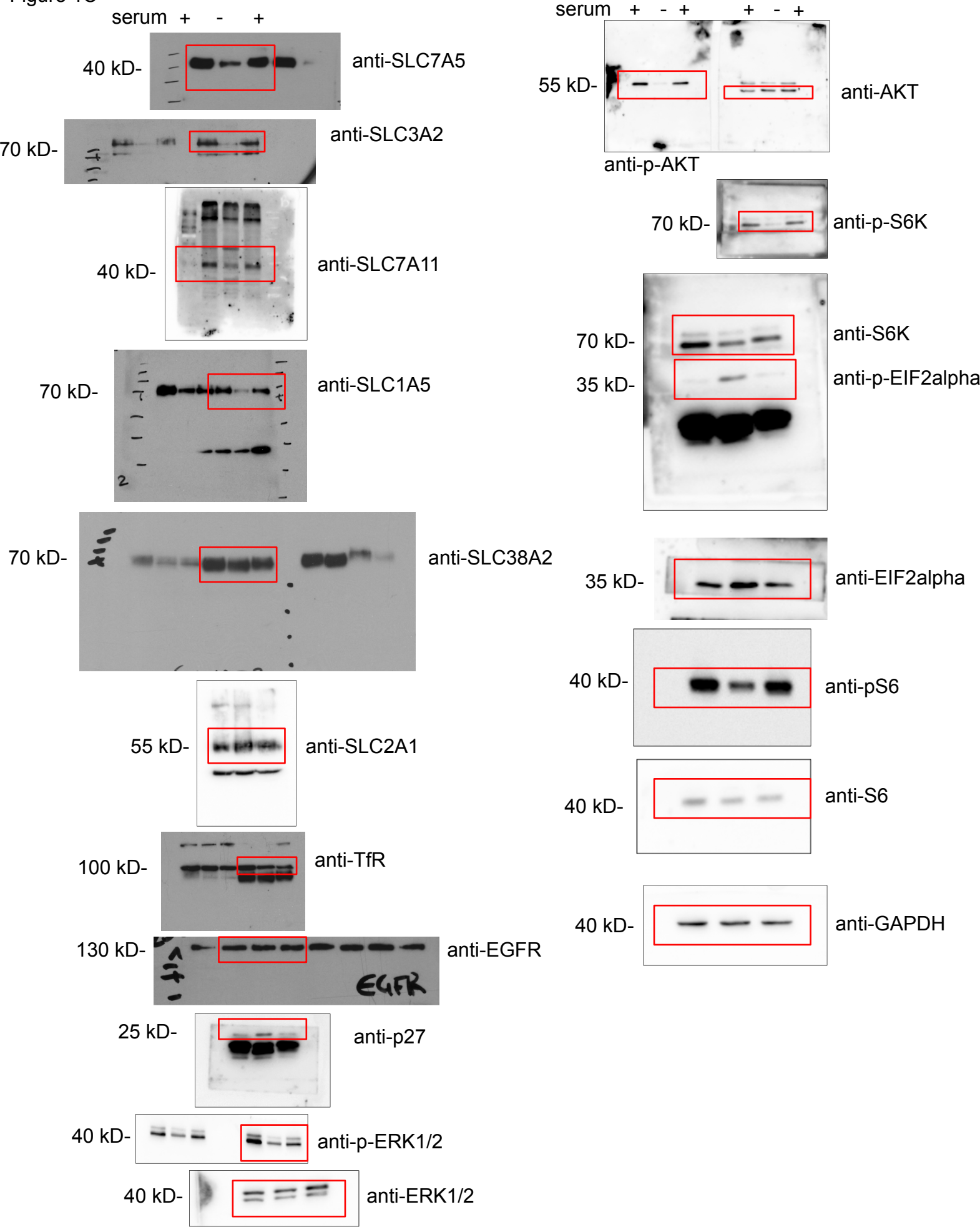

Supplement: Supplementary file 4 — Source data Fig. 1 [file 44318_2025_608_MOESM4_ESM.zip › Figure 1/1C/Figure 1C.pdf]

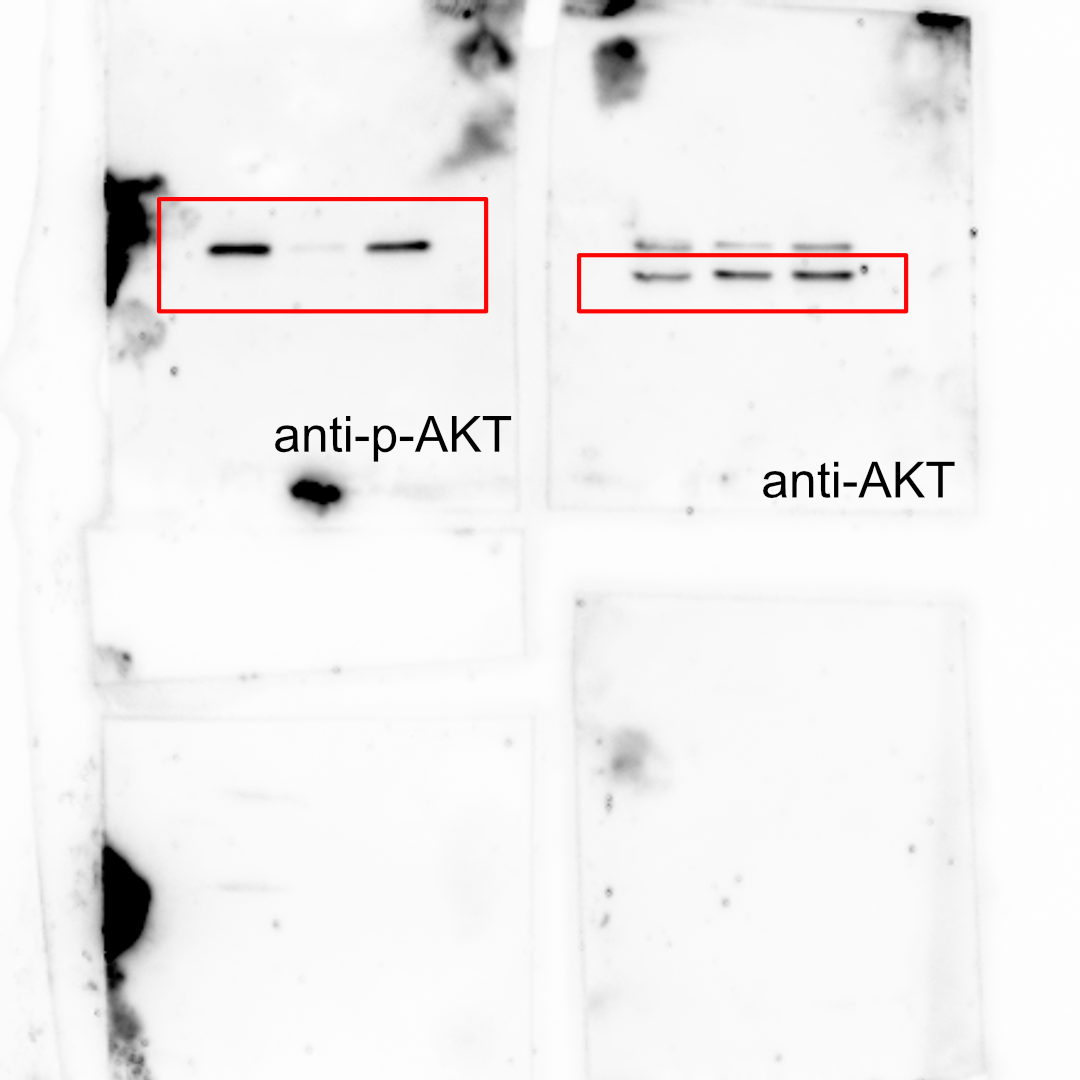

Supplement: Supplementary file 4 — Source data Fig. 1 [file 44318_2025_608_MOESM4_ESM.zip › Figure 1/1C/western AKT_p-AKT.tiff]

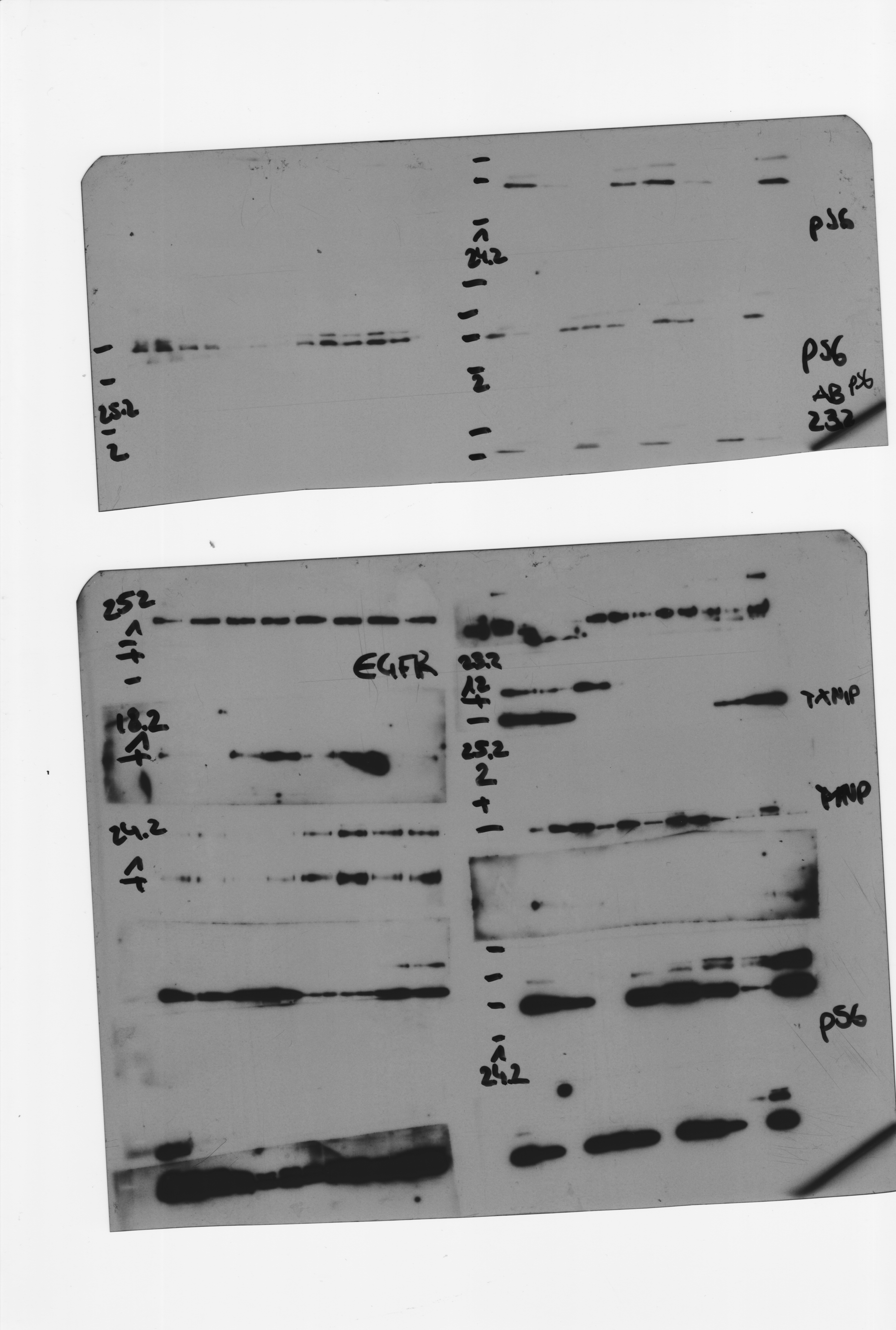

Supplement: Supplementary file 4 — Source data Fig. 1 [file 44318_2025_608_MOESM4_ESM.zip › Figure 1/1C/western EGFR.TIF]

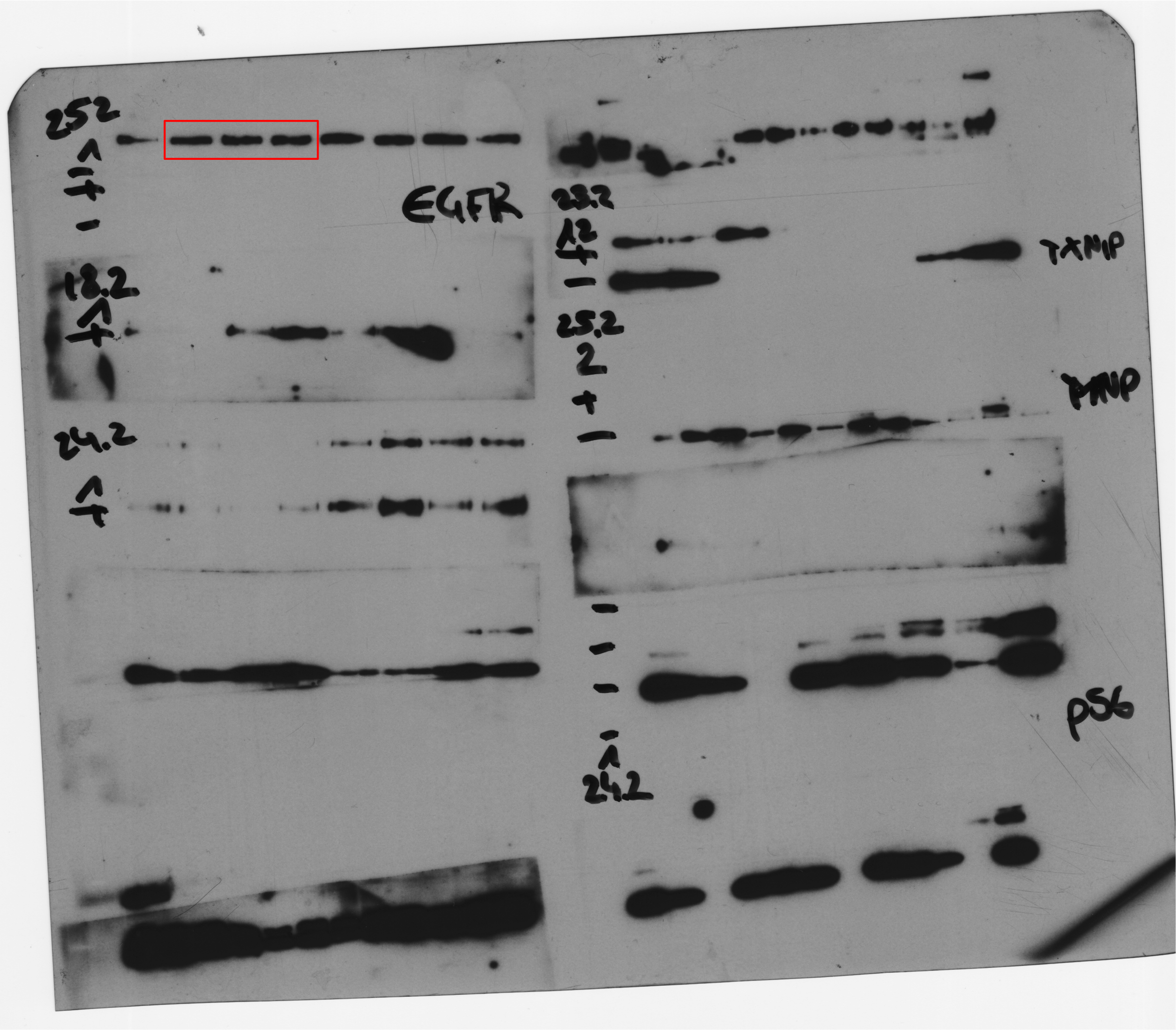

Supplement: Supplementary file 4 — Source data Fig. 1 [file 44318_2025_608_MOESM4_ESM.zip › Figure 1/1C/western EGFR.tiff]

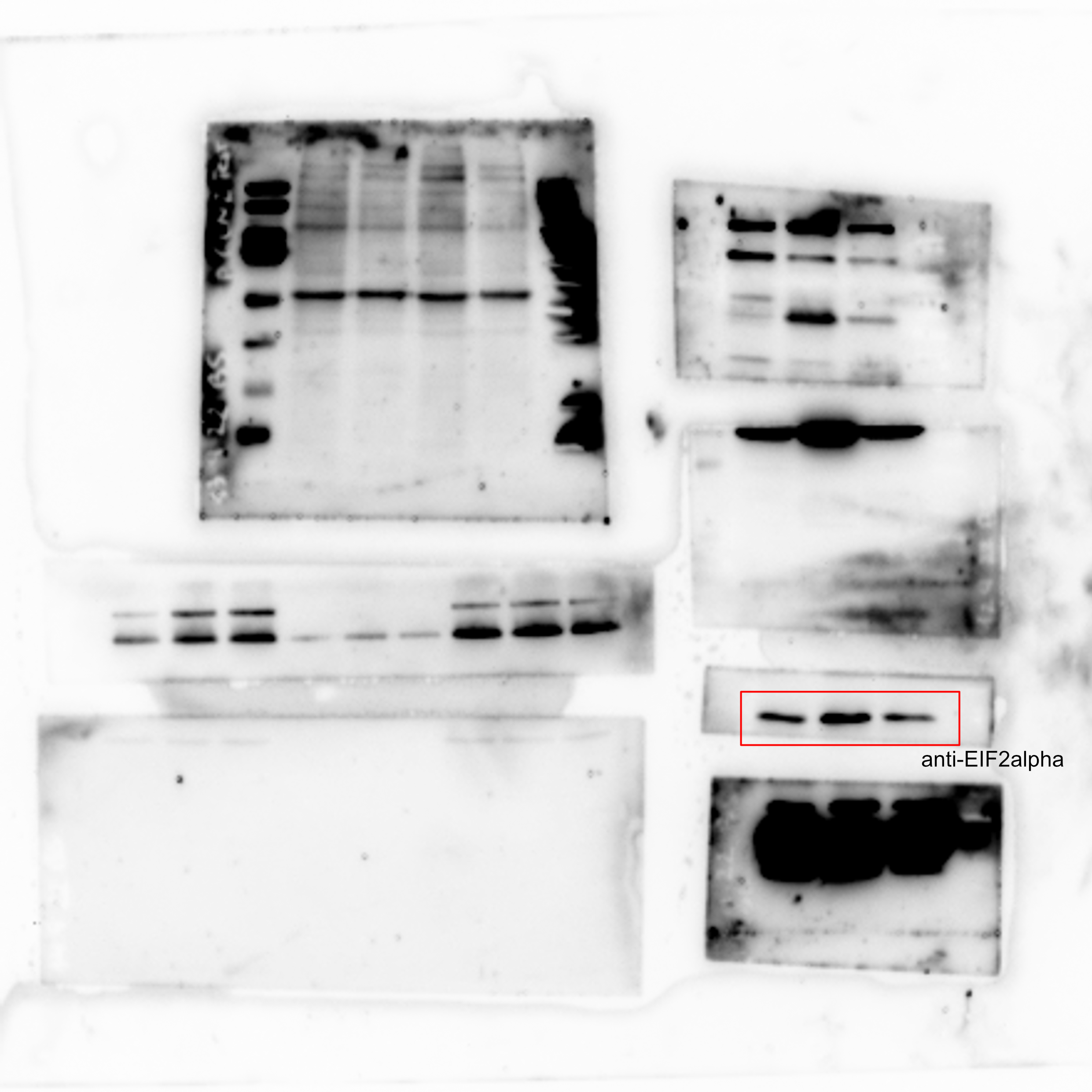

Supplement: Supplementary file 4 — Source data Fig. 1 [file 44318_2025_608_MOESM4_ESM.zip › Figure 1/1C/western EIF2alpha.tiff]

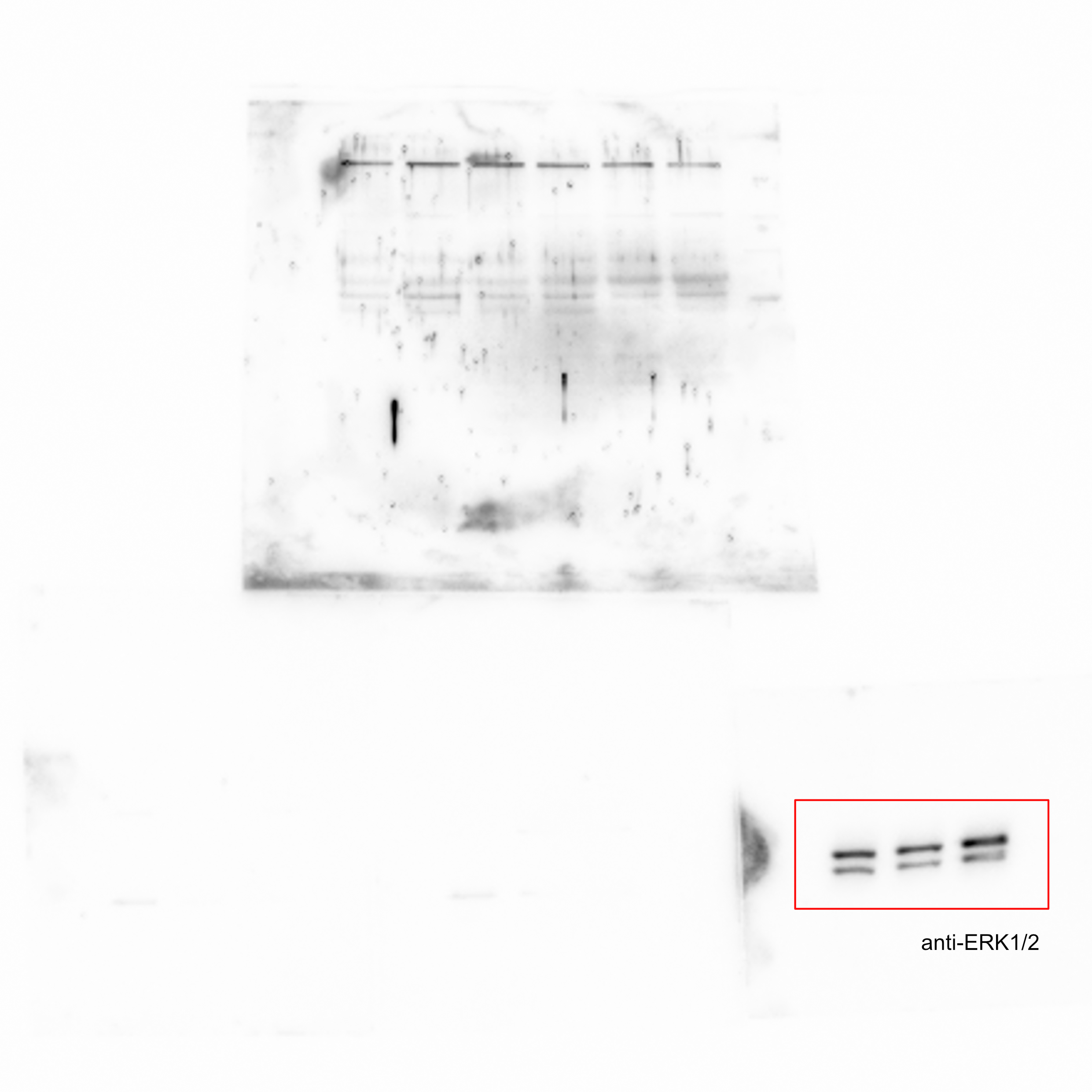

Supplement: Supplementary file 4 — Source data Fig. 1 [file 44318_2025_608_MOESM4_ESM.zip › Figure 1/1C/western ERK.tiff]

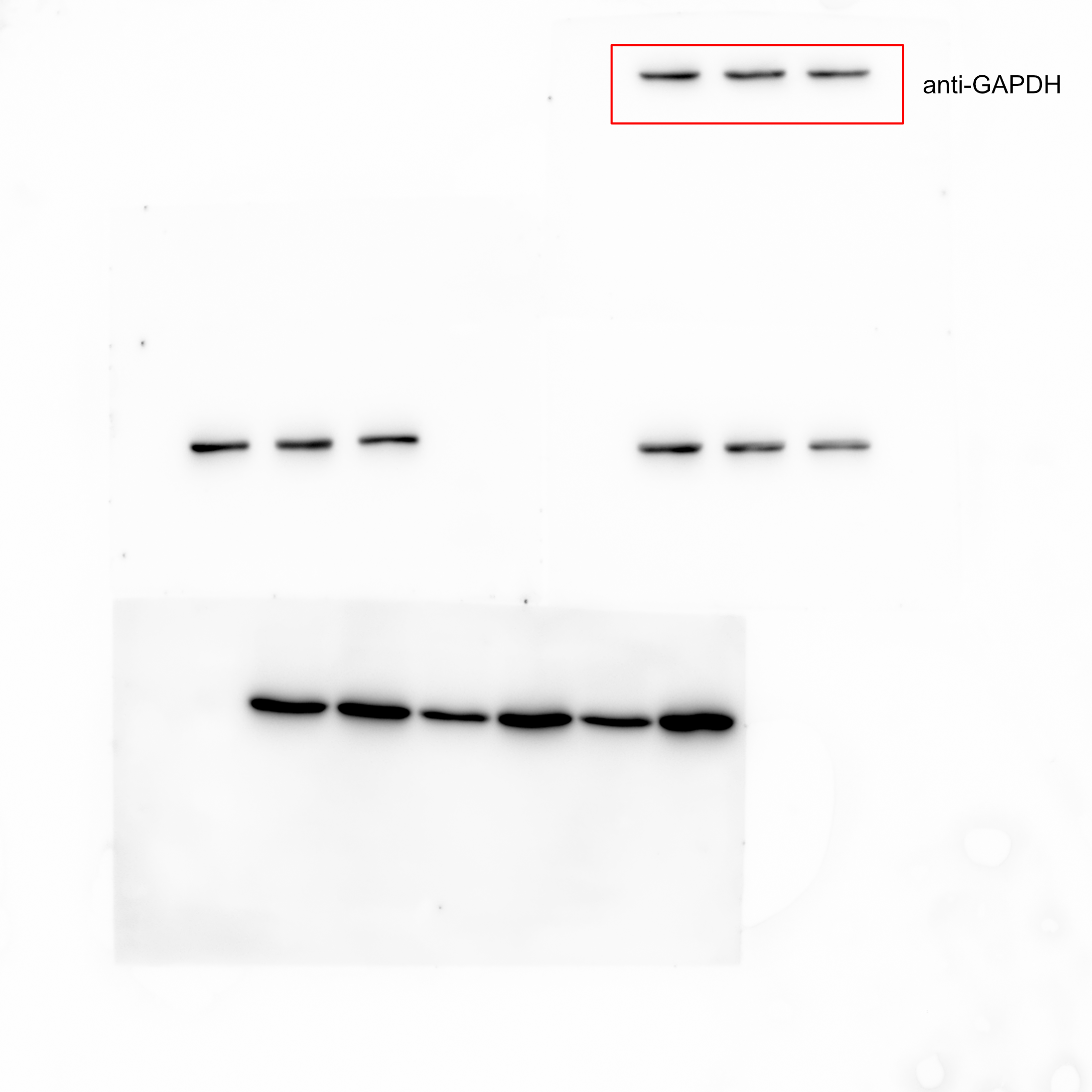

Supplement: Supplementary file 4 — Source data Fig. 1 [file 44318_2025_608_MOESM4_ESM.zip › Figure 1/1C/western GAPDH.tiff]

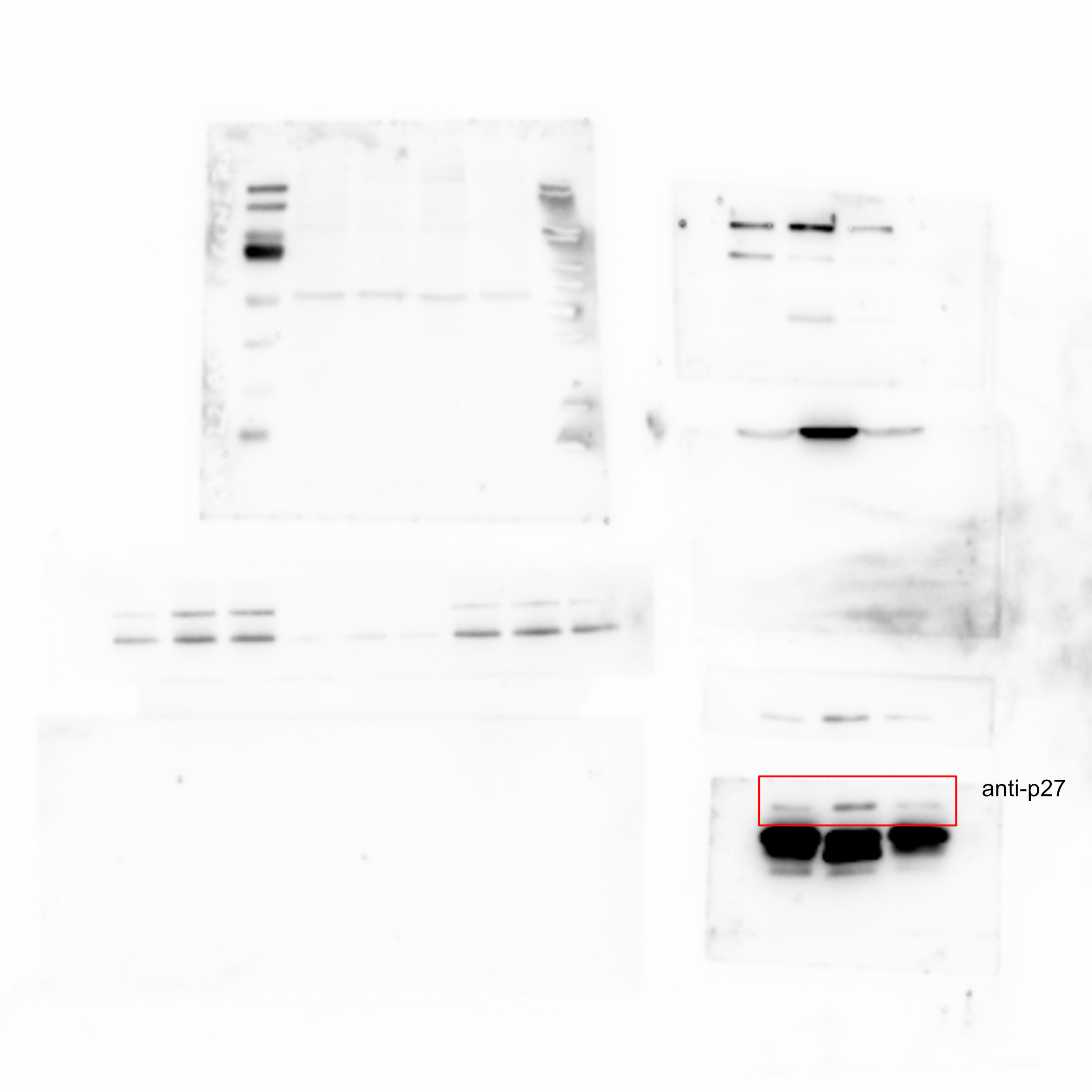

Supplement: Supplementary file 4 — Source data Fig. 1 [file 44318_2025_608_MOESM4_ESM.zip › Figure 1/1C/western p27.tiff]

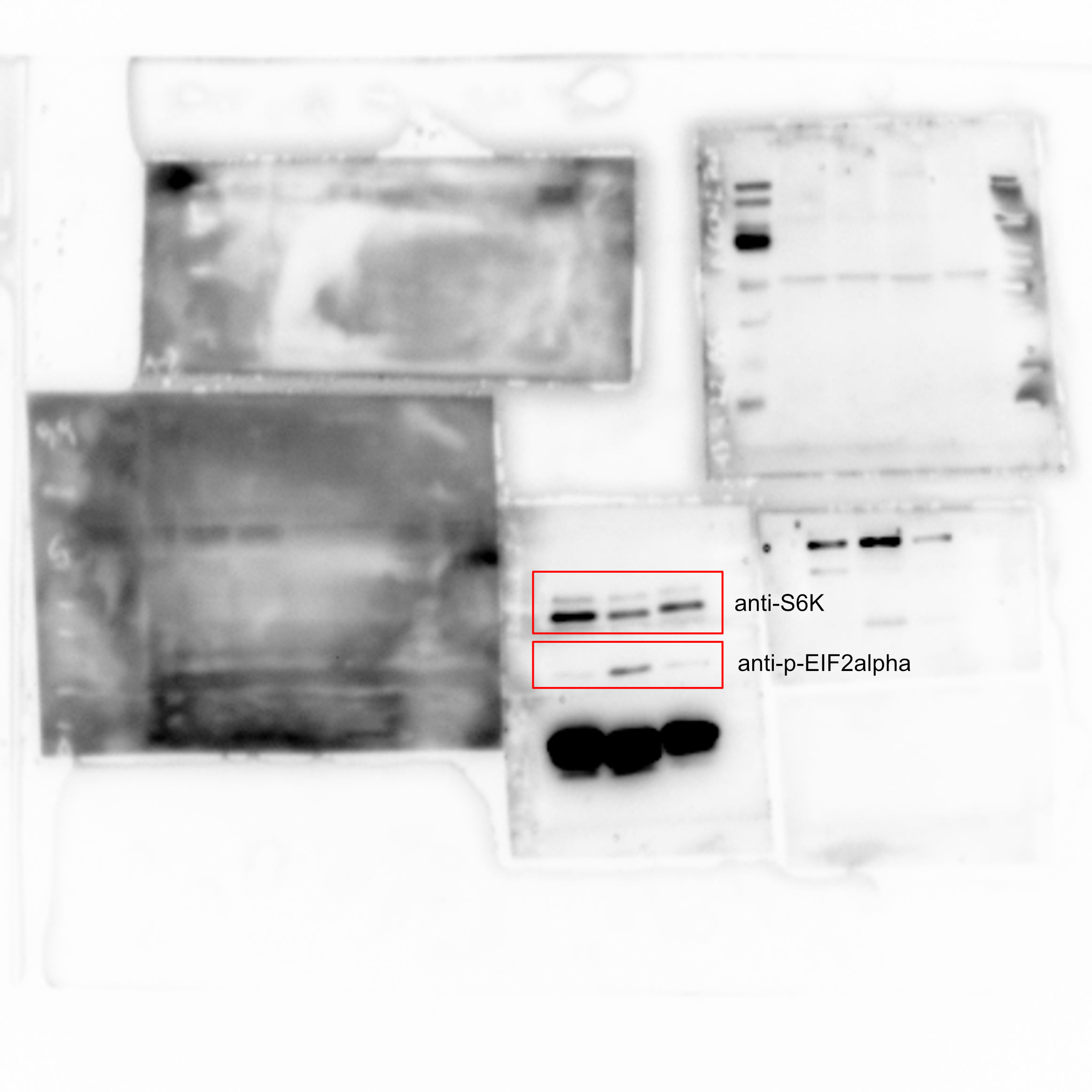

Supplement: Supplementary file 4 — Source data Fig. 1 [file 44318_2025_608_MOESM4_ESM.zip › Figure 1/1C/western pEIF2alpha_S6K.tiff]

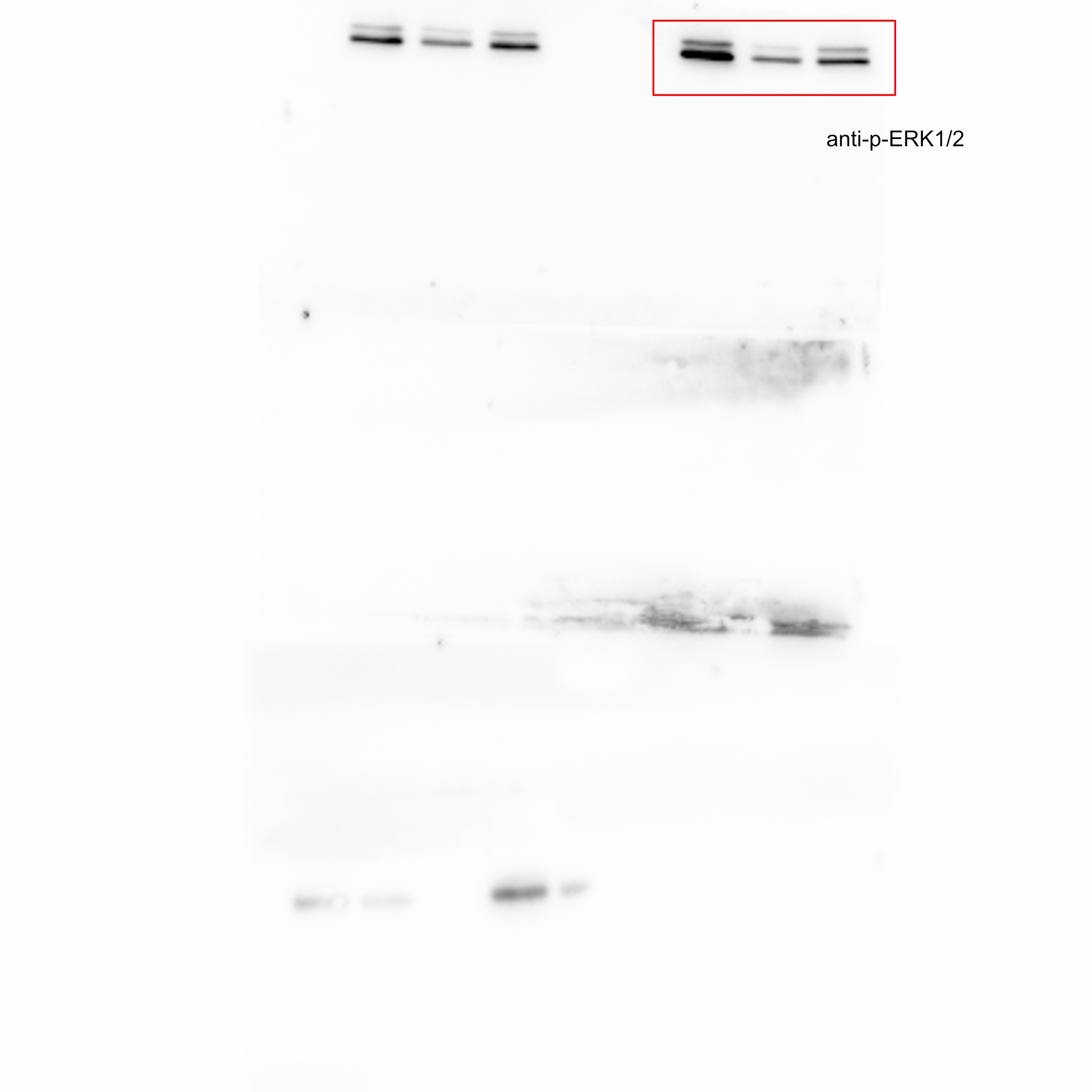

Supplement: Supplementary file 4 — Source data Fig. 1 [file 44318_2025_608_MOESM4_ESM.zip › Figure 1/1C/western pERK1_2.tiff]

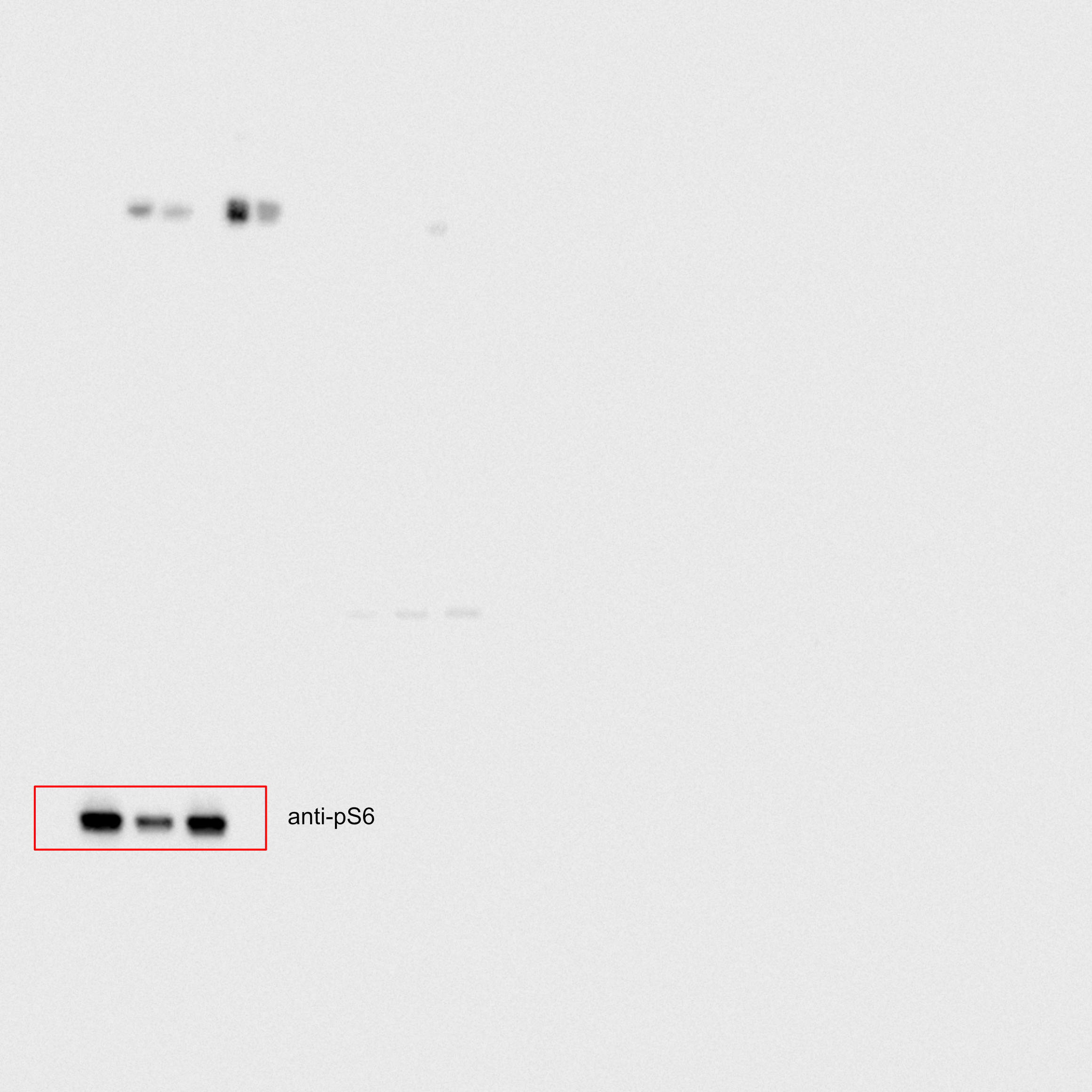

Supplement: Supplementary file 4 — Source data Fig. 1 [file 44318_2025_608_MOESM4_ESM.zip › Figure 1/1C/western pS6.tiff]

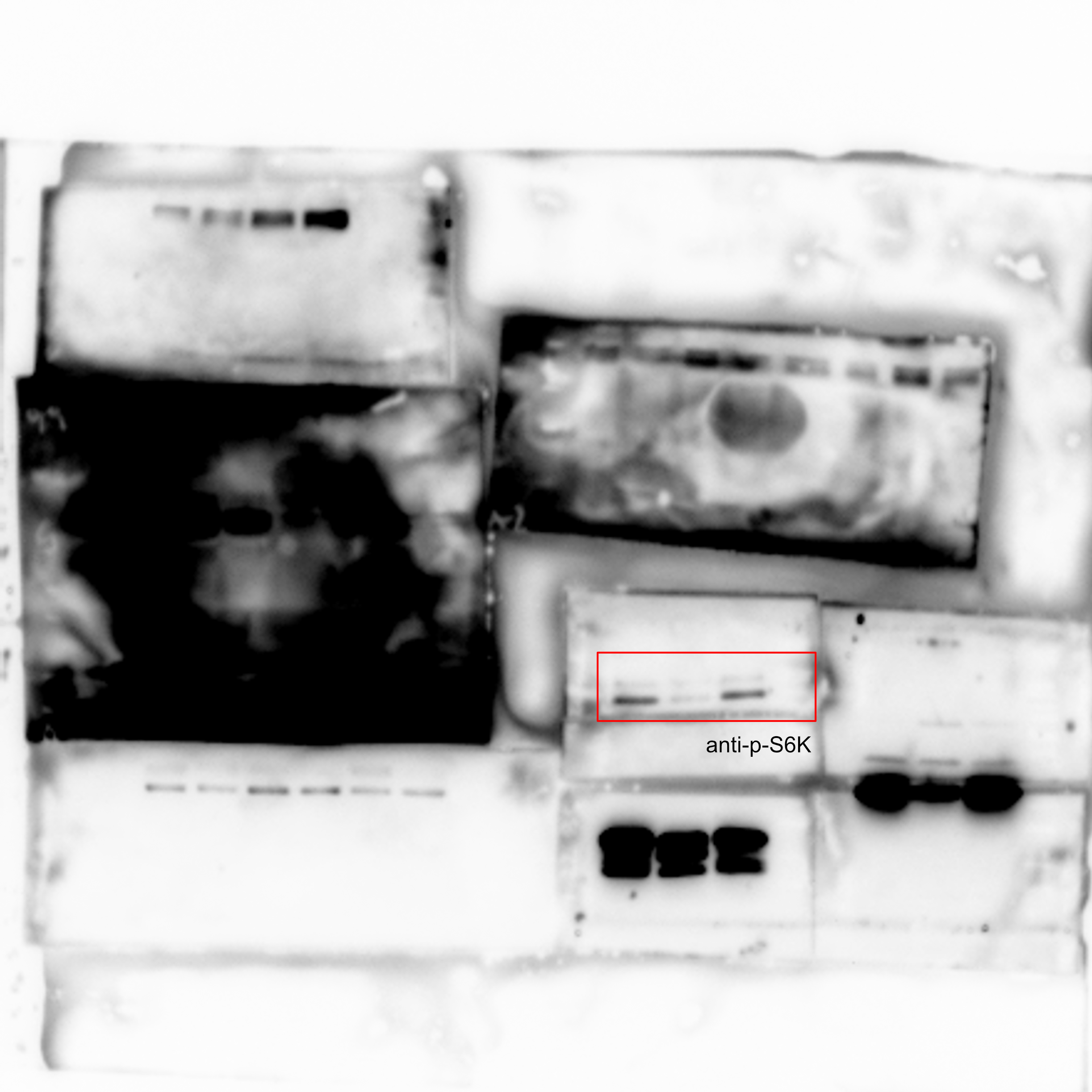

Supplement: Supplementary file 4 — Source data Fig. 1 [file 44318_2025_608_MOESM4_ESM.zip › Figure 1/1C/western pS6K.tiff]

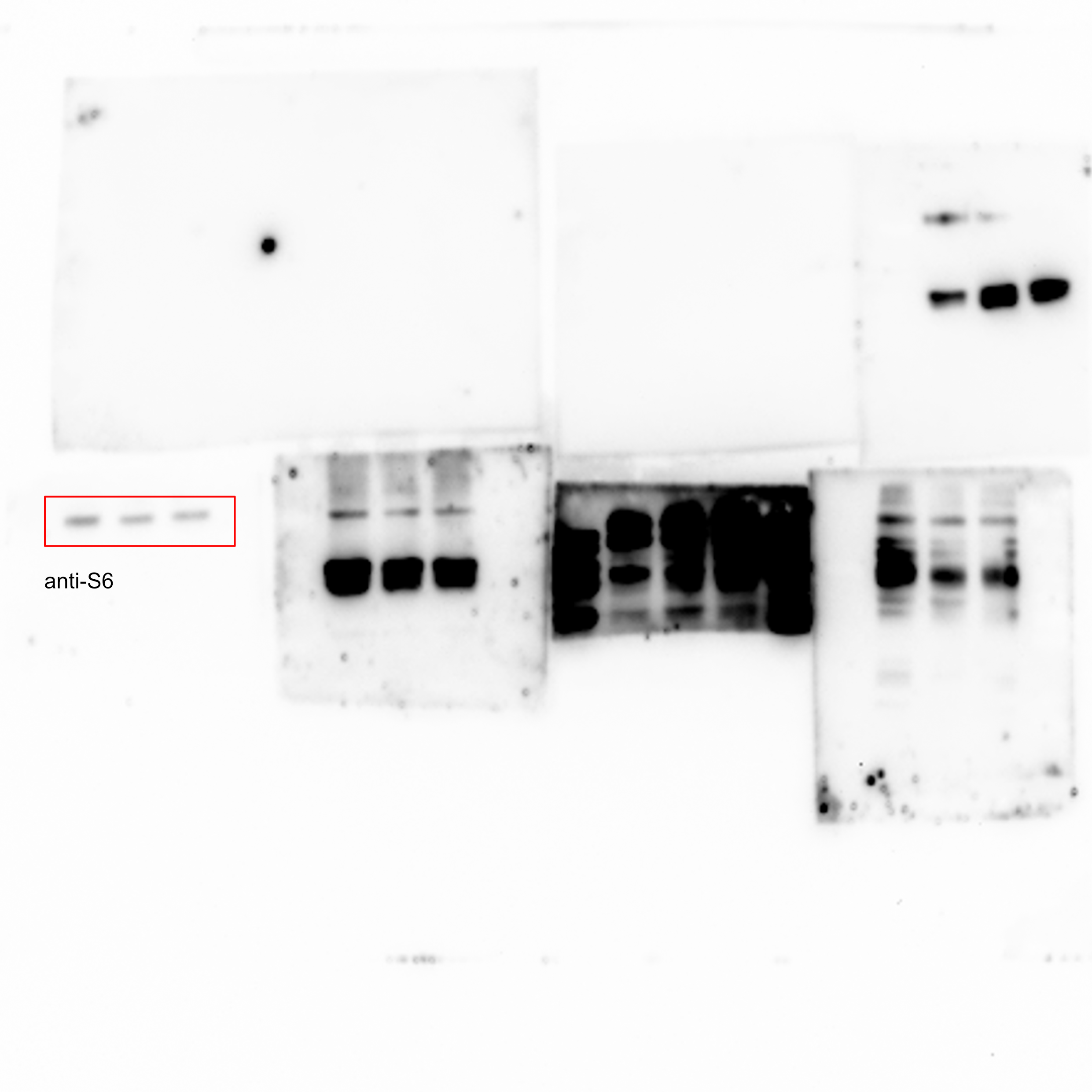

Supplement: Supplementary file 4 — Source data Fig. 1 [file 44318_2025_608_MOESM4_ESM.zip › Figure 1/1C/western S6.tiff]

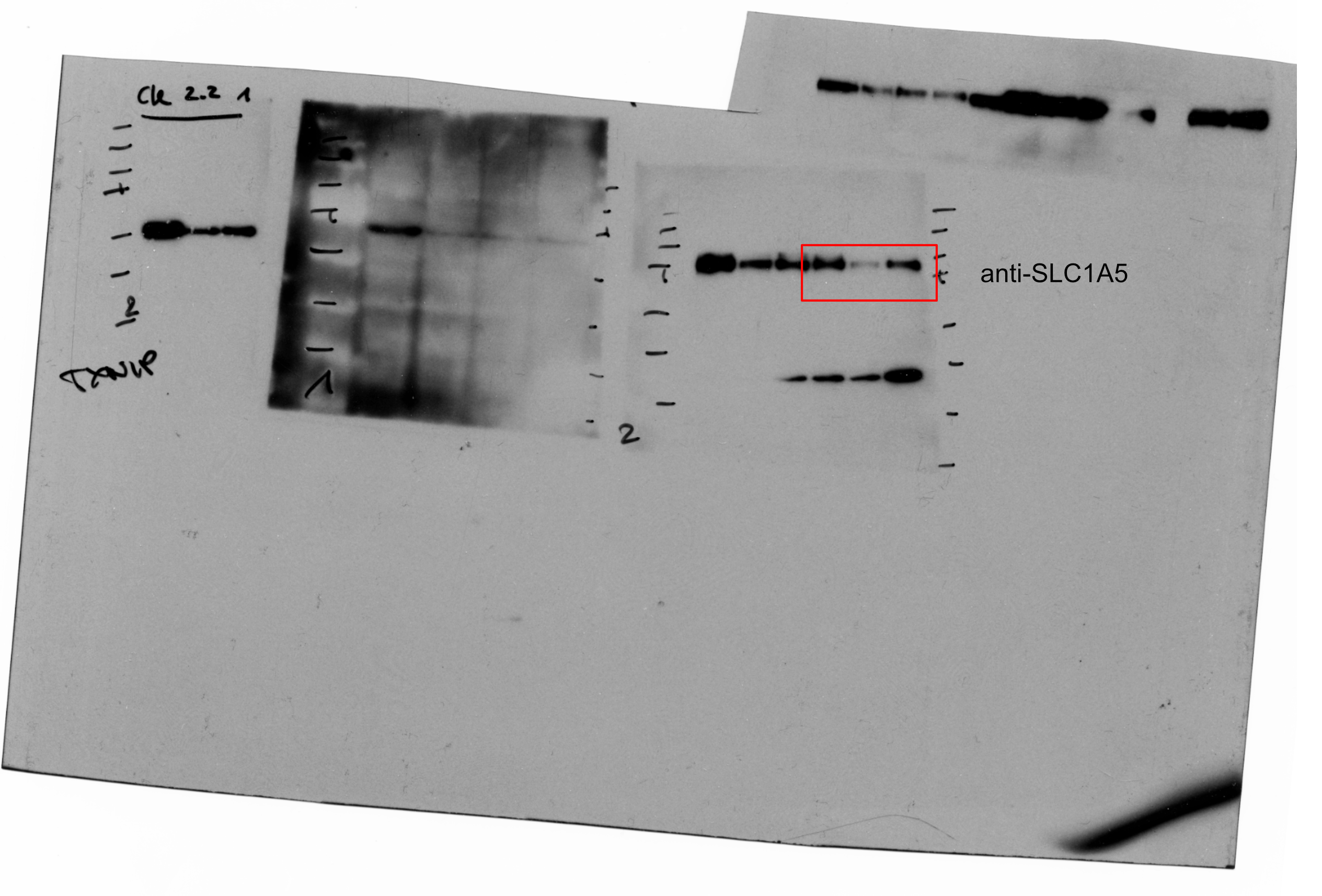

Supplement: Supplementary file 4 — Source data Fig. 1 [file 44318_2025_608_MOESM4_ESM.zip › Figure 1/1C/western SLC1A5.tiff]

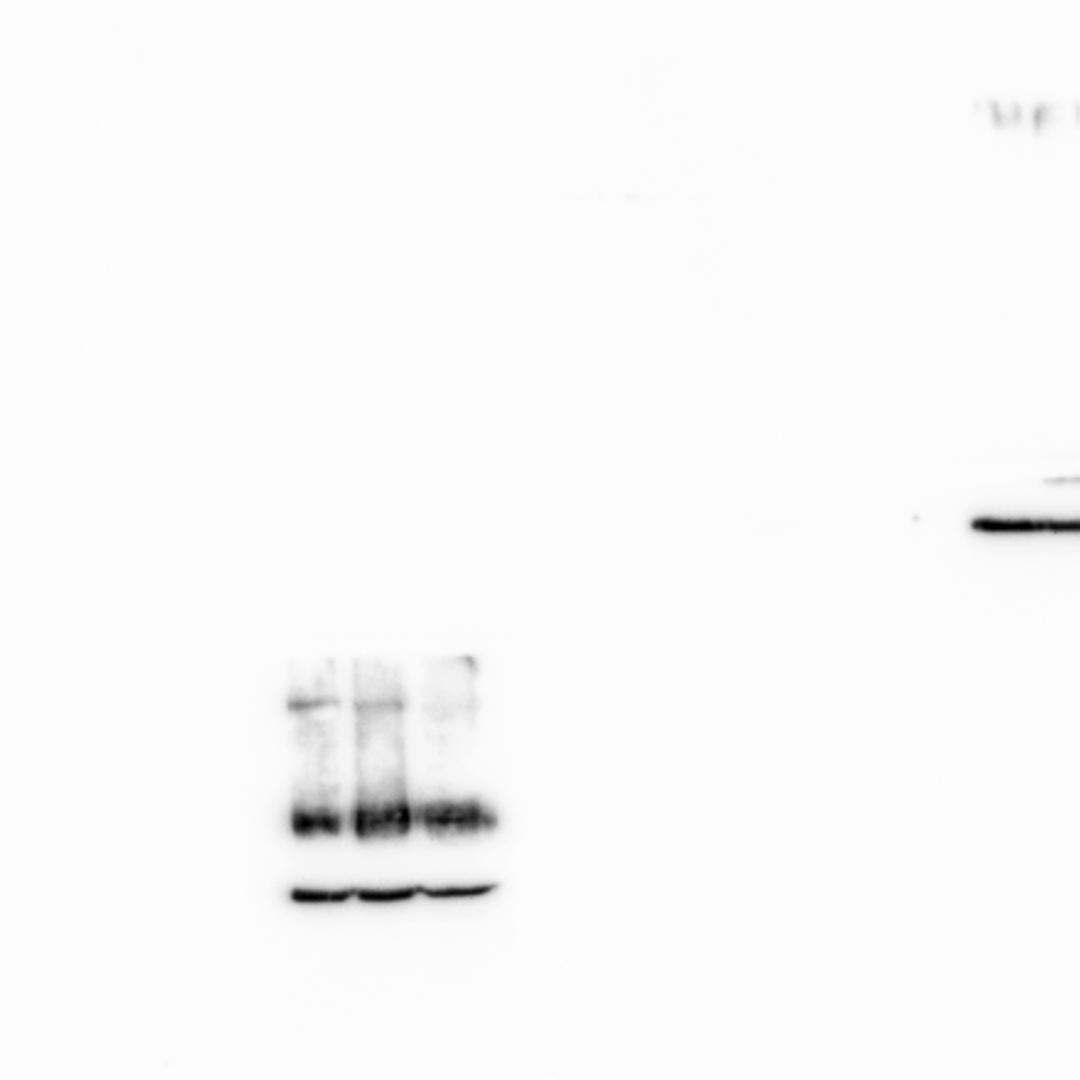

Supplement: Supplementary file 4 — Source data Fig. 1 [file 44318_2025_608_MOESM4_ESM.zip › Figure 1/1C/western SLC2A1.Tif]

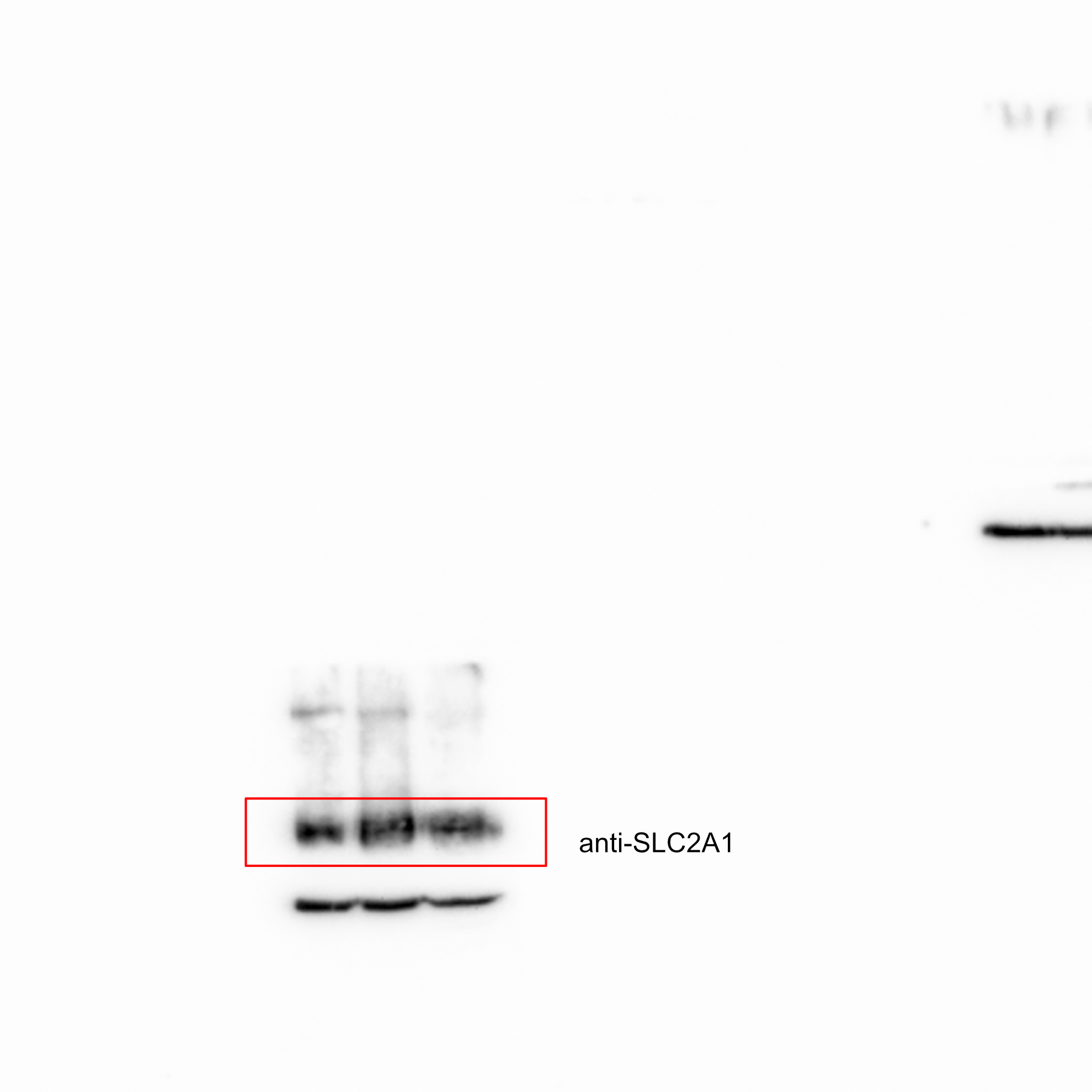

Supplement: Supplementary file 4 — Source data Fig. 1 [file 44318_2025_608_MOESM4_ESM.zip › Figure 1/1C/western SLC2A1.tiff]

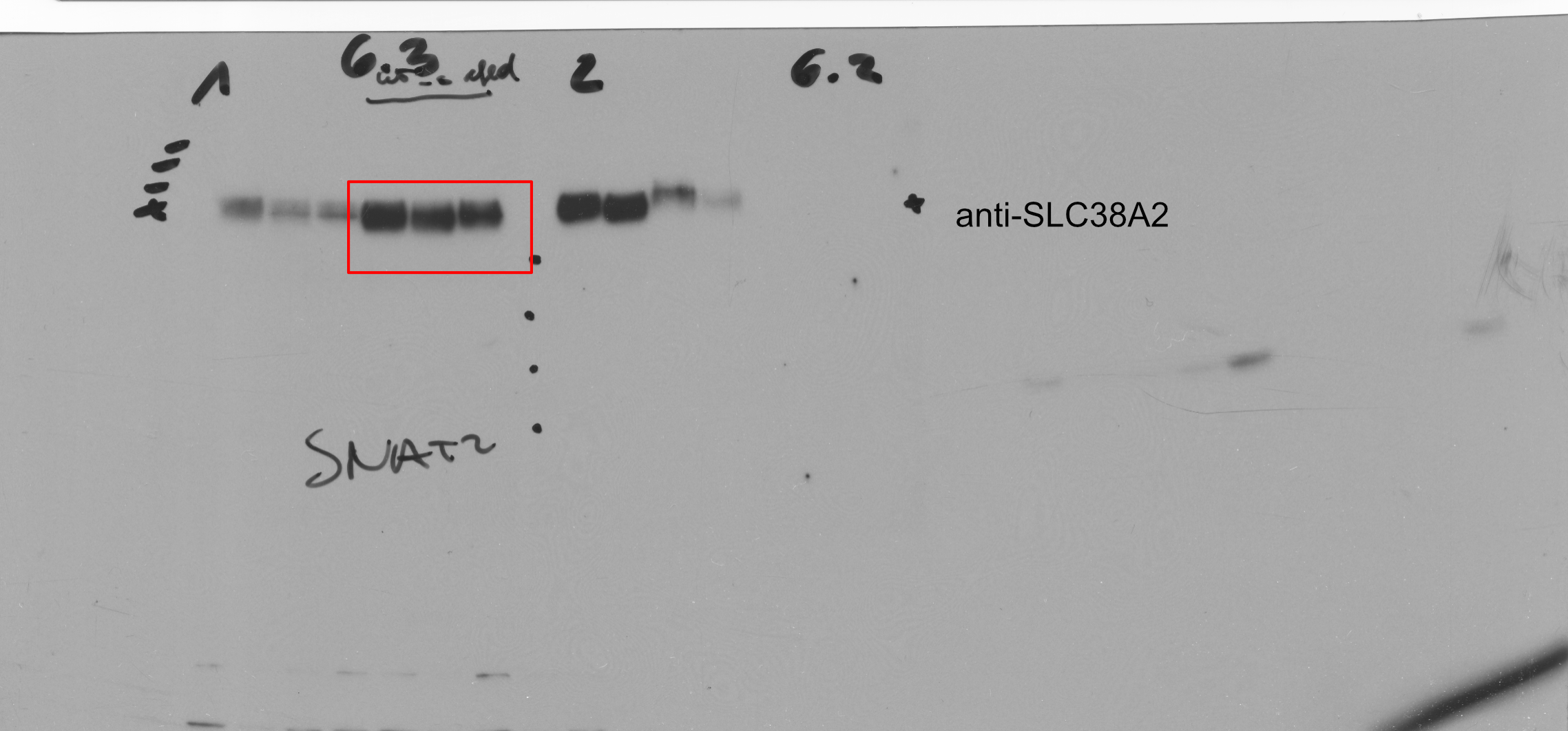

Supplement: Supplementary file 4 — Source data Fig. 1 [file 44318_2025_608_MOESM4_ESM.zip › Figure 1/1C/western SLC38A2.tiff]

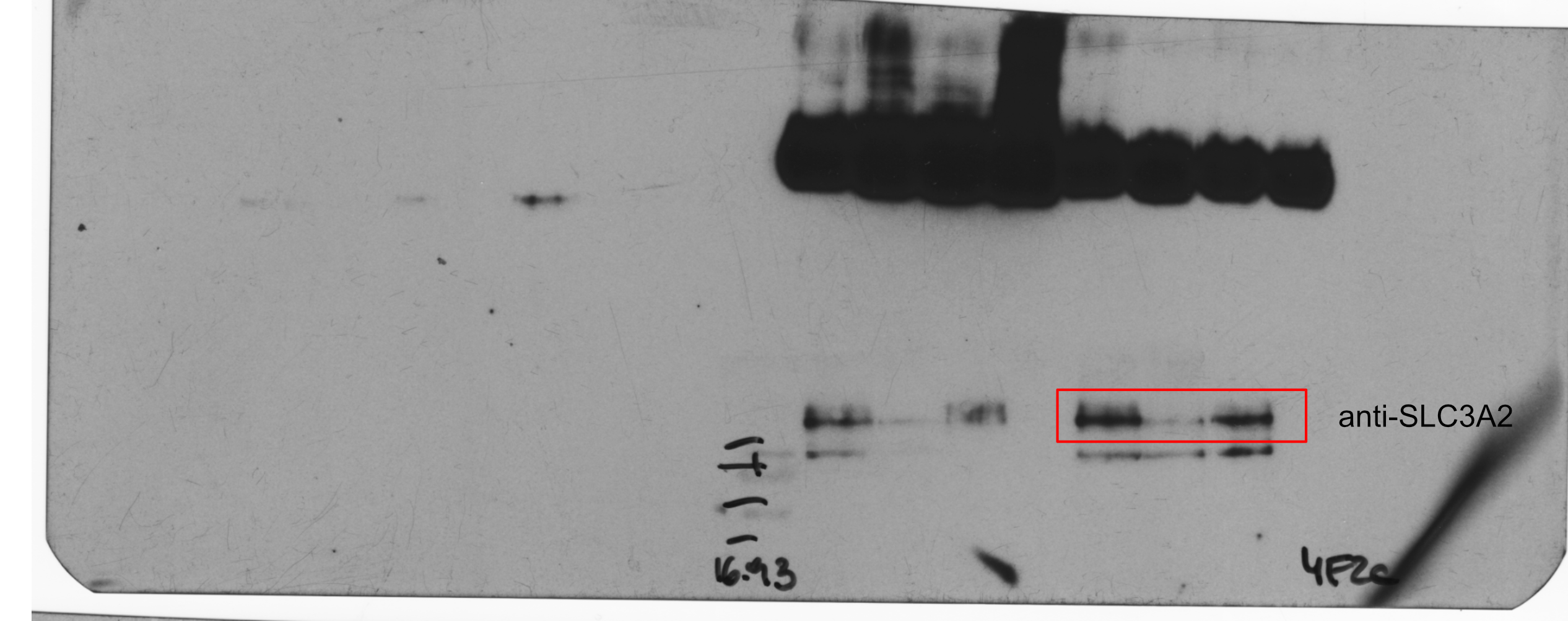

Supplement: Supplementary file 4 — Source data Fig. 1 [file 44318_2025_608_MOESM4_ESM.zip › Figure 1/1C/western SLC3A2.tiff]

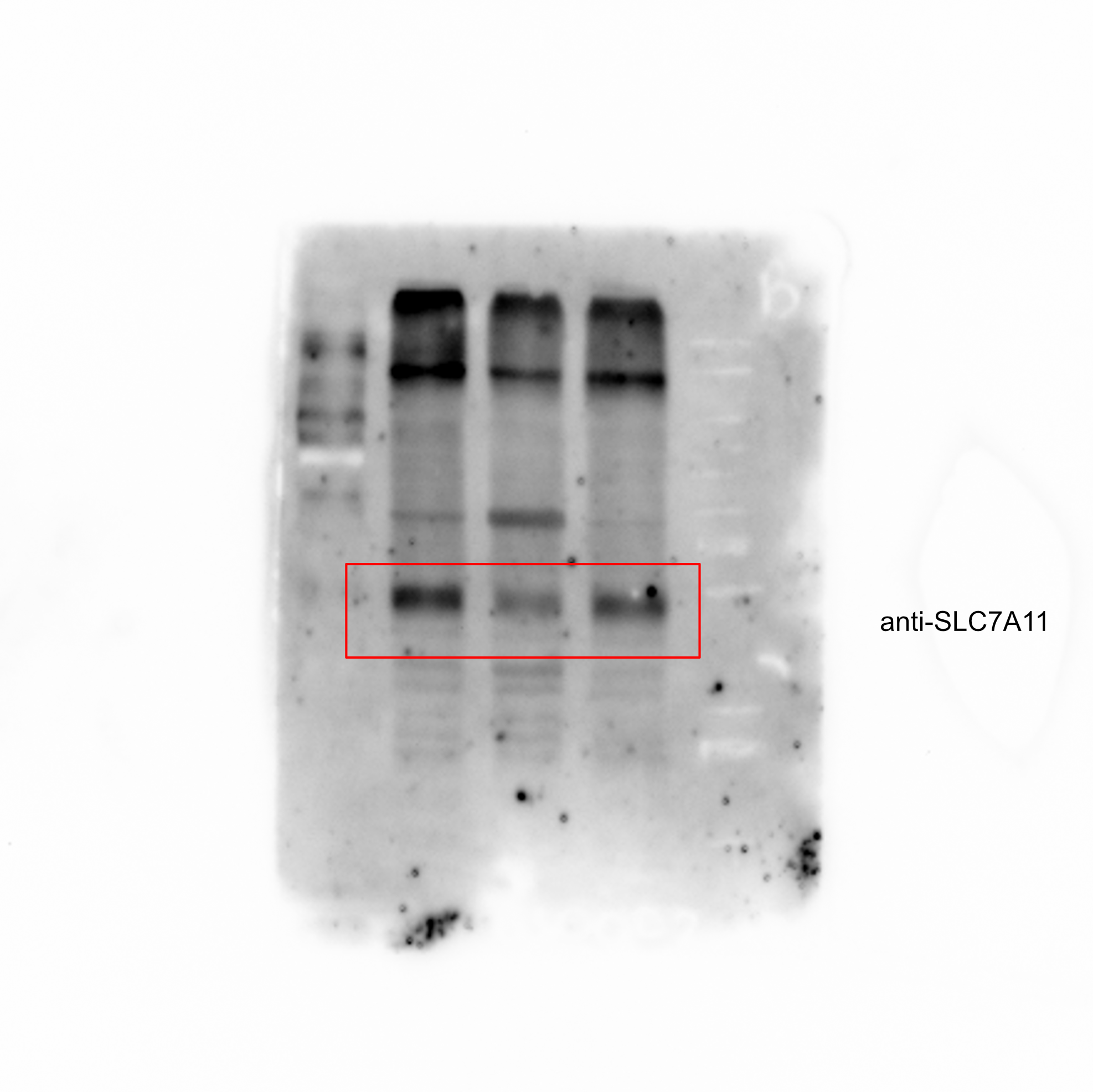

Supplement: Supplementary file 4 — Source data Fig. 1 [file 44318_2025_608_MOESM4_ESM.zip › Figure 1/1C/western SLC7A11.tiff]

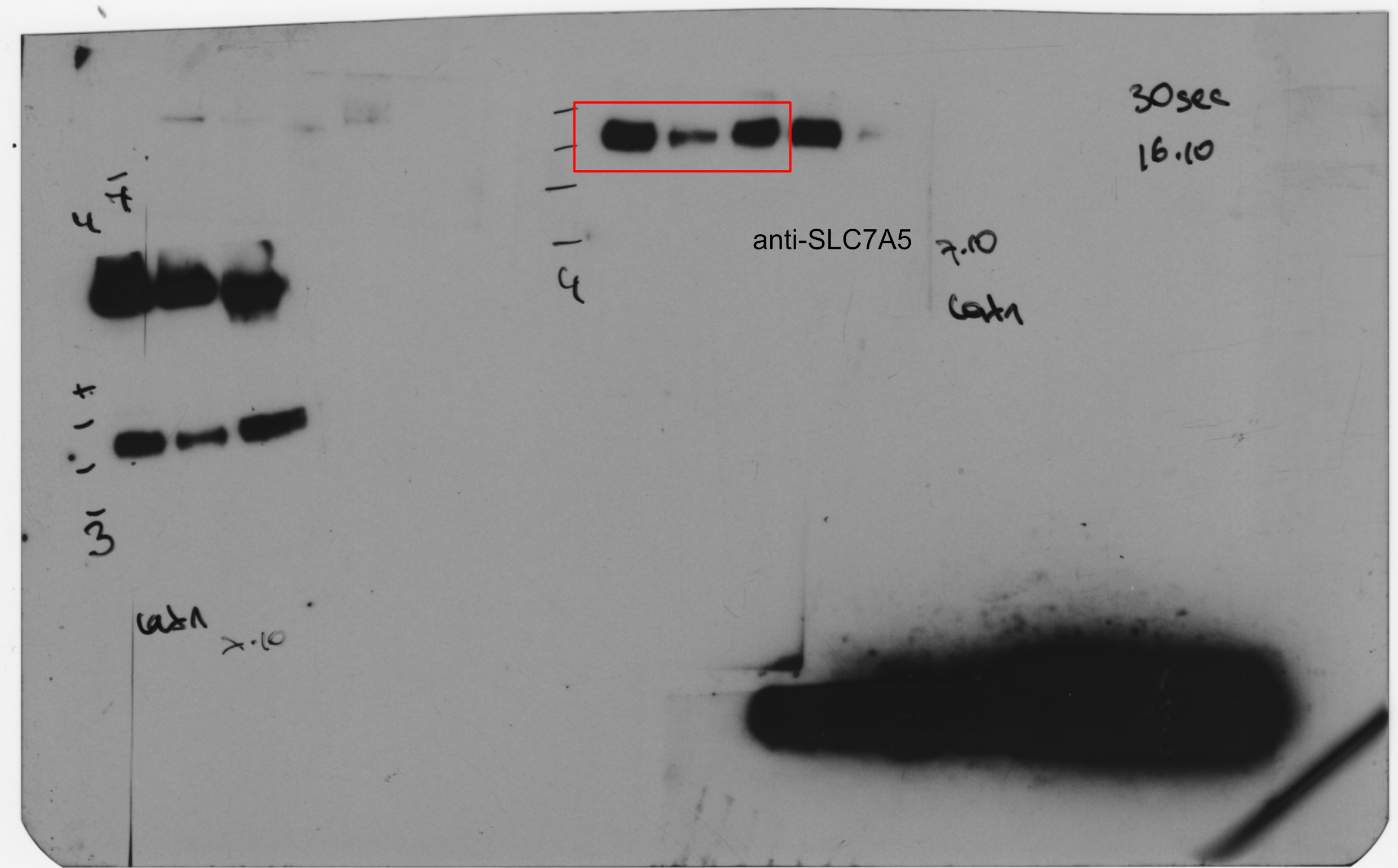

Supplement: Supplementary file 4 — Source data Fig. 1 [file 44318_2025_608_MOESM4_ESM.zip › Figure 1/1C/western SLC7A5.tiff]

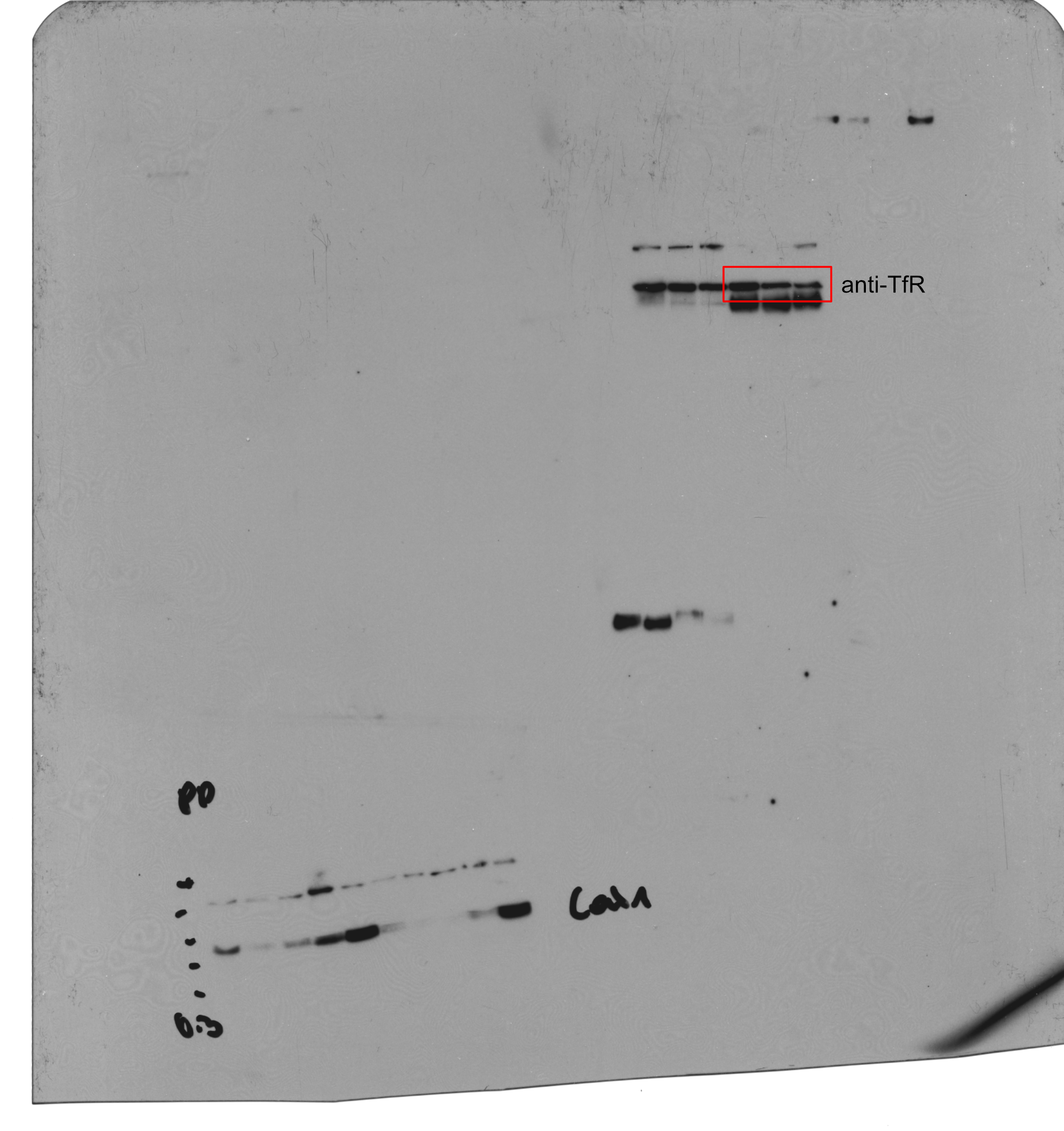

Supplement: Supplementary file 4 — Source data Fig. 1 [file 44318_2025_608_MOESM4_ESM.zip › Figure 1/1C/western TfR.tiff]

Figure 1F

CQ - - + +  
serum + - + -

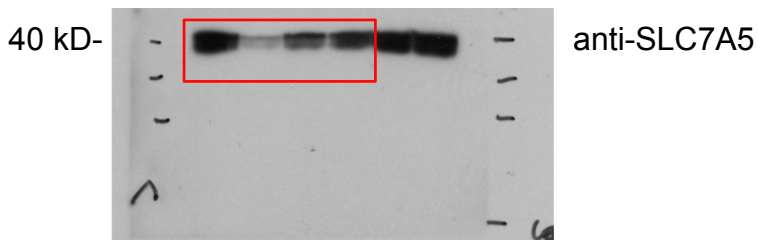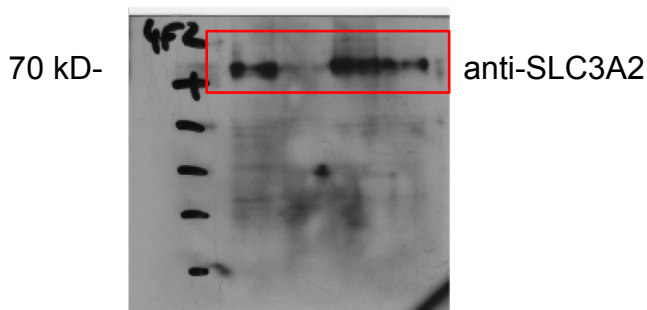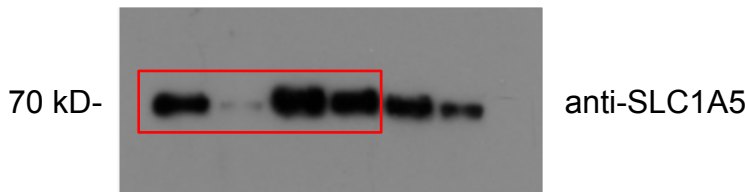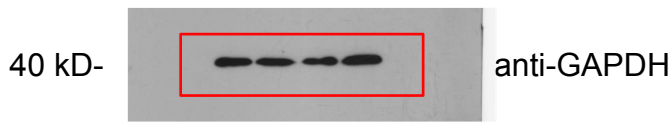

Supplement: Supplementary file 4 — Source data Fig. 1 [file 44318_2025_608_MOESM4_ESM.zip › Figure 1/1F/Figure 1F.pdf]

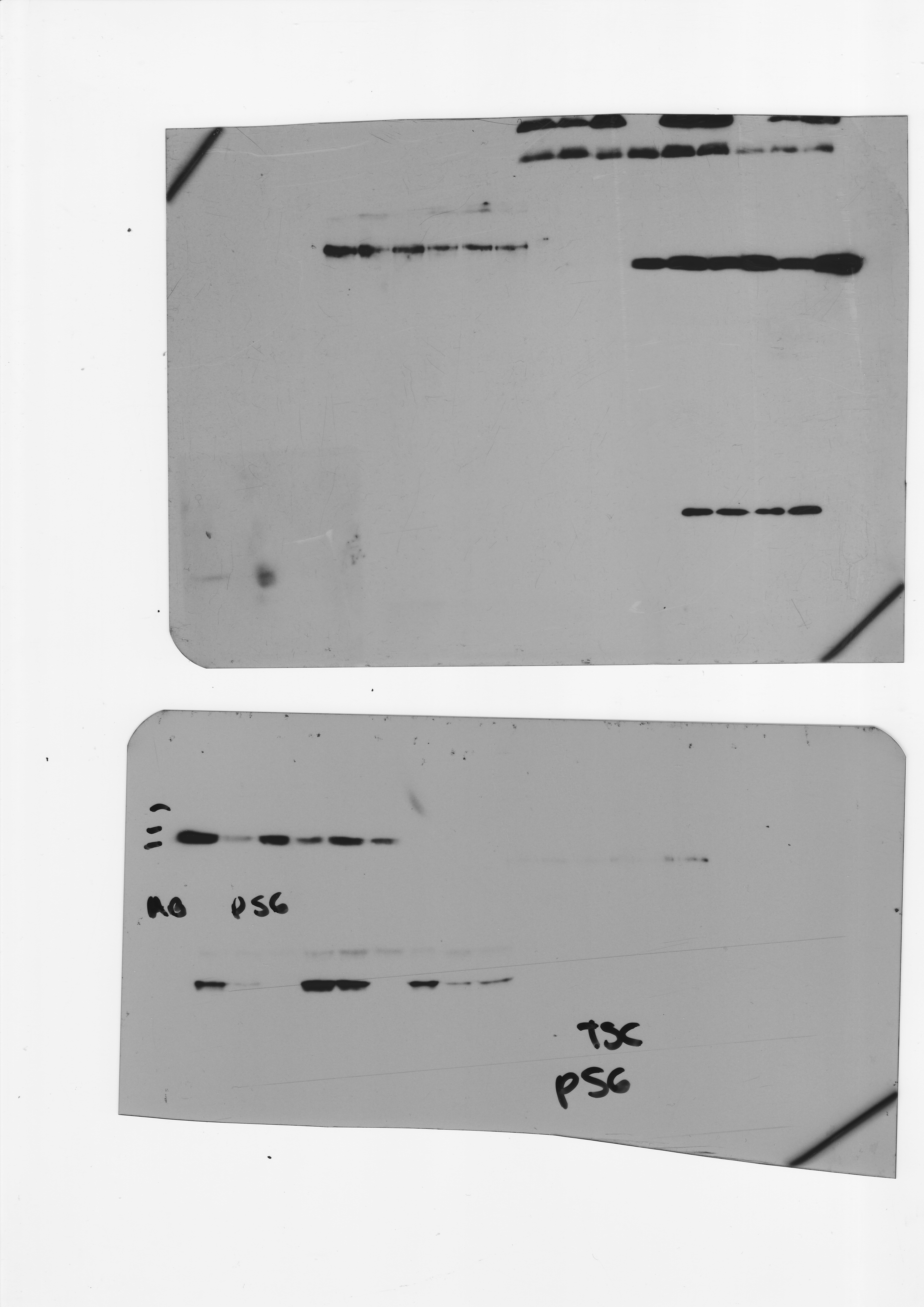

Supplement: Supplementary file 4 — Source data Fig. 1 [file 44318_2025_608_MOESM4_ESM.zip › Figure 1/1F/western GAPDH.TIF]

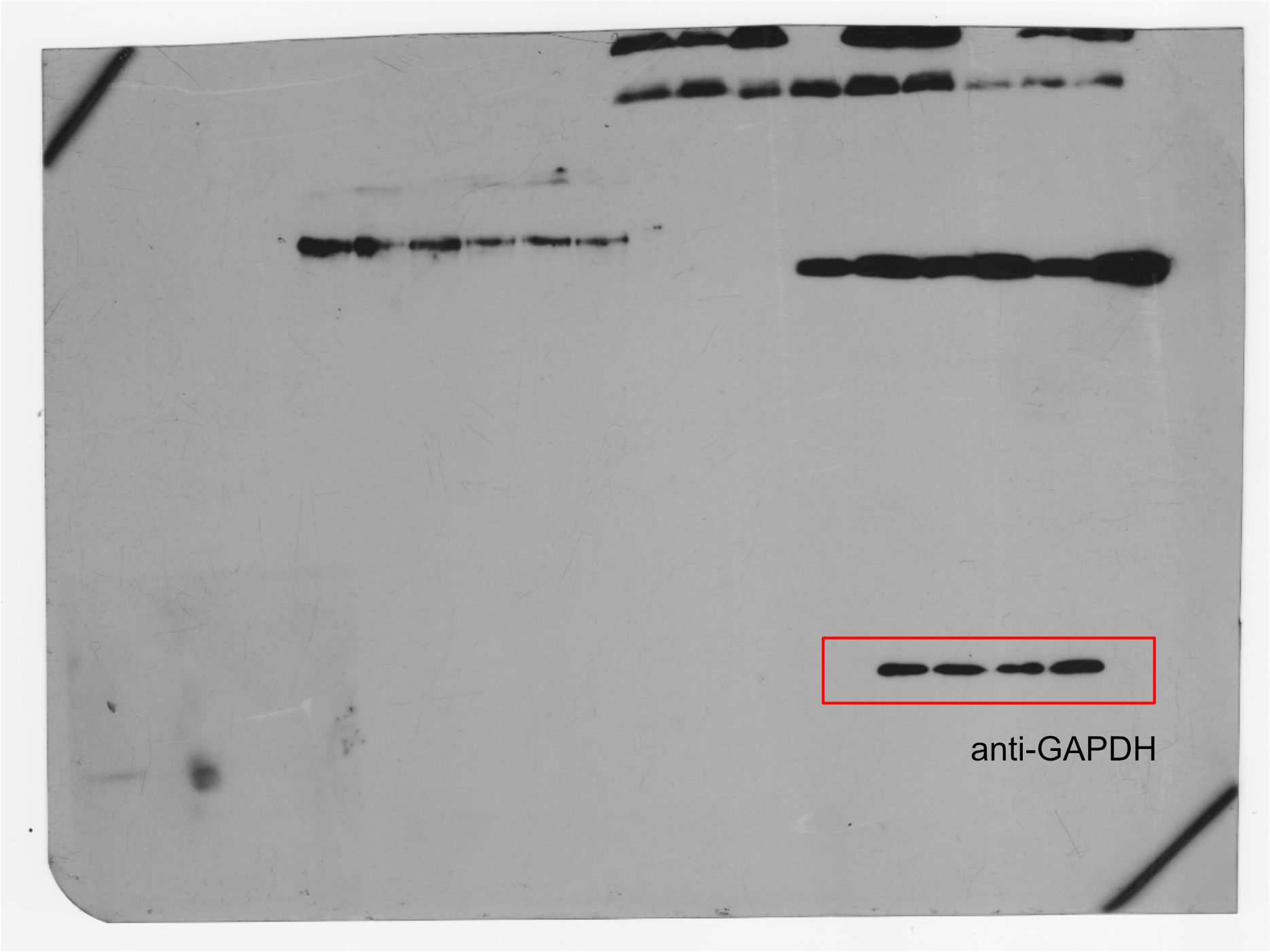

Supplement: Supplementary file 4 — Source data Fig. 1 [file 44318_2025_608_MOESM4_ESM.zip › Figure 1/1F/western GAPDH.tiff]

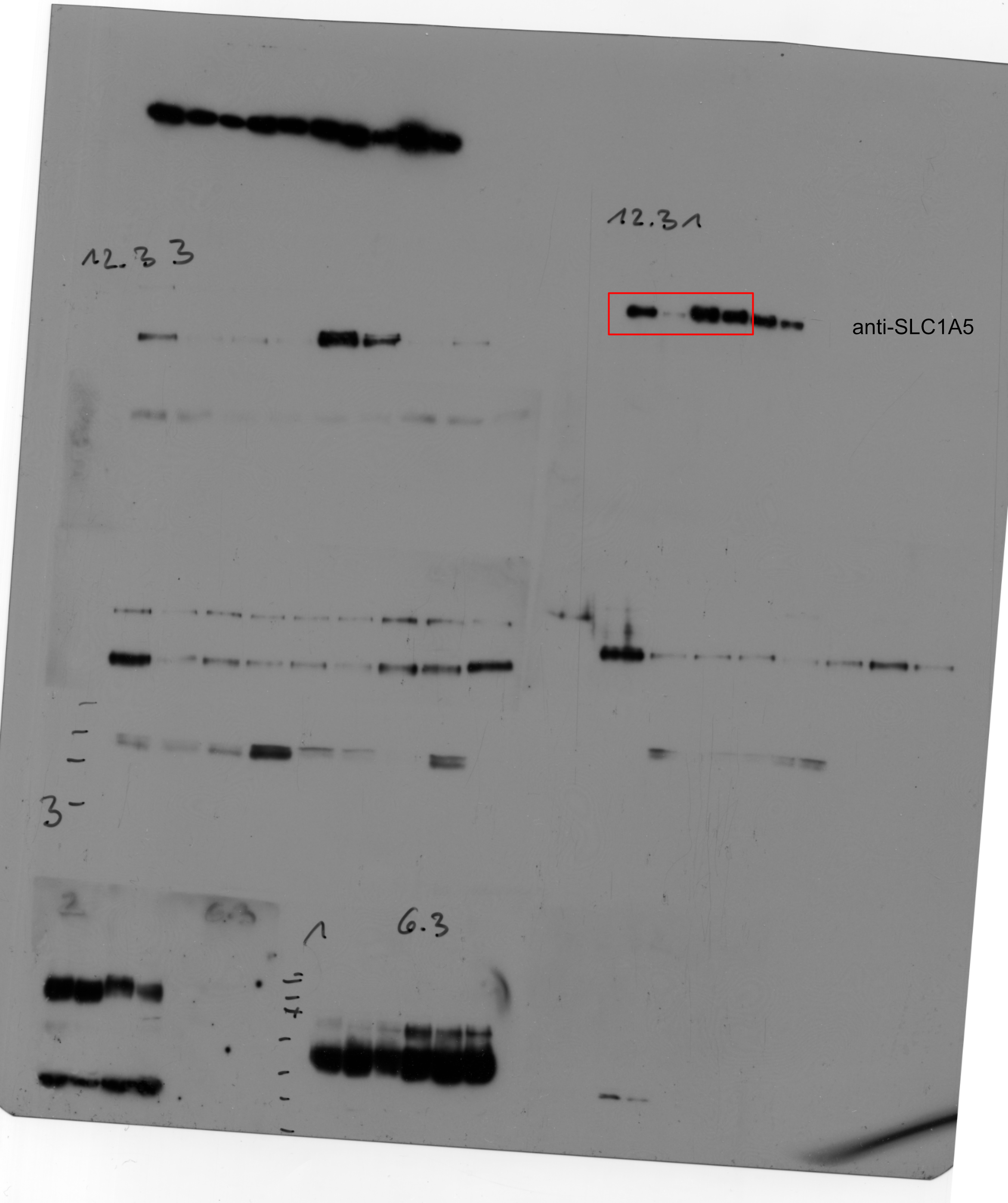

Supplement: Supplementary file 4 — Source data Fig. 1 [file 44318_2025_608_MOESM4_ESM.zip › Figure 1/1F/western SLC1A5.tiff]

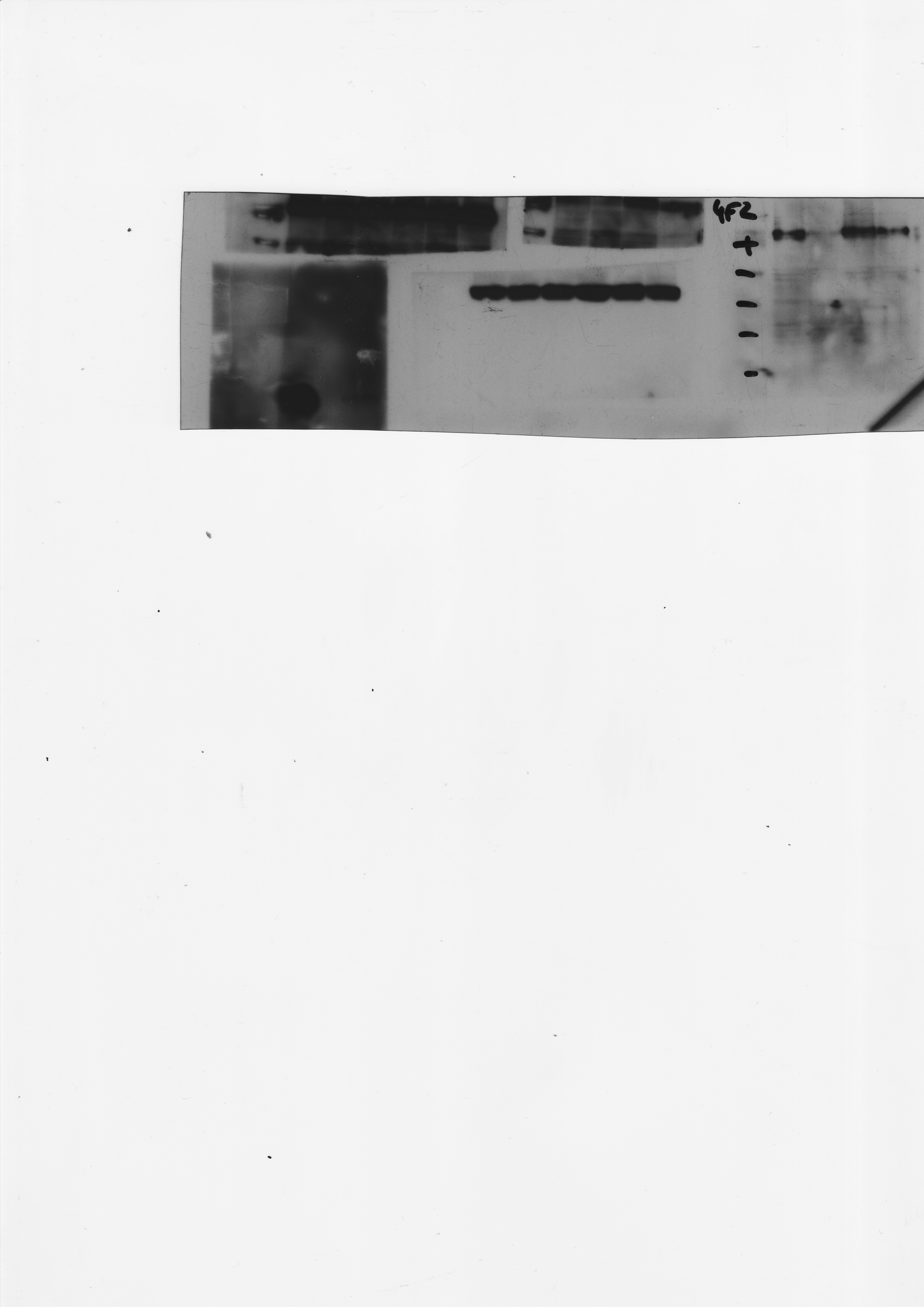

Supplement: Supplementary file 4 — Source data Fig. 1 [file 44318_2025_608_MOESM4_ESM.zip › Figure 1/1F/western SLC3A2.TIF]

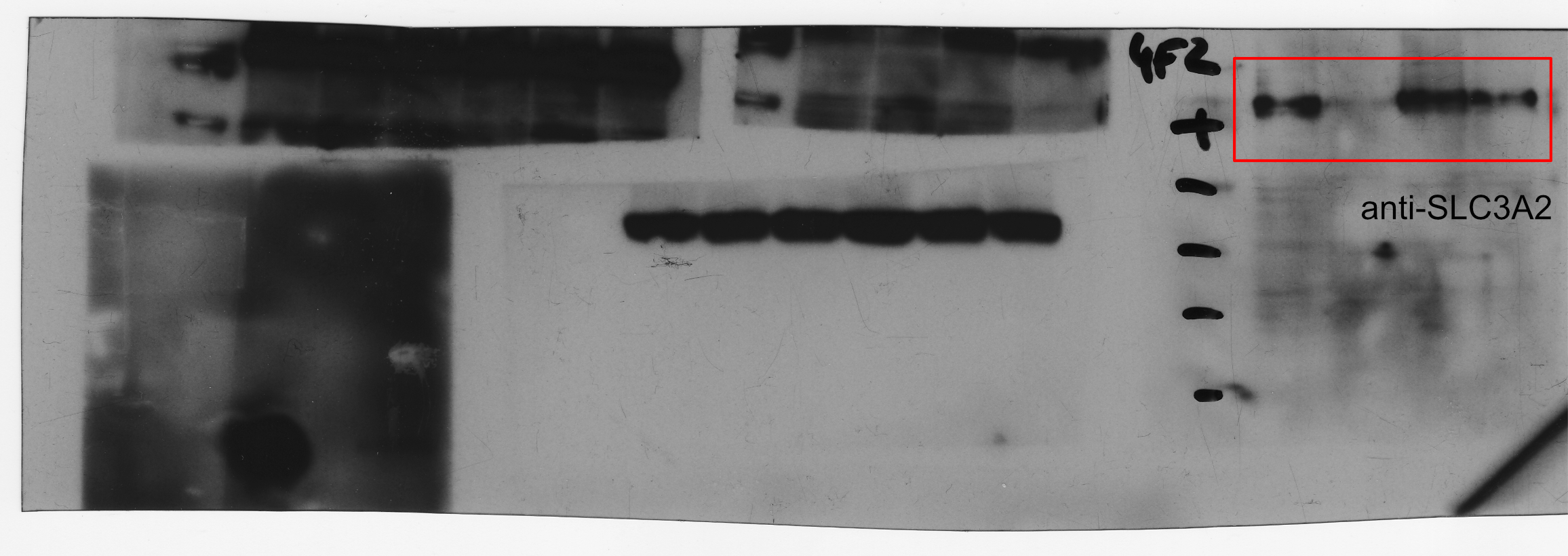

Supplement: Supplementary file 4 — Source data Fig. 1 [file 44318_2025_608_MOESM4_ESM.zip › Figure 1/1F/western SLC3A2.tiff]

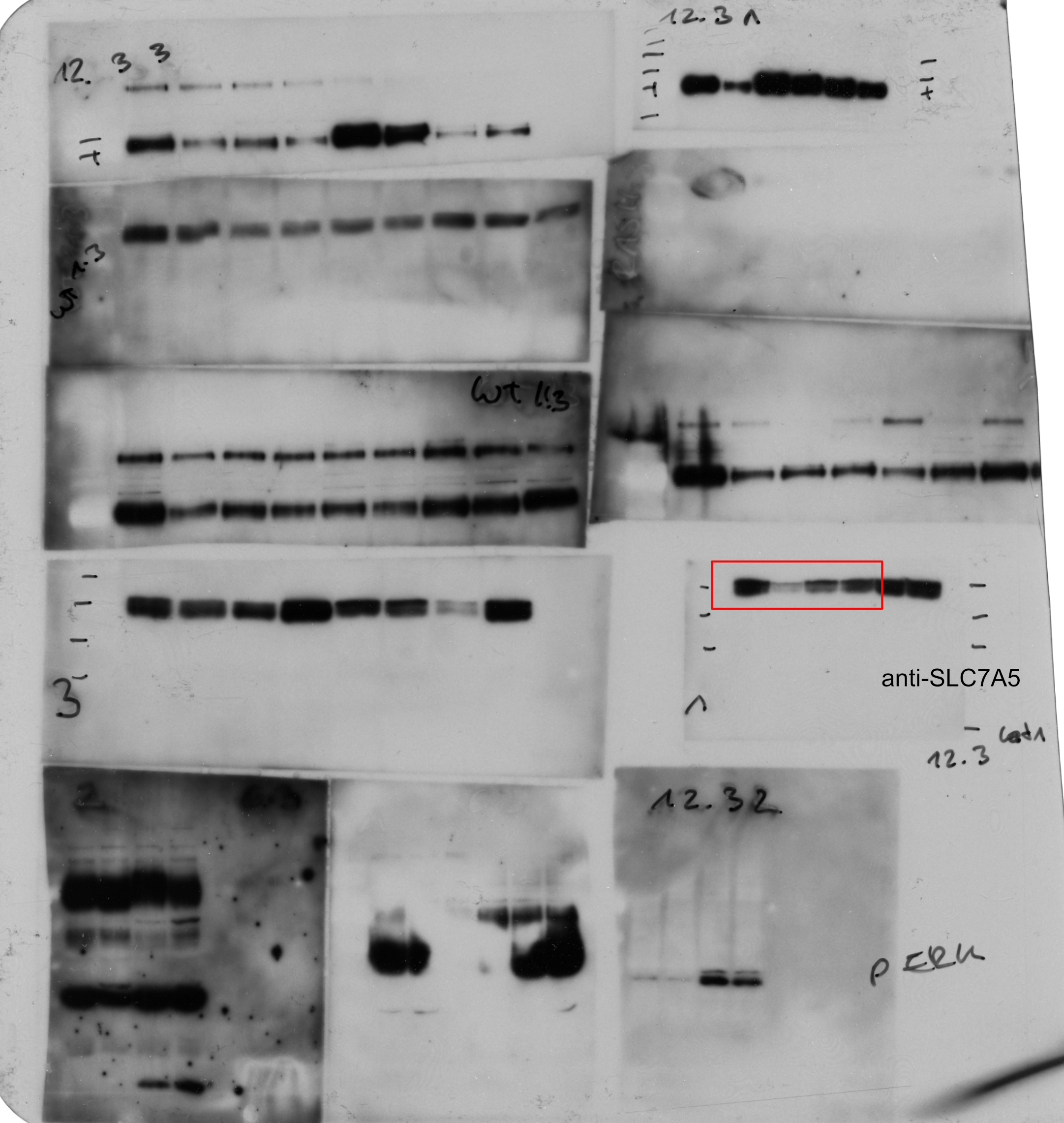

Supplement: Supplementary file 4 — Source data Fig. 1 [file 44318_2025_608_MOESM4_ESM.zip › Figure 1/1F/western SLC7A5.tiff]

Figure 1H

|       |   |   |   |
|-------|---|---|---|
| dyna  | - | - | + |
| serum | + | - | - |

40 kD-

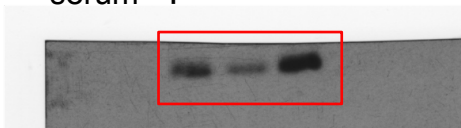

anti-SLC7A5

70 kD-

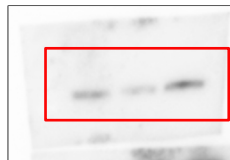

anti-SLC3A2

40 kD-

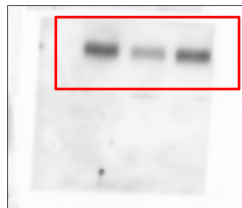

anti-SLC7A11

70 kD-

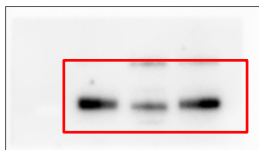

anti-SLC1A5

40 kD-

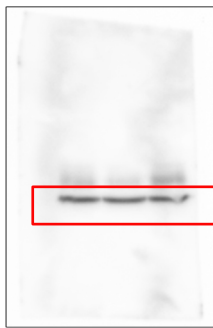

anti-GAPDH

Supplement: Supplementary file 4 — Source data Fig. 1 [file 44318_2025_608_MOESM4_ESM.zip › Figure 1/1H/Figure 1H.pdf]

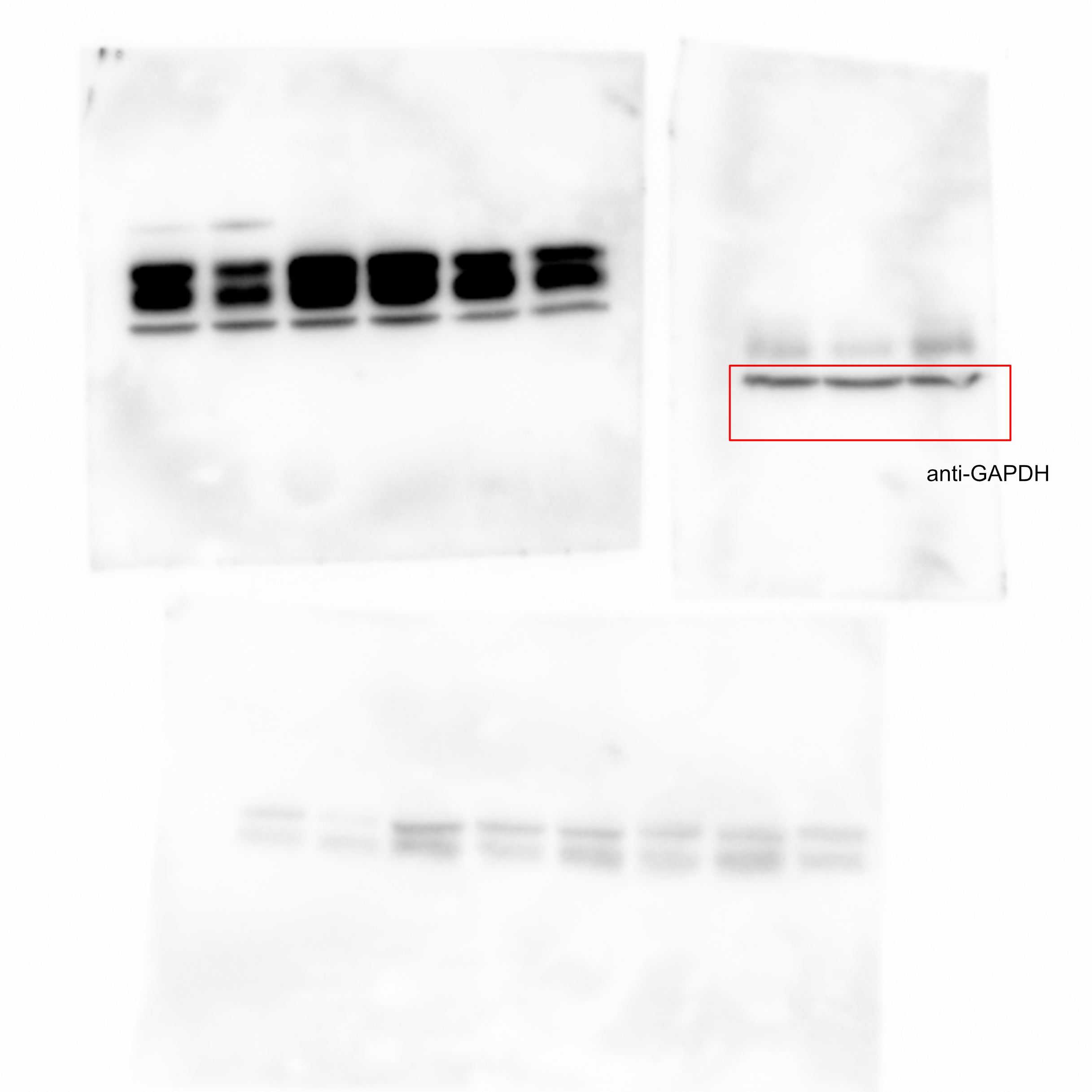

Supplement: Supplementary file 4 — Source data Fig. 1 [file 44318_2025_608_MOESM4_ESM.zip › Figure 1/1H/western GAPDH.tiff]

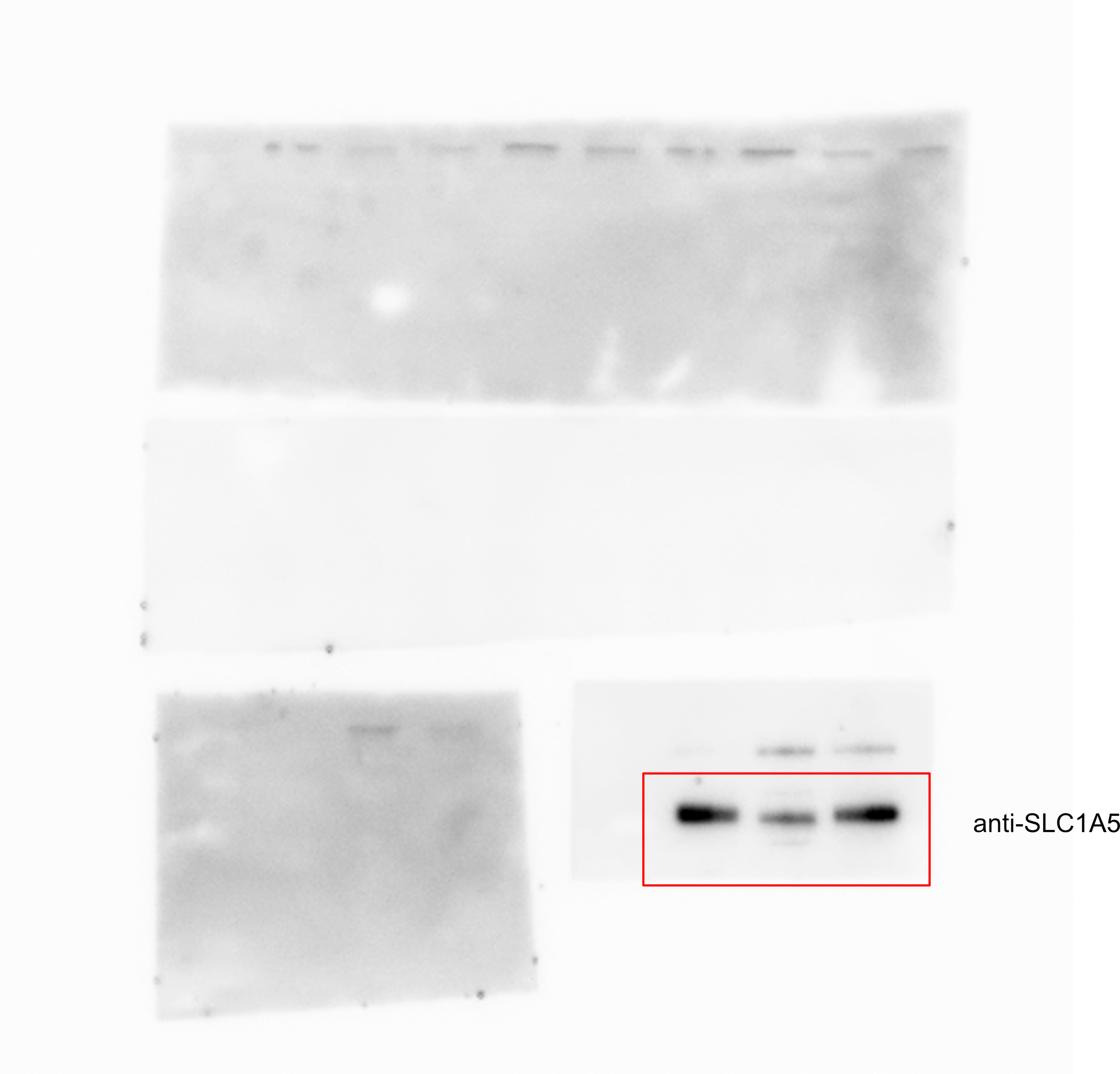

Supplement: Supplementary file 4 — Source data Fig. 1 [file 44318_2025_608_MOESM4_ESM.zip › Figure 1/1H/western SLC1A5.tiff]

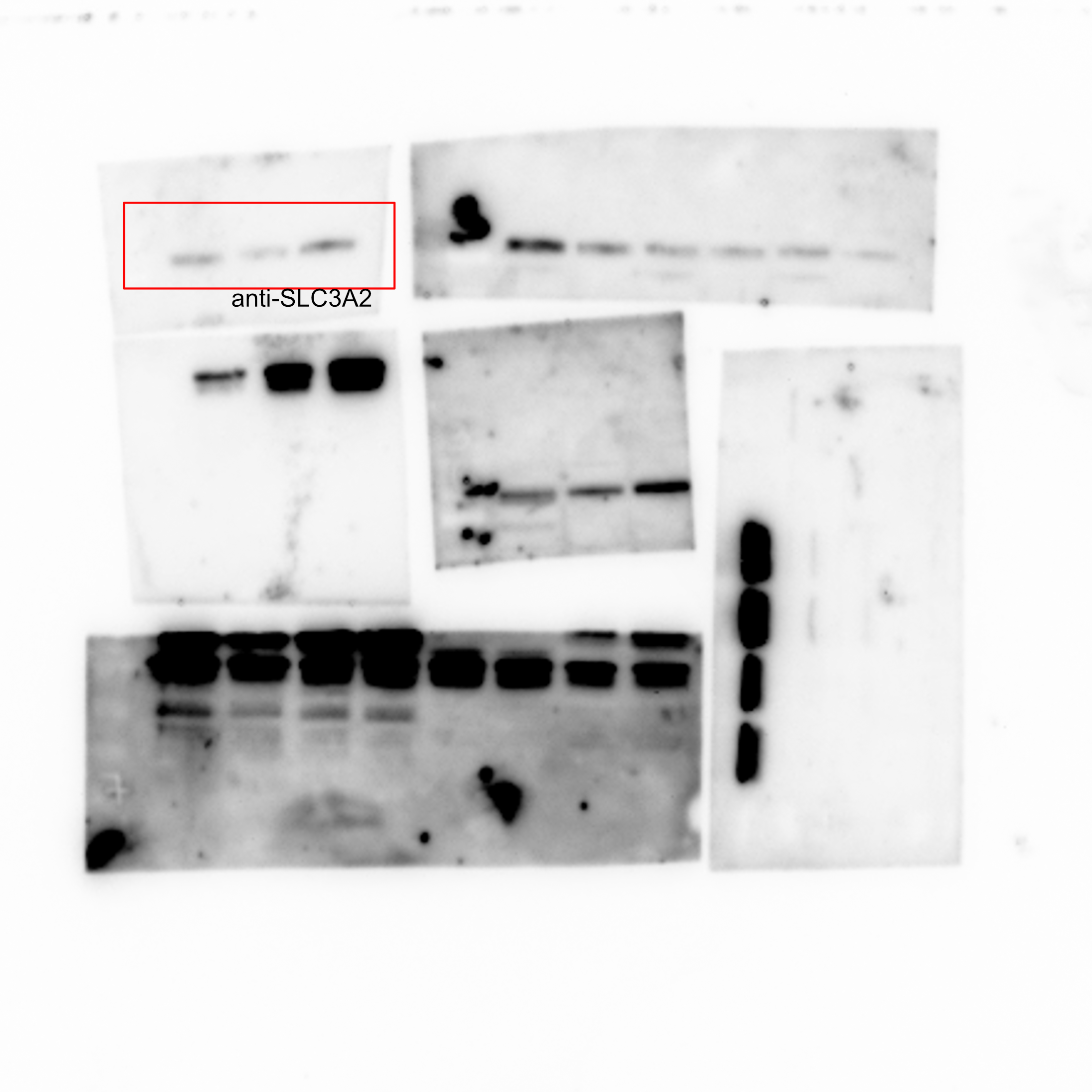

Supplement: Supplementary file 4 — Source data Fig. 1 [file 44318_2025_608_MOESM4_ESM.zip › Figure 1/1H/western SLC3A2.tiff]

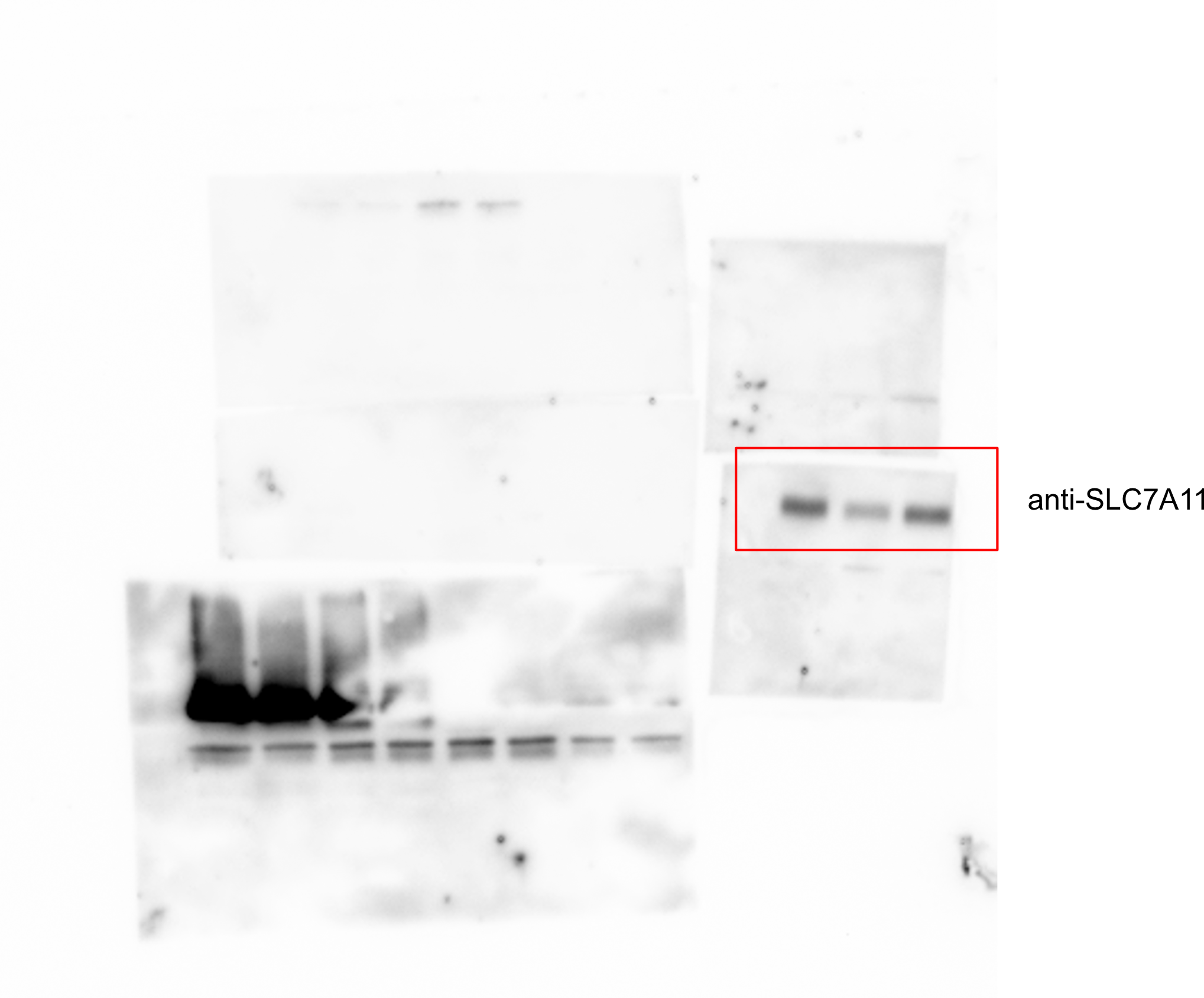

Supplement: Supplementary file 4 — Source data Fig. 1 [file 44318_2025_608_MOESM4_ESM.zip › Figure 1/1H/western SLC7A11.tiff]

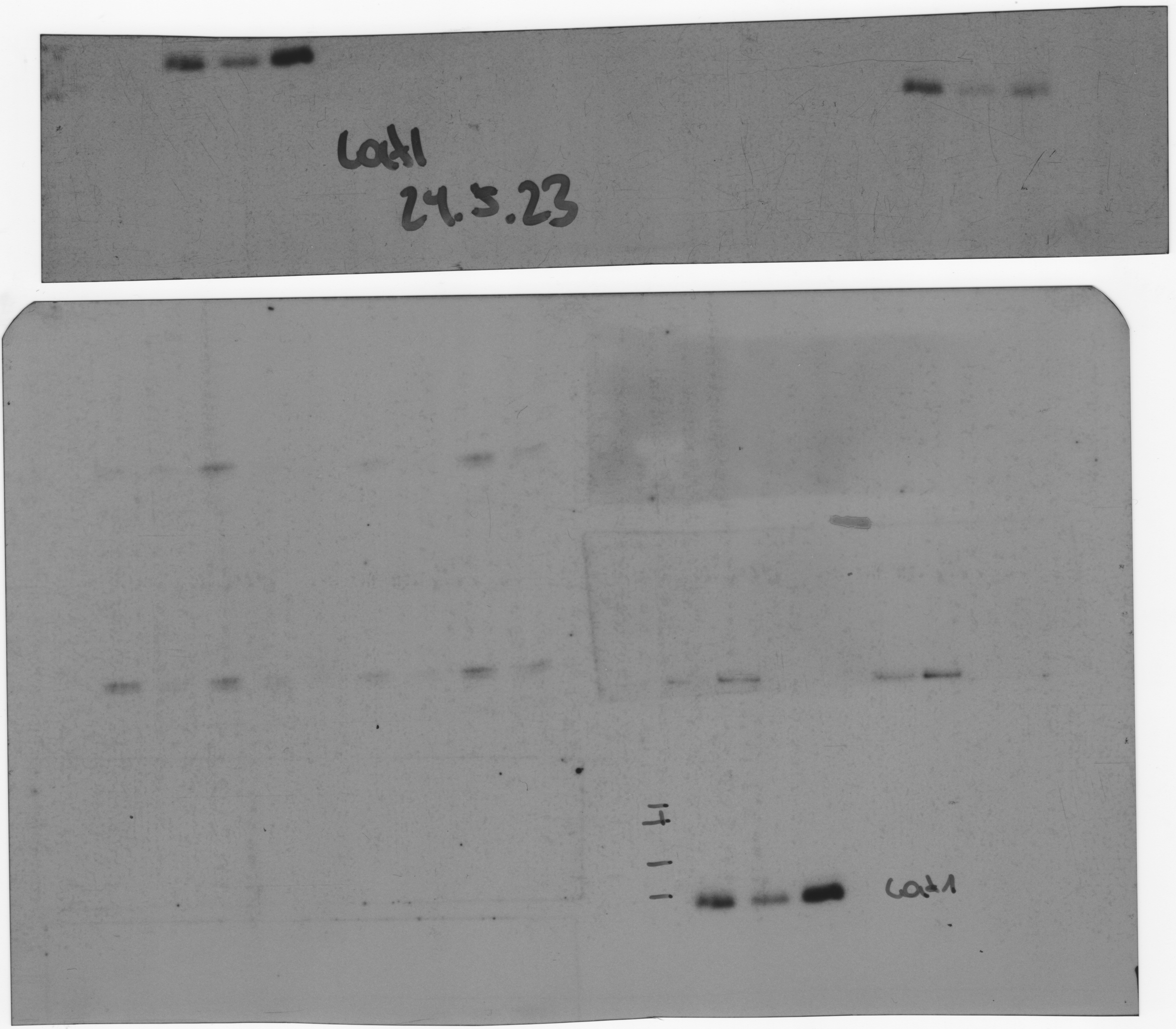

Supplement: Supplementary file 4 — Source data Fig. 1 [file 44318_2025_608_MOESM4_ESM.zip › Figure 1/1H/western SLC7A5.TIF]

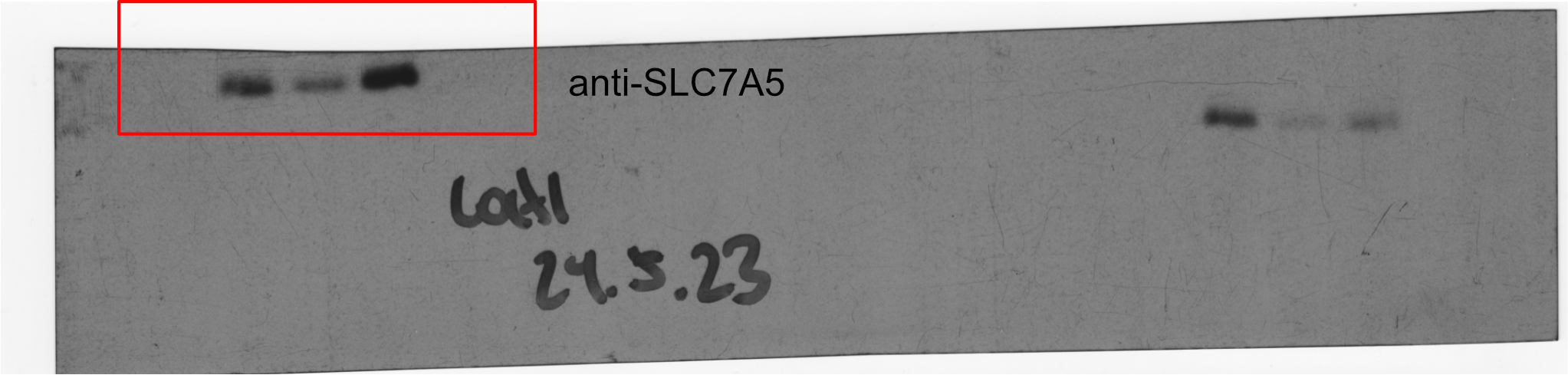

Supplement: Supplementary file 4 — Source data Fig. 1 [file 44318_2025_608_MOESM4_ESM.zip › Figure 1/1H/western SLC7A5.tiff]

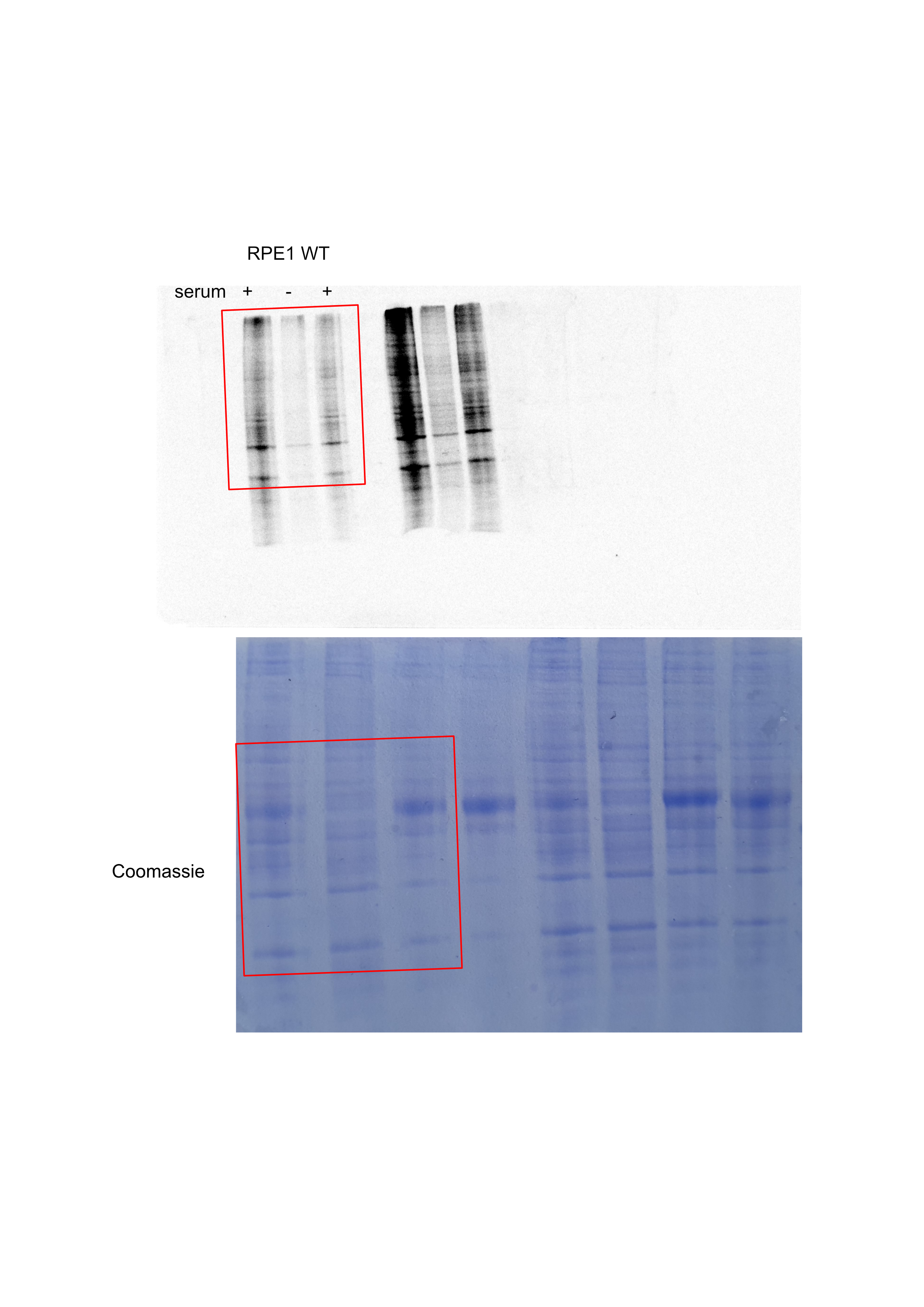

Supplement: Supplementary file 4 — Source data Fig. 1 [file 44318_2025_608_MOESM4_ESM.zip › Figure 1/1J/Figure1J-.tif]

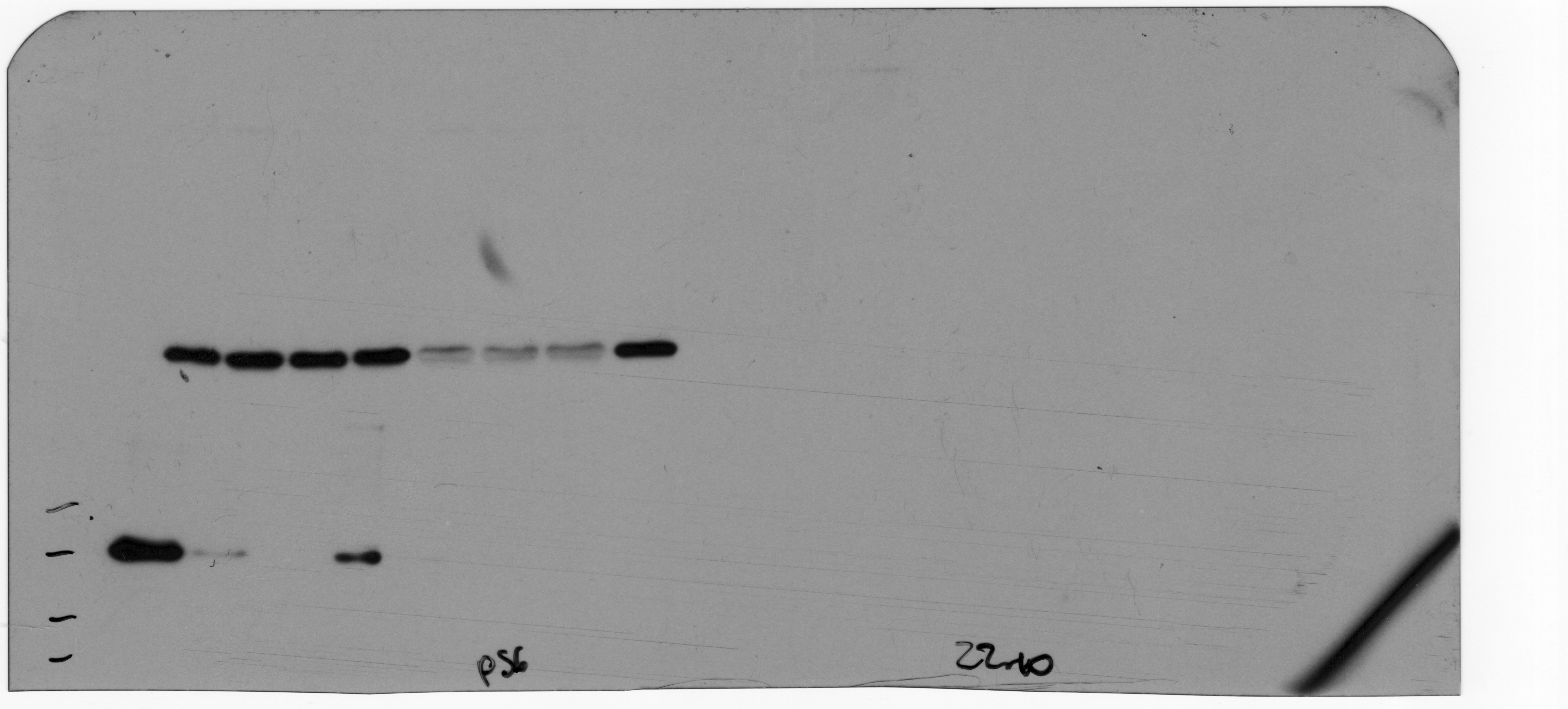

Supplement: Supplementary file 5 — Source data Fig. 2 [file 44318_2025_608_MOESM5_ESM.zip › Figure 2/2A/western GAPDH.TIF]

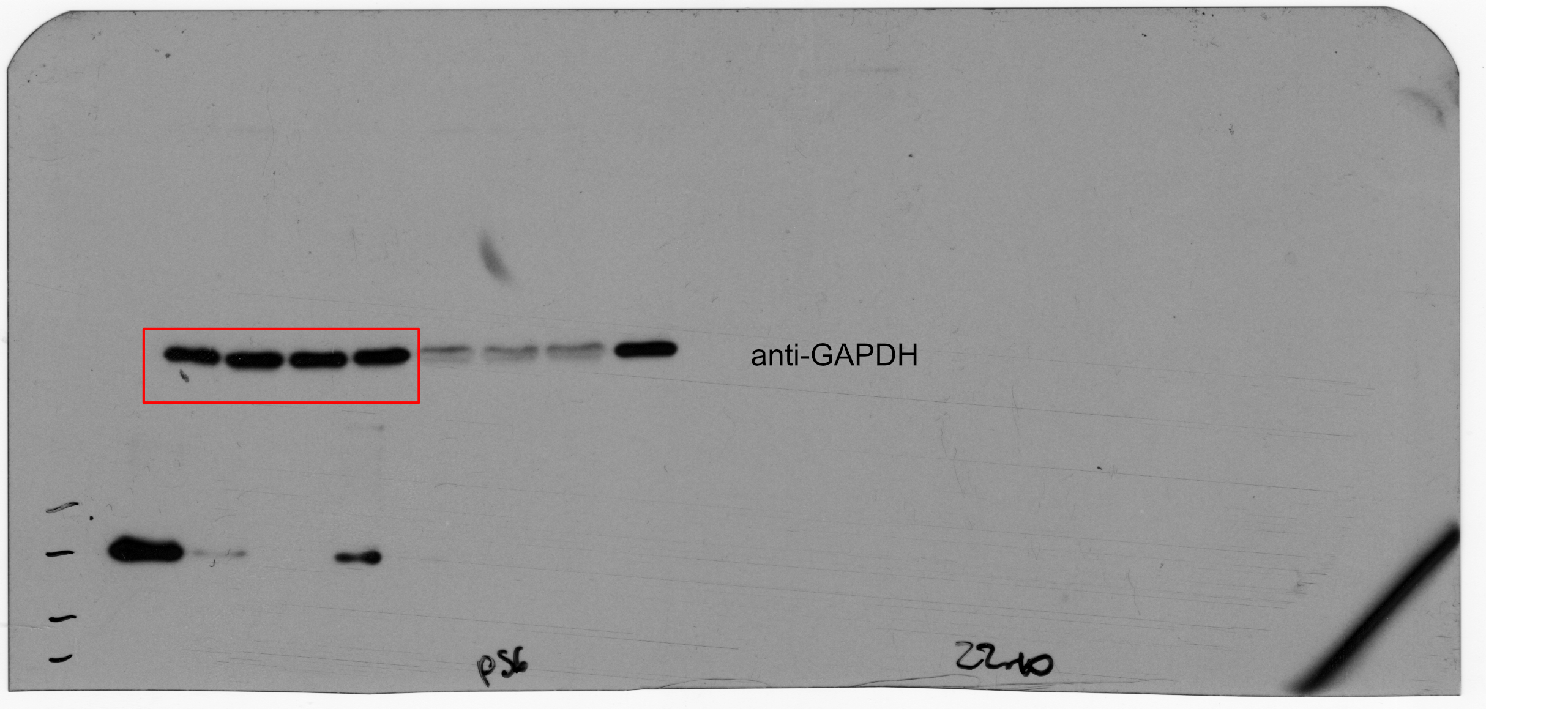

Supplement: Supplementary file 5 — Source data Fig. 2 [file 44318_2025_608_MOESM5_ESM.zip › Figure 2/2A/western GAPDH.tiff]

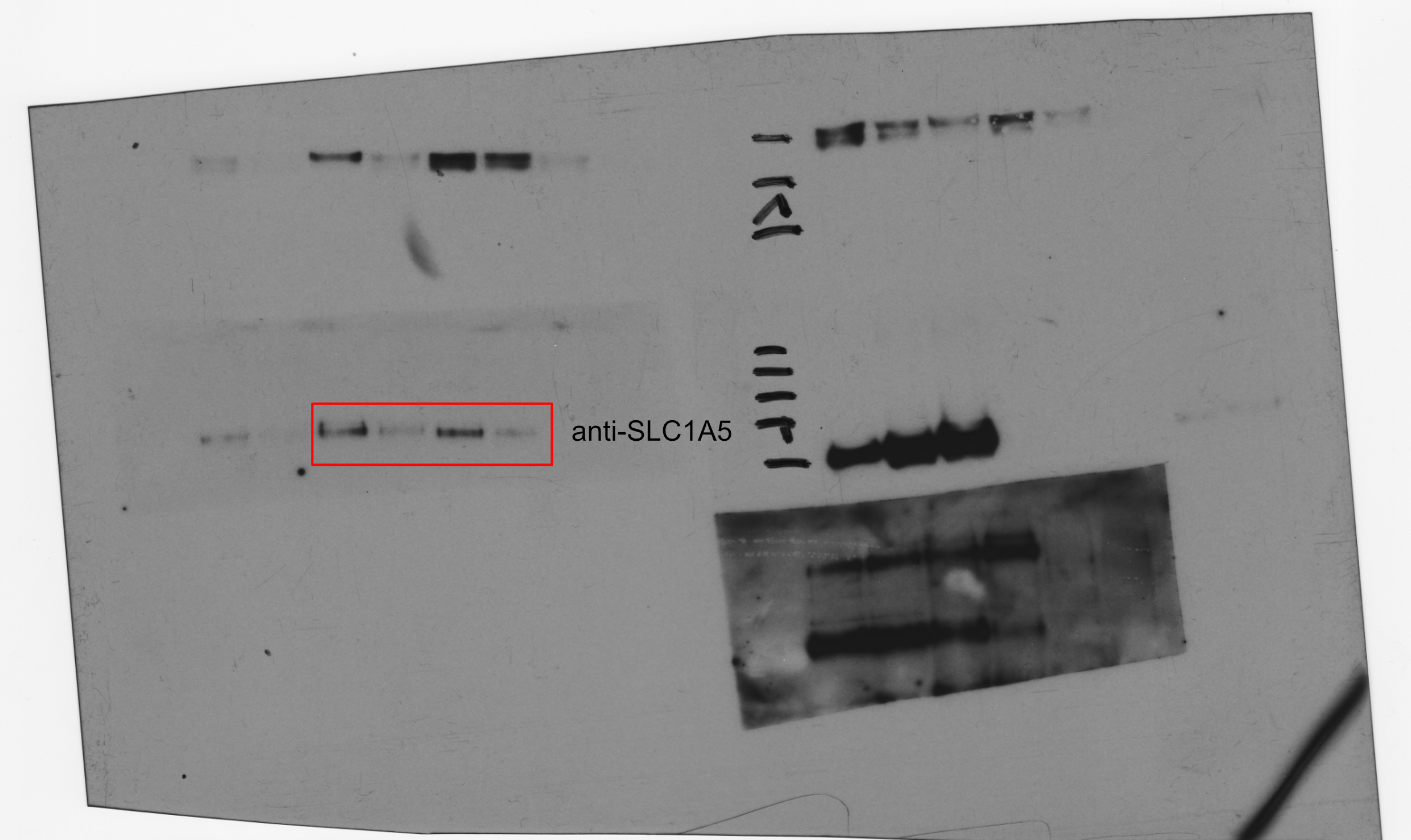

Supplement: Supplementary file 5 — Source data Fig. 2 [file 44318_2025_608_MOESM5_ESM.zip › Figure 2/2A/western SLC1A5.tiff]

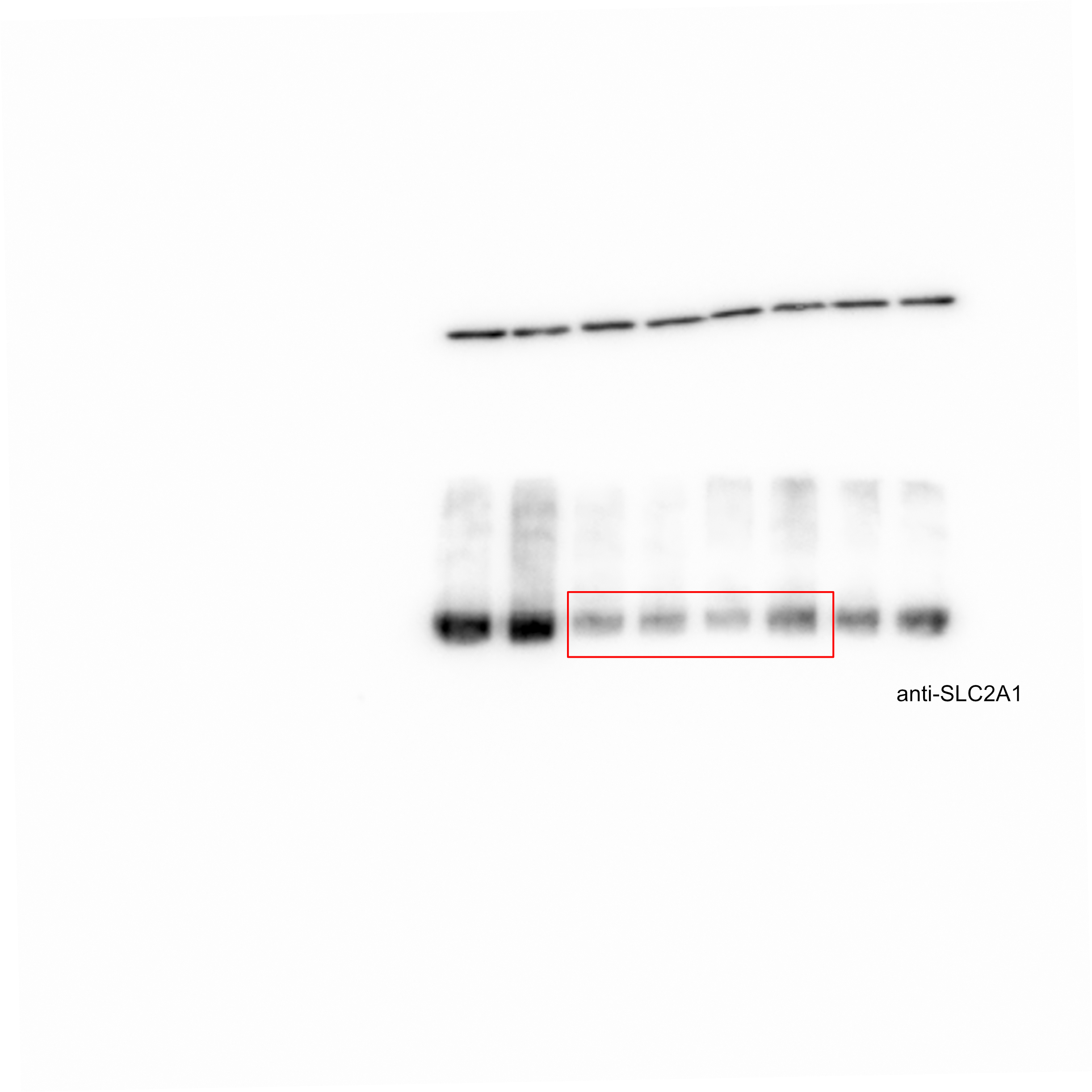

Supplement: Supplementary file 5 — Source data Fig. 2 [file 44318_2025_608_MOESM5_ESM.zip › Figure 2/2A/western SLC2A1.tiff]

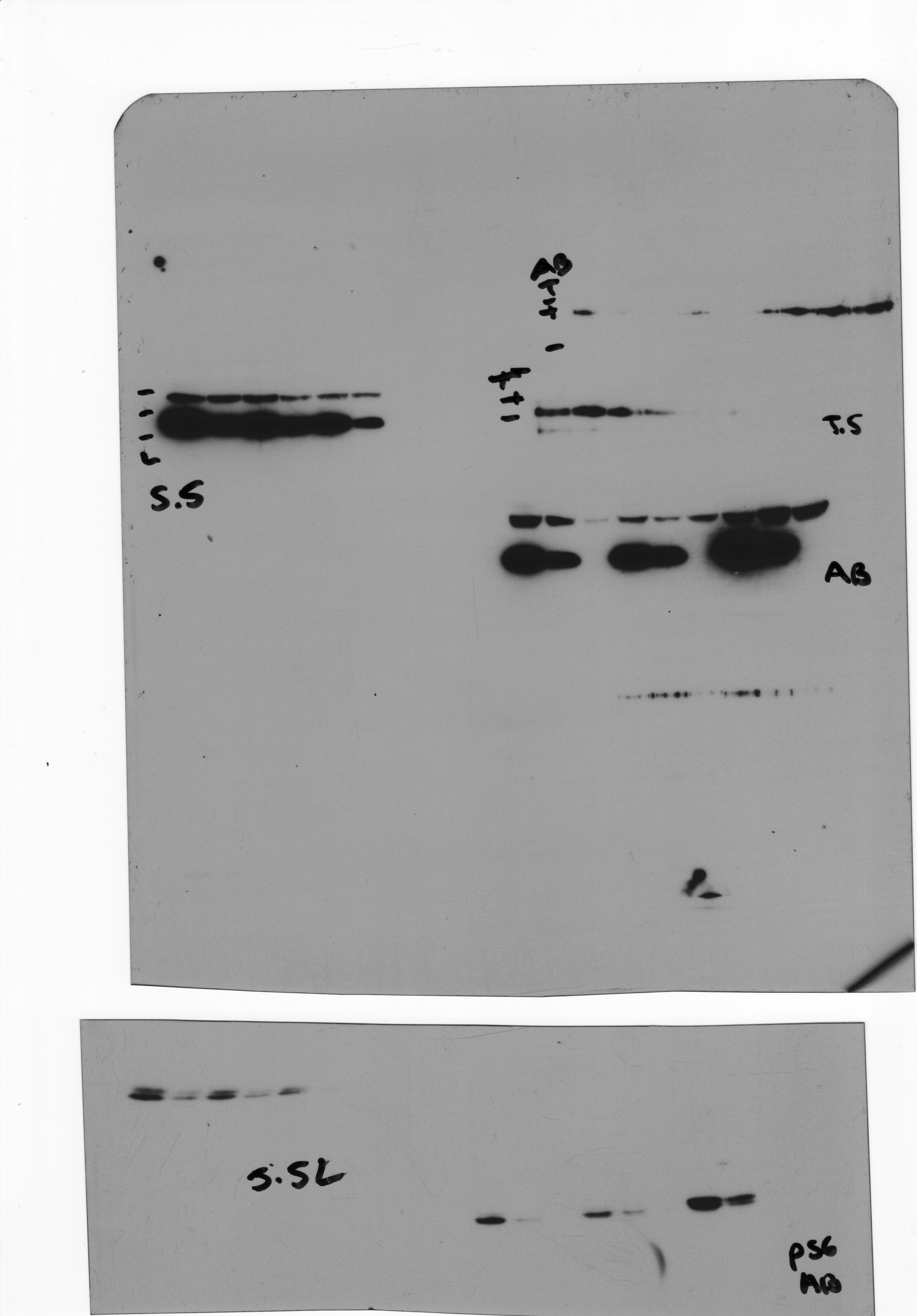

Supplement: Supplementary file 5 — Source data Fig. 2 [file 44318_2025_608_MOESM5_ESM.zip › Figure 2/2A/western SLC3A2.TIF]

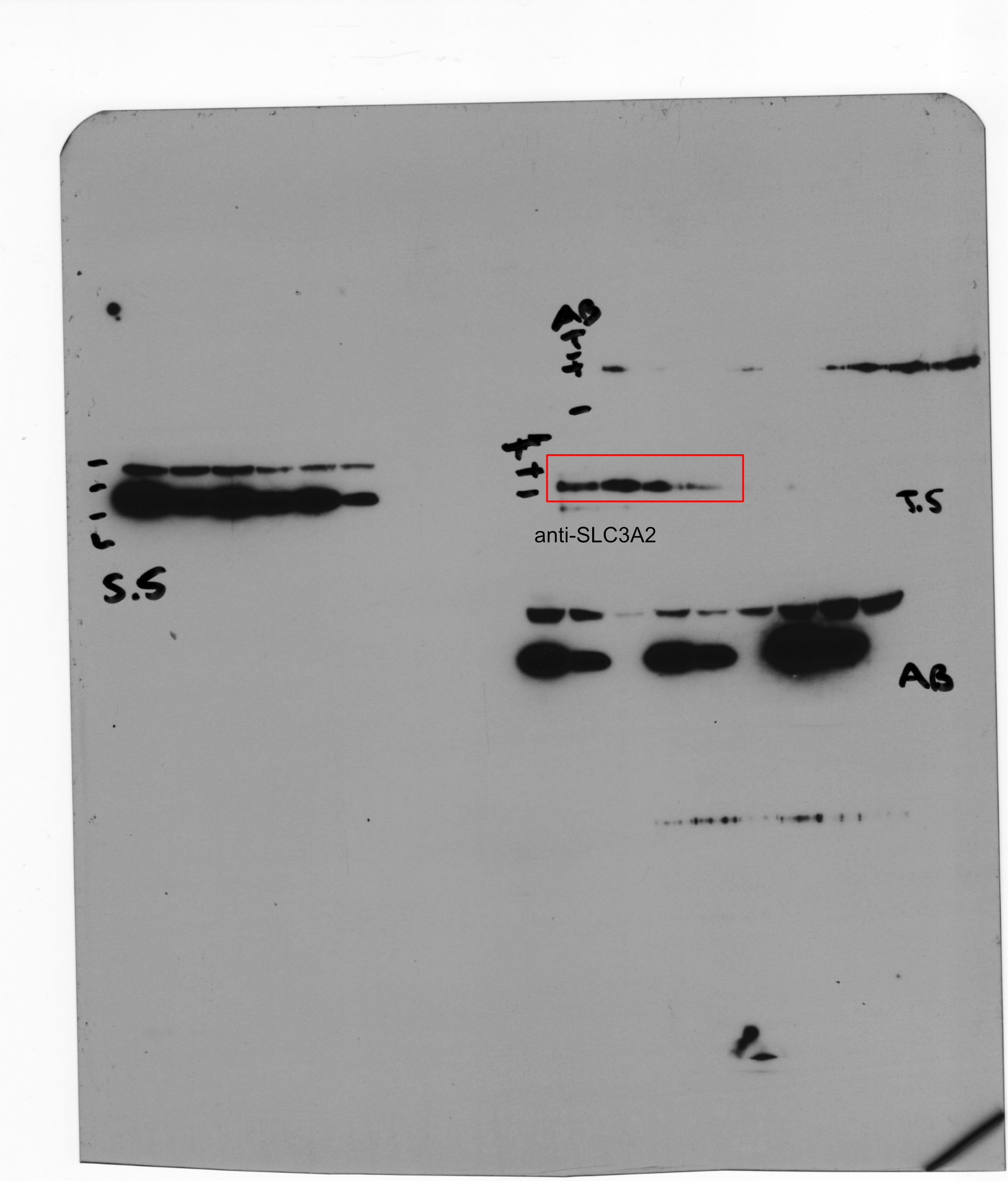

Supplement: Supplementary file 5 — Source data Fig. 2 [file 44318_2025_608_MOESM5_ESM.zip › Figure 2/2A/western SLC3A2.tiff]

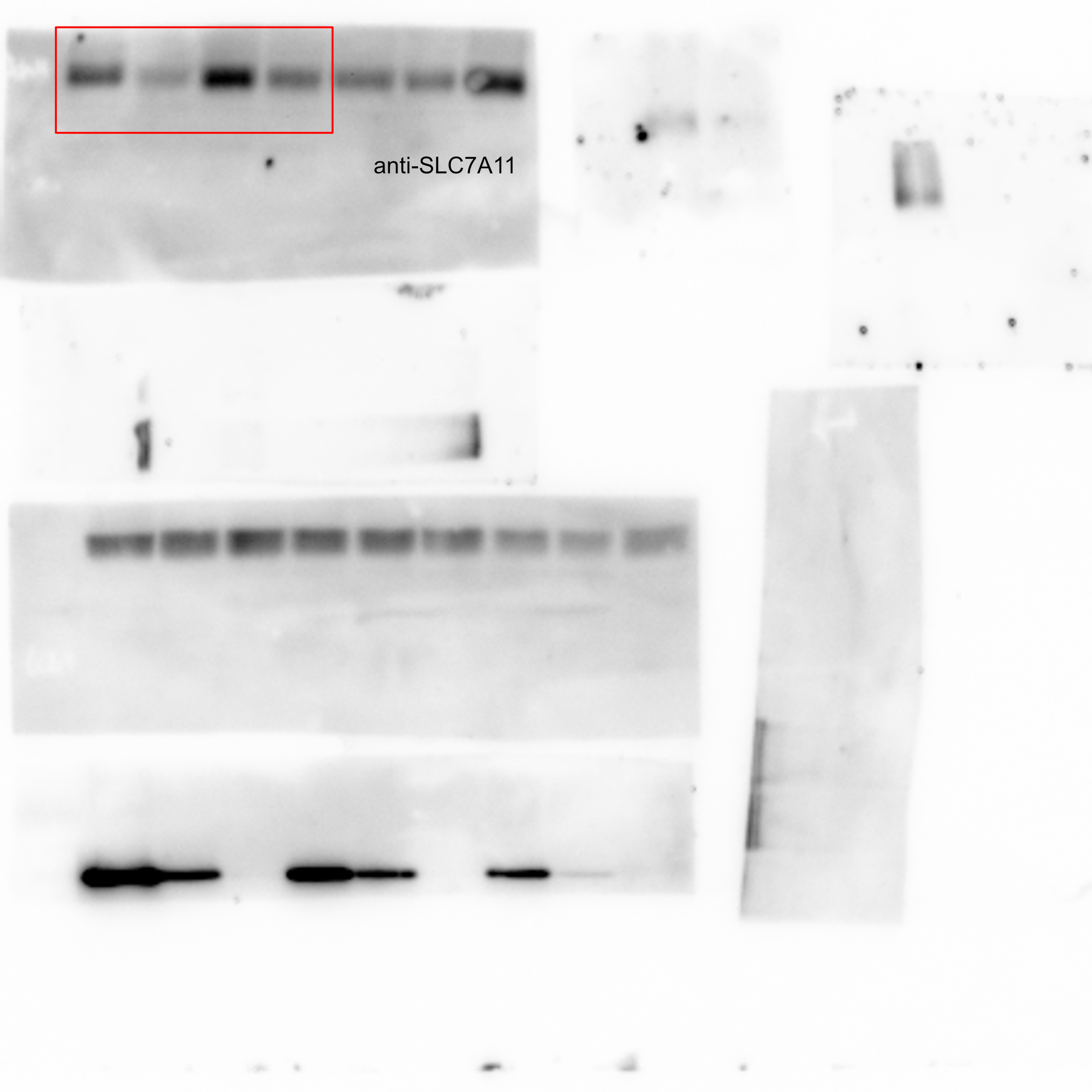

Supplement: Supplementary file 5 — Source data Fig. 2 [file 44318_2025_608_MOESM5_ESM.zip › Figure 2/2A/western SLC7A11.tiff]

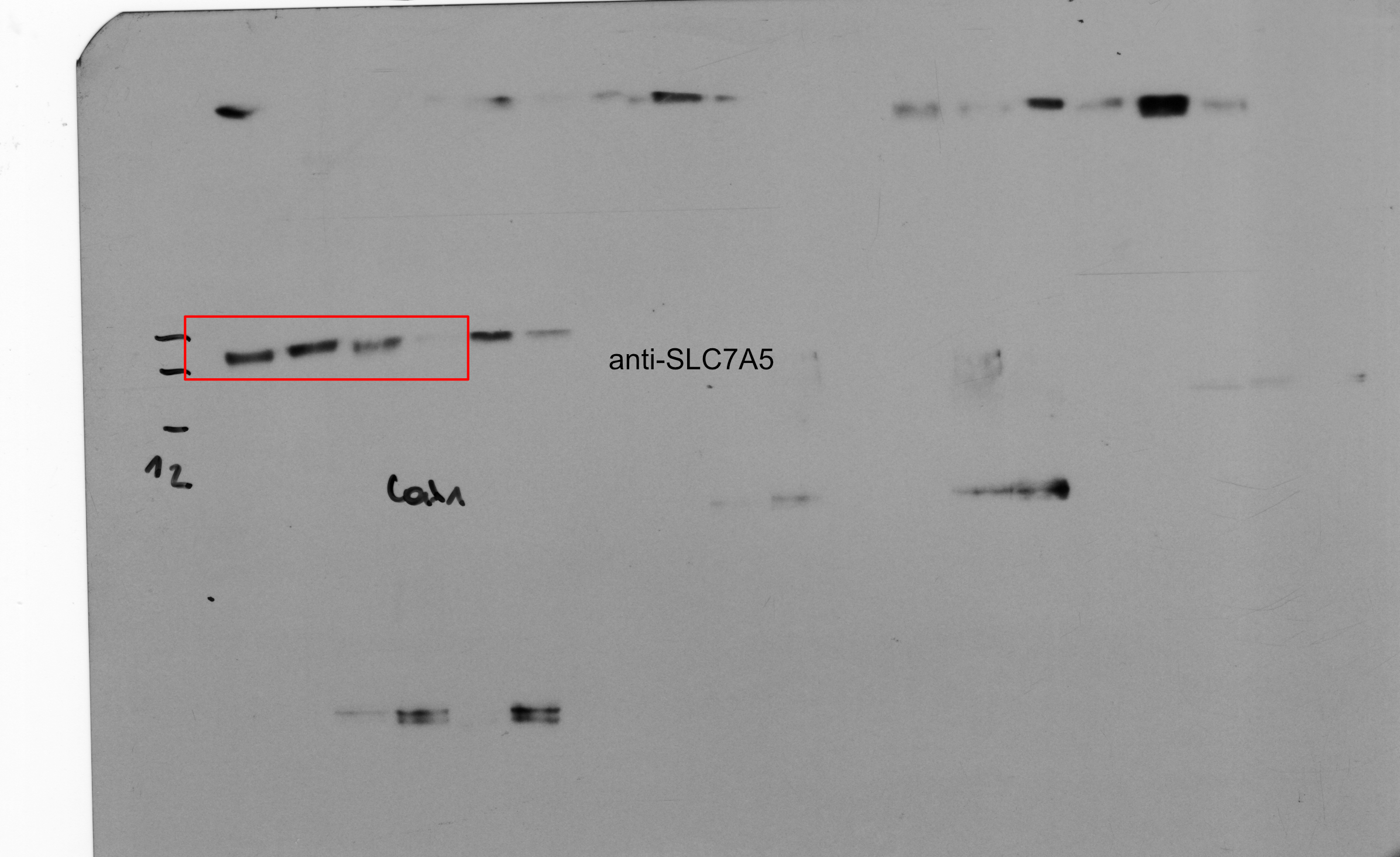

Supplement: Supplementary file 5 — Source data Fig. 2 [file 44318_2025_608_MOESM5_ESM.zip › Figure 2/2A/western SLC7A5.tiff]

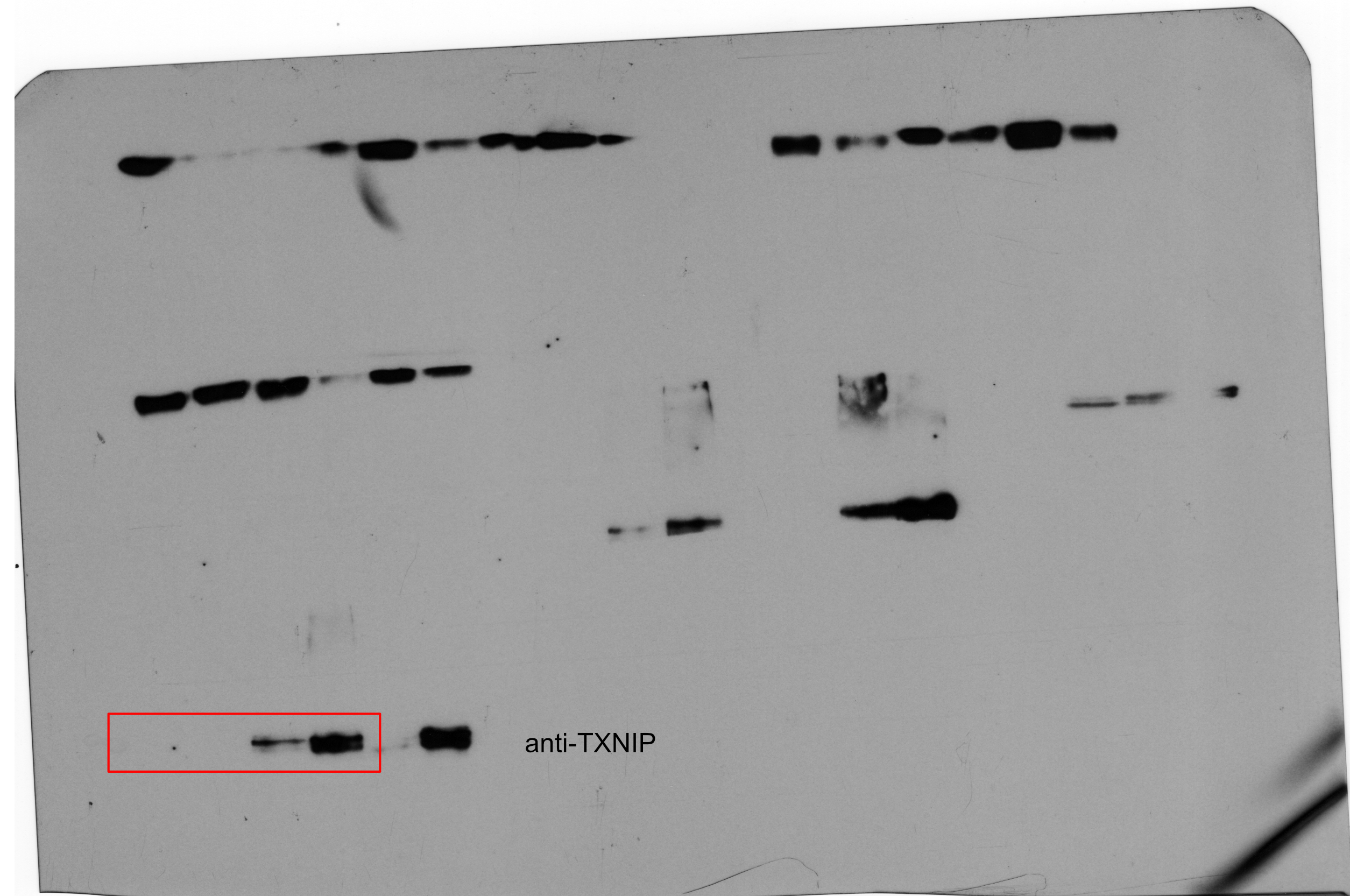

Supplement: Supplementary file 5 — Source data Fig. 2 [file 44318_2025_608_MOESM5_ESM.zip › Figure 2/2A/western TXNIP.tiff]

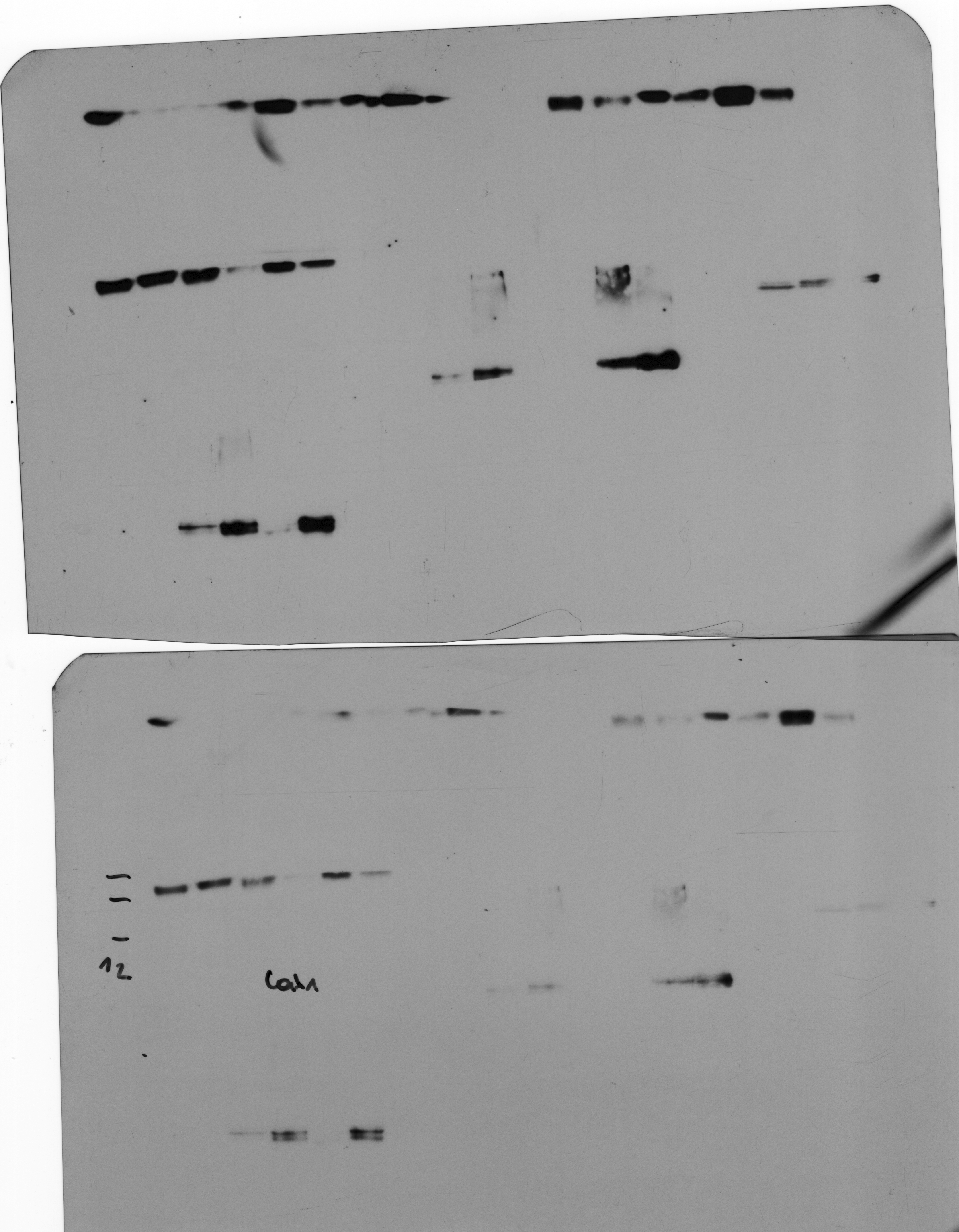

Supplement: Supplementary file 5 — Source data Fig. 2 [file 44318_2025_608_MOESM5_ESM.zip › Figure 2/2A/western TXNIP_SLC7A5.TIF]

Figure 3B

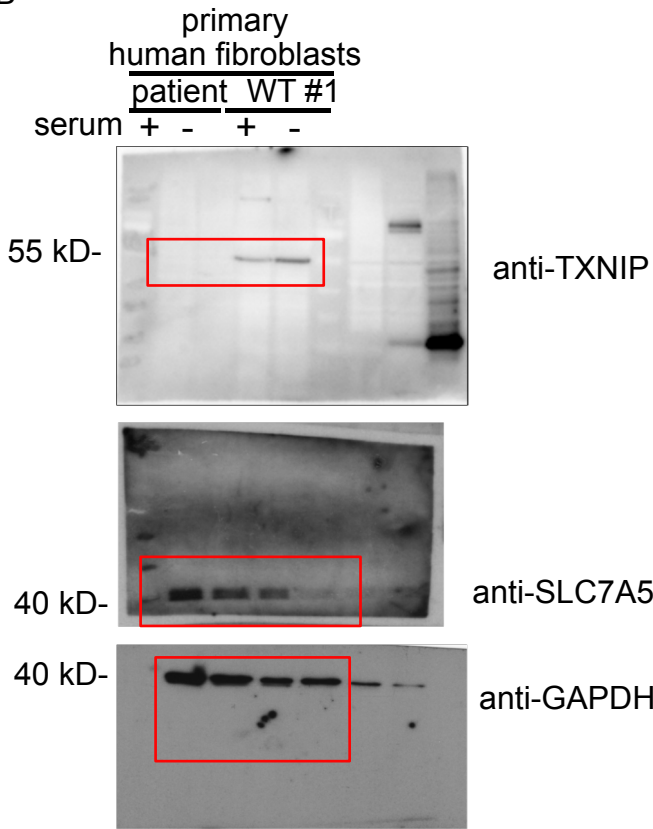

Supplement: Supplementary file 6 — Source data Fig. 3 [file 44318_2025_608_MOESM6_ESM.zip › Figure 3/3B/Figure 3B.pdf]

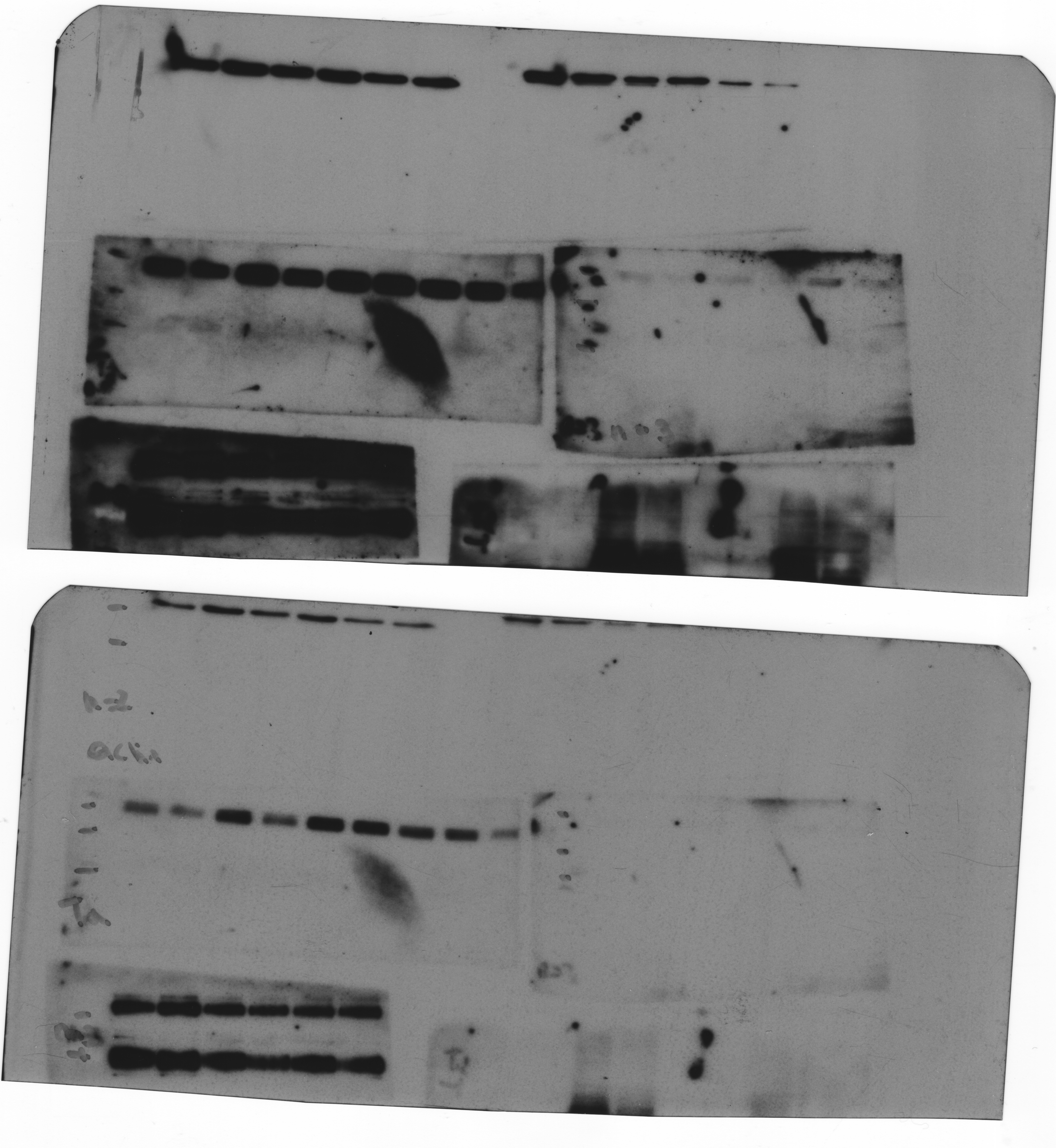

Supplement: Supplementary file 6 — Source data Fig. 3 [file 44318_2025_608_MOESM6_ESM.zip › Figure 3/3B/western GAPDH.TIF]

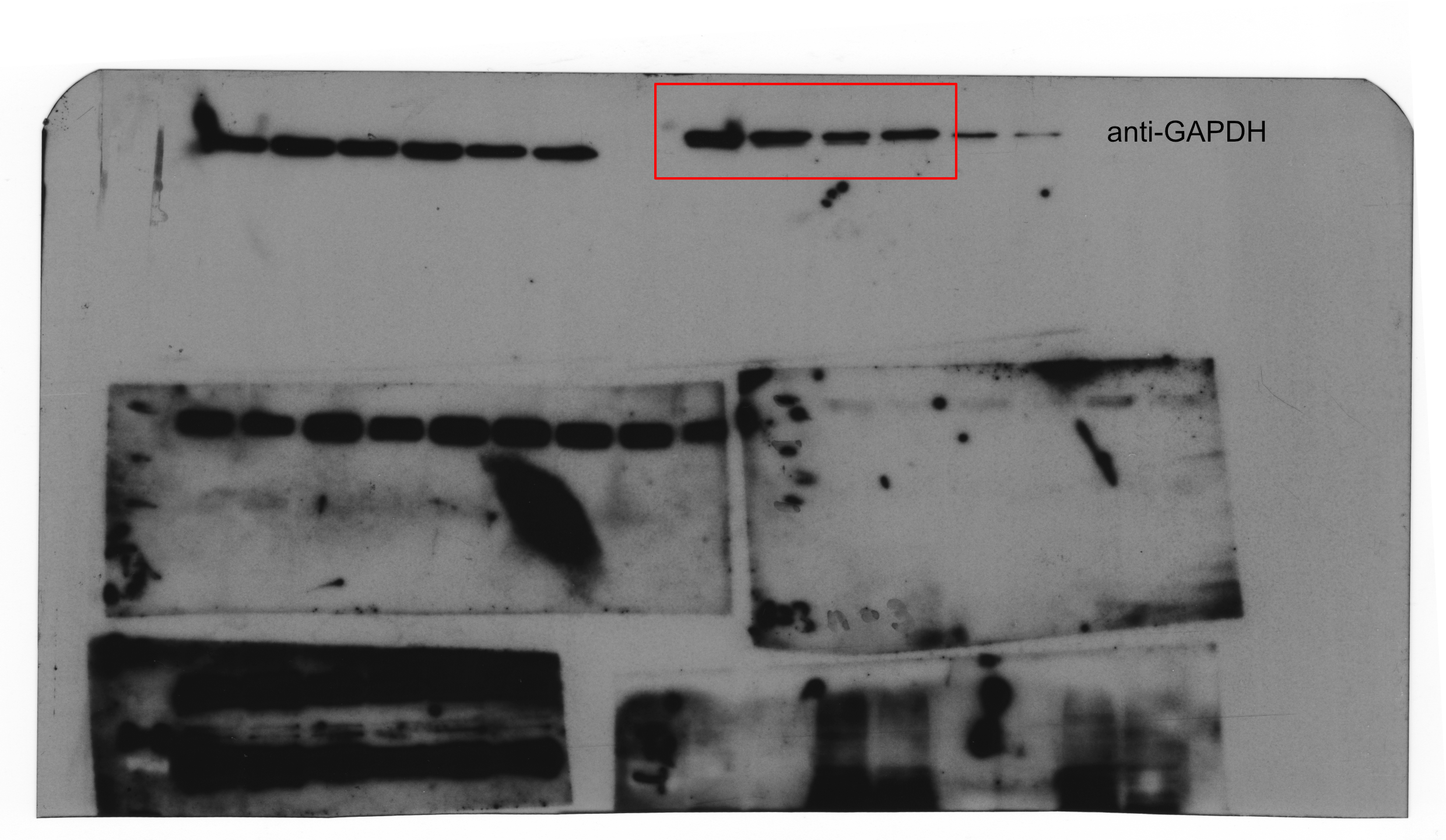

Supplement: Supplementary file 6 — Source data Fig. 3 [file 44318_2025_608_MOESM6_ESM.zip › Figure 3/3B/western GAPDH.tiff]

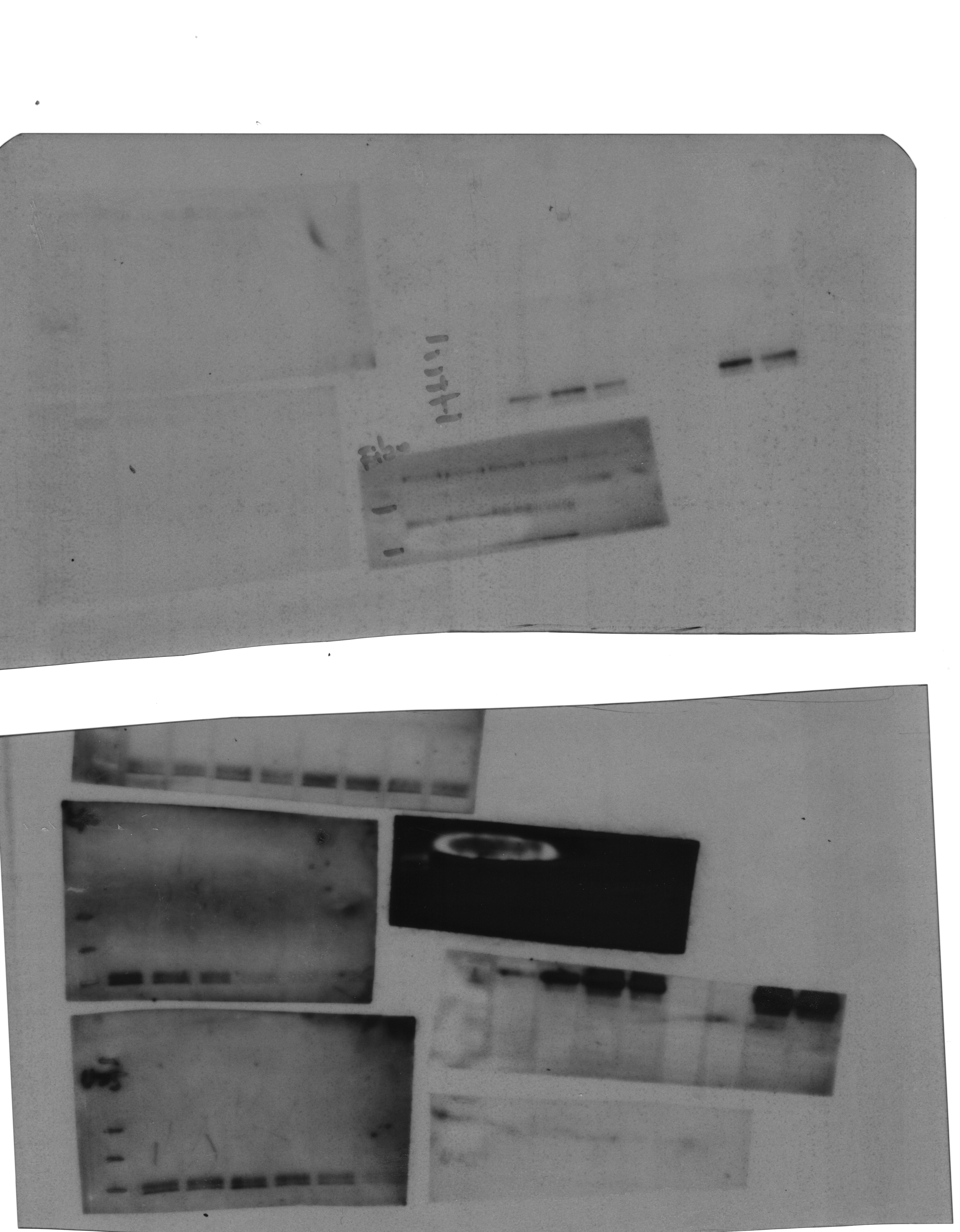

Supplement: Supplementary file 6 — Source data Fig. 3 [file 44318_2025_608_MOESM6_ESM.zip › Figure 3/3B/western SLC7A5.TIF]

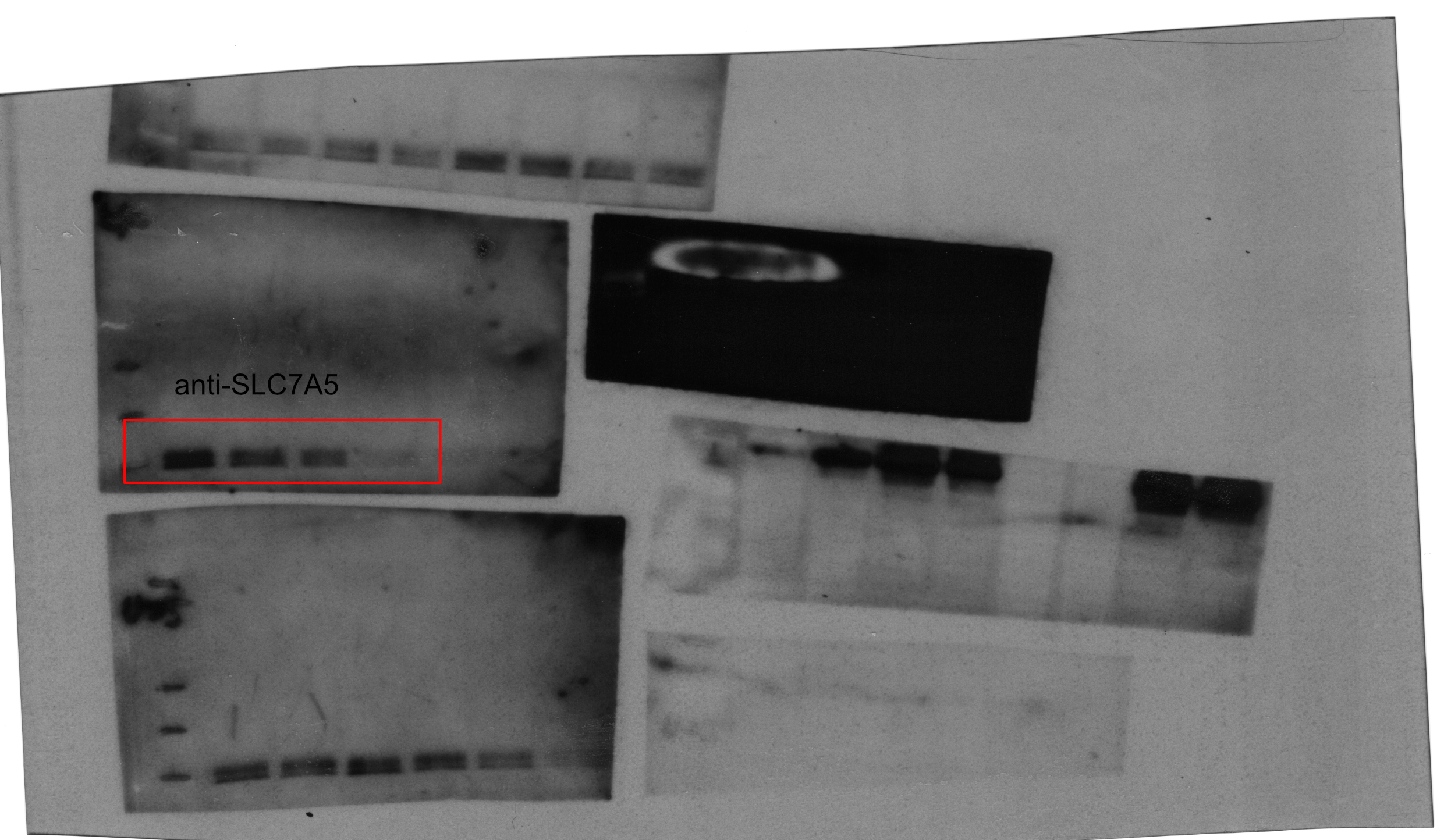

Supplement: Supplementary file 6 — Source data Fig. 3 [file 44318_2025_608_MOESM6_ESM.zip › Figure 3/3B/western SLC7A5.tiff]

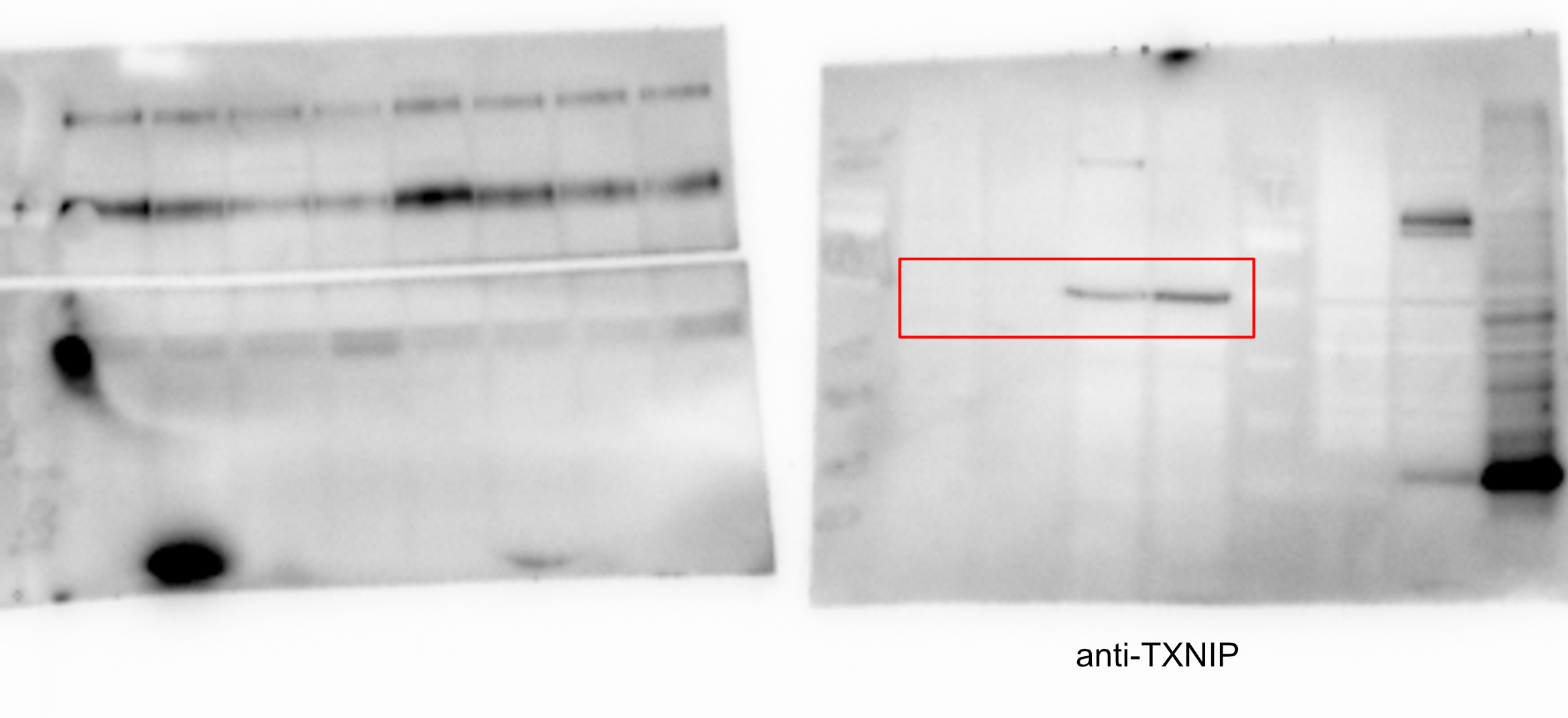

Supplement: Supplementary file 6 — Source data Fig. 3 [file 44318_2025_608_MOESM6_ESM.zip › Figure 3/3B/western TXNIP.tiff]

Figure 3D

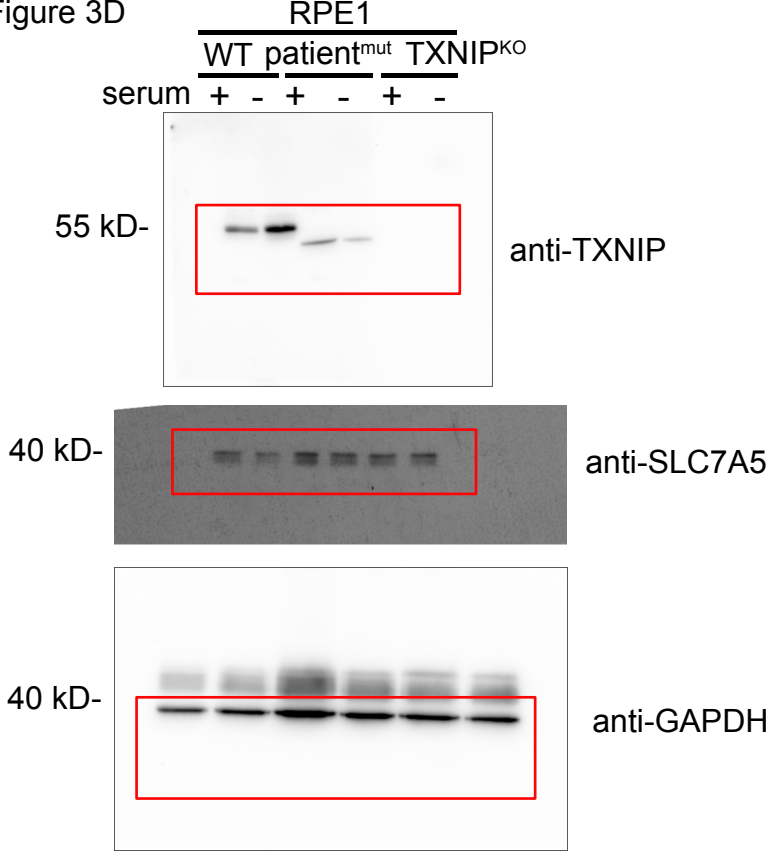

Supplement: Supplementary file 6 — Source data Fig. 3 [file 44318_2025_608_MOESM6_ESM.zip › Figure 3/3D/Figure 3D.pdf]

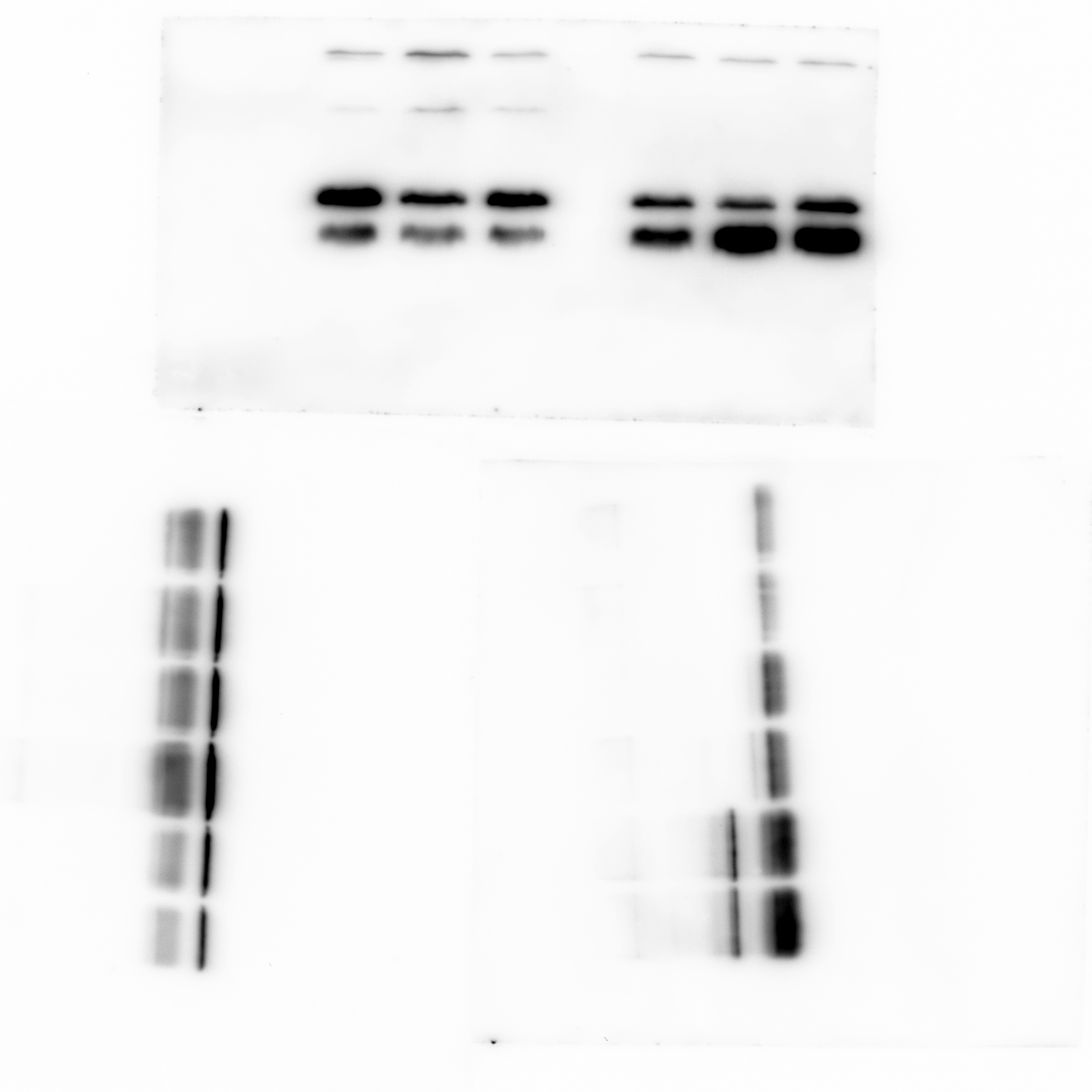

Supplement: Supplementary file 6 — Source data Fig. 3 [file 44318_2025_608_MOESM6_ESM.zip › Figure 3/3D/western GAPDH.TIF]

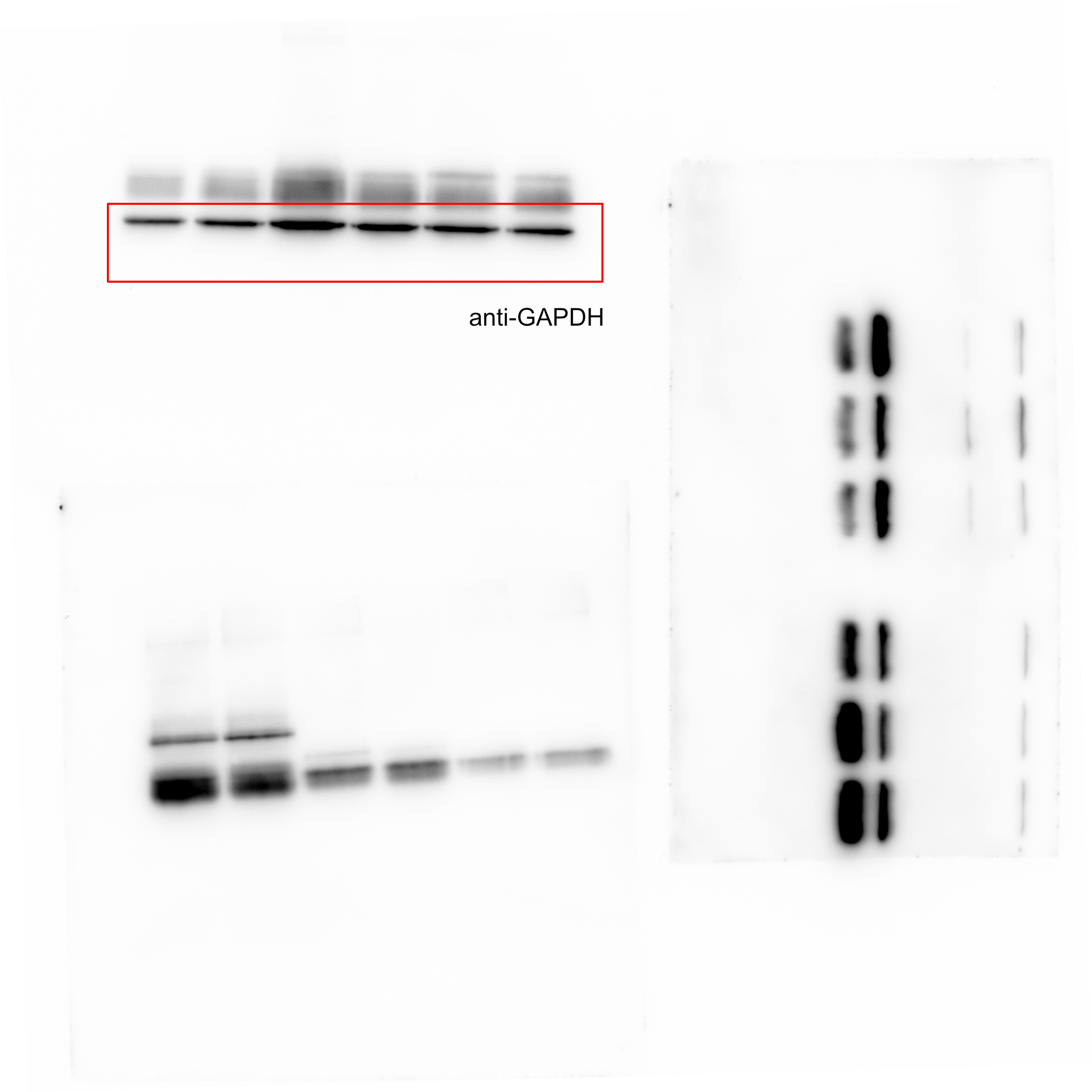

Supplement: Supplementary file 6 — Source data Fig. 3 [file 44318_2025_608_MOESM6_ESM.zip › Figure 3/3D/western GAPDH.tiff]

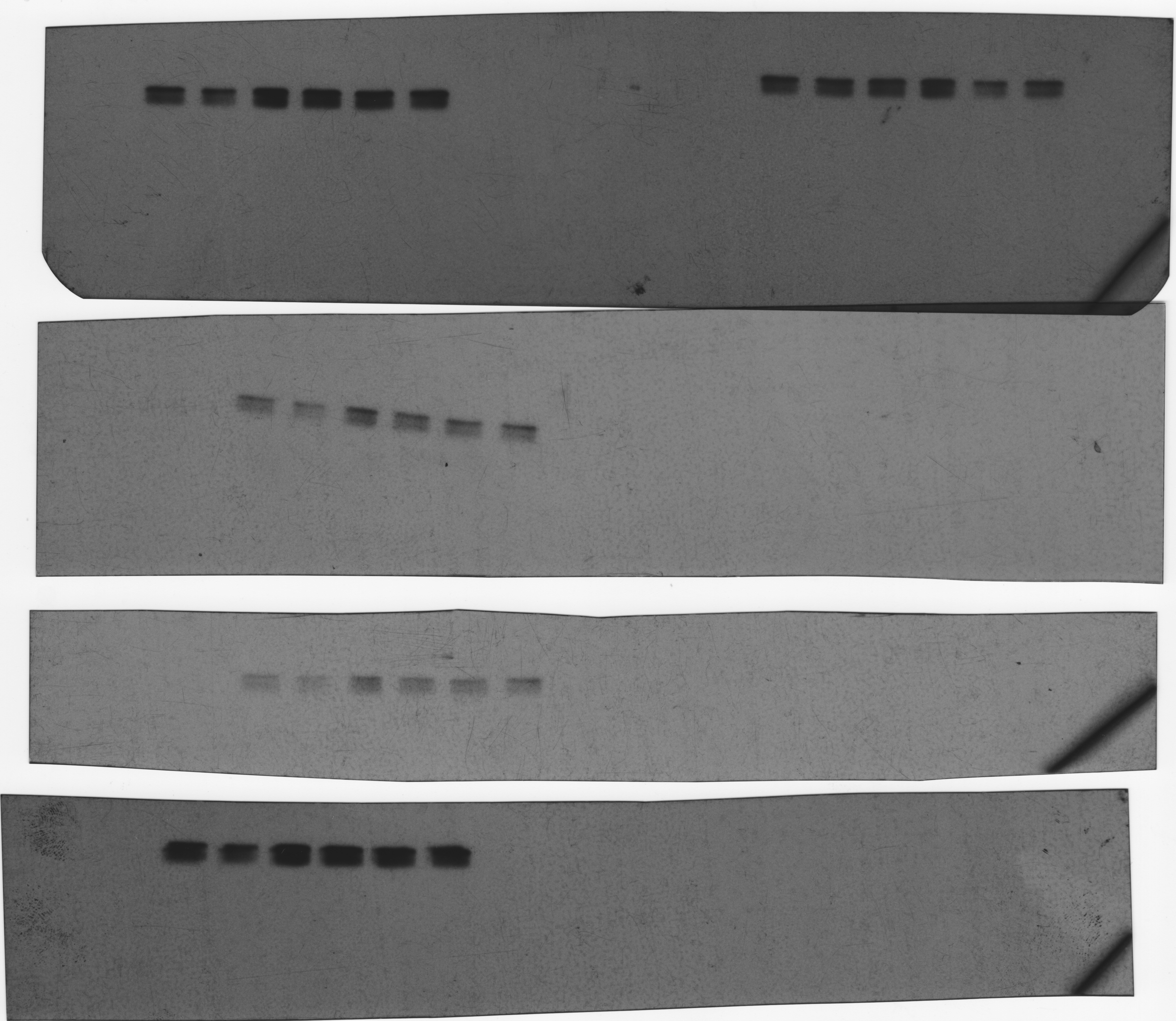

Supplement: Supplementary file 6 — Source data Fig. 3 [file 44318_2025_608_MOESM6_ESM.zip › Figure 3/3D/western SLC7A5.TIF]

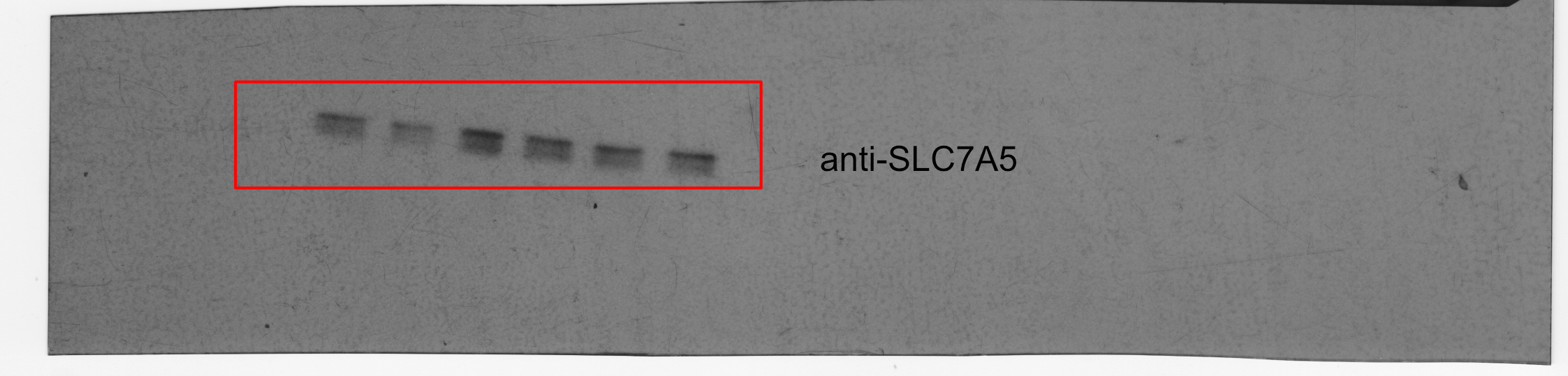

Supplement: Supplementary file 6 — Source data Fig. 3 [file 44318_2025_608_MOESM6_ESM.zip › Figure 3/3D/western SLC7A5.tiff]

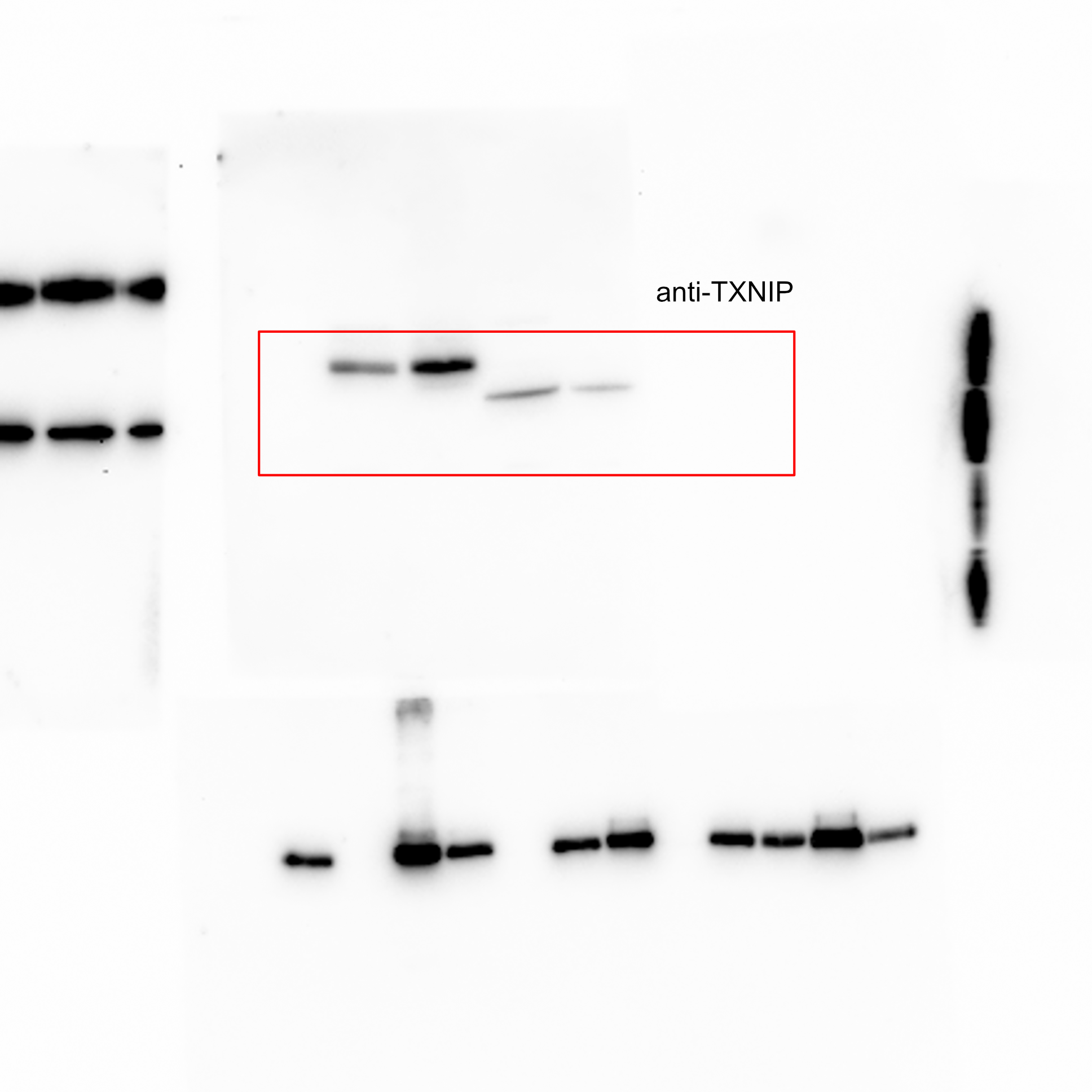

Supplement: Supplementary file 6 — Source data Fig. 3 [file 44318_2025_608_MOESM6_ESM.zip › Figure 3/3D/western TXNIP.tiff]

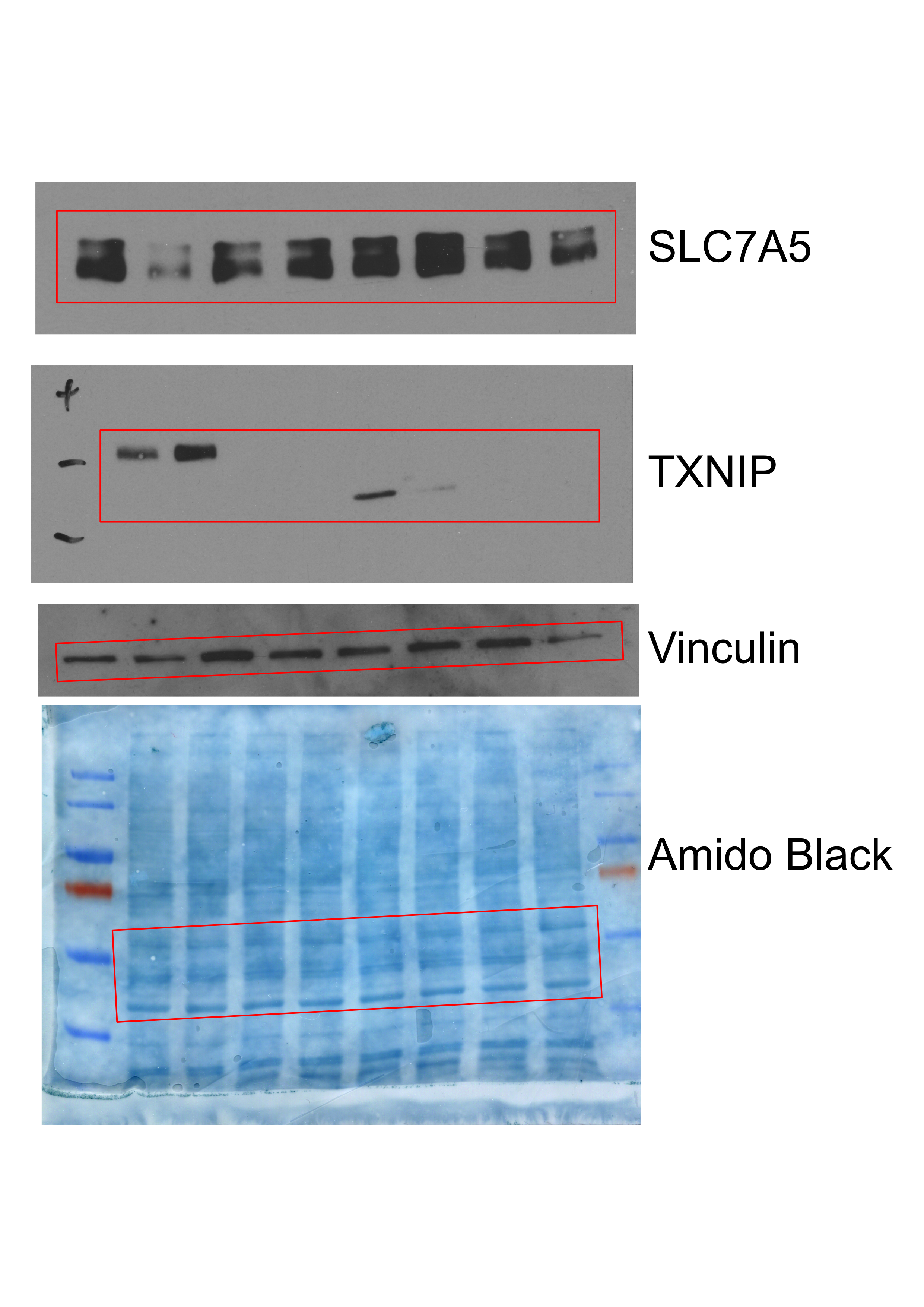

Supplement: Supplementary file 6 — Source data Fig. 3 [file 44318_2025_608_MOESM6_ESM.zip › Figure 3/new 3D/Figure3D-westernblot.tiff]

Figure 4B

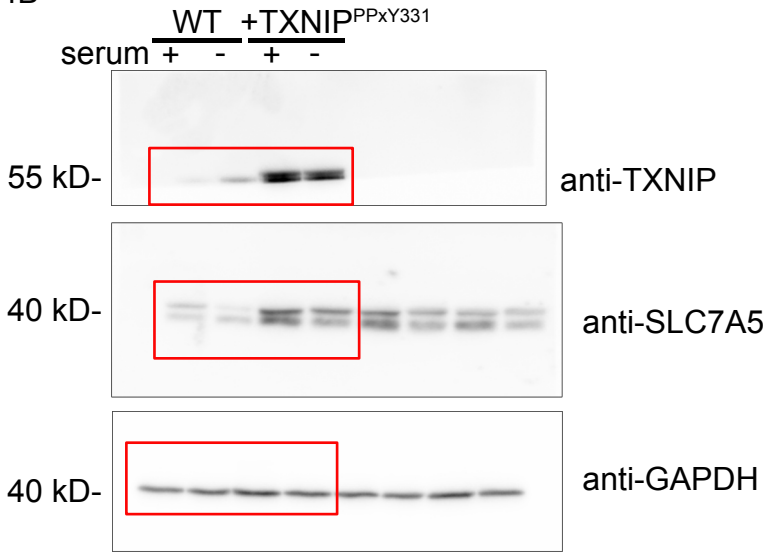

Supplement: Supplementary file 7 — Source data Fig. 4 [file 44318_2025_608_MOESM7_ESM.zip › Figure 4/4B/Figure 4B.pdf]

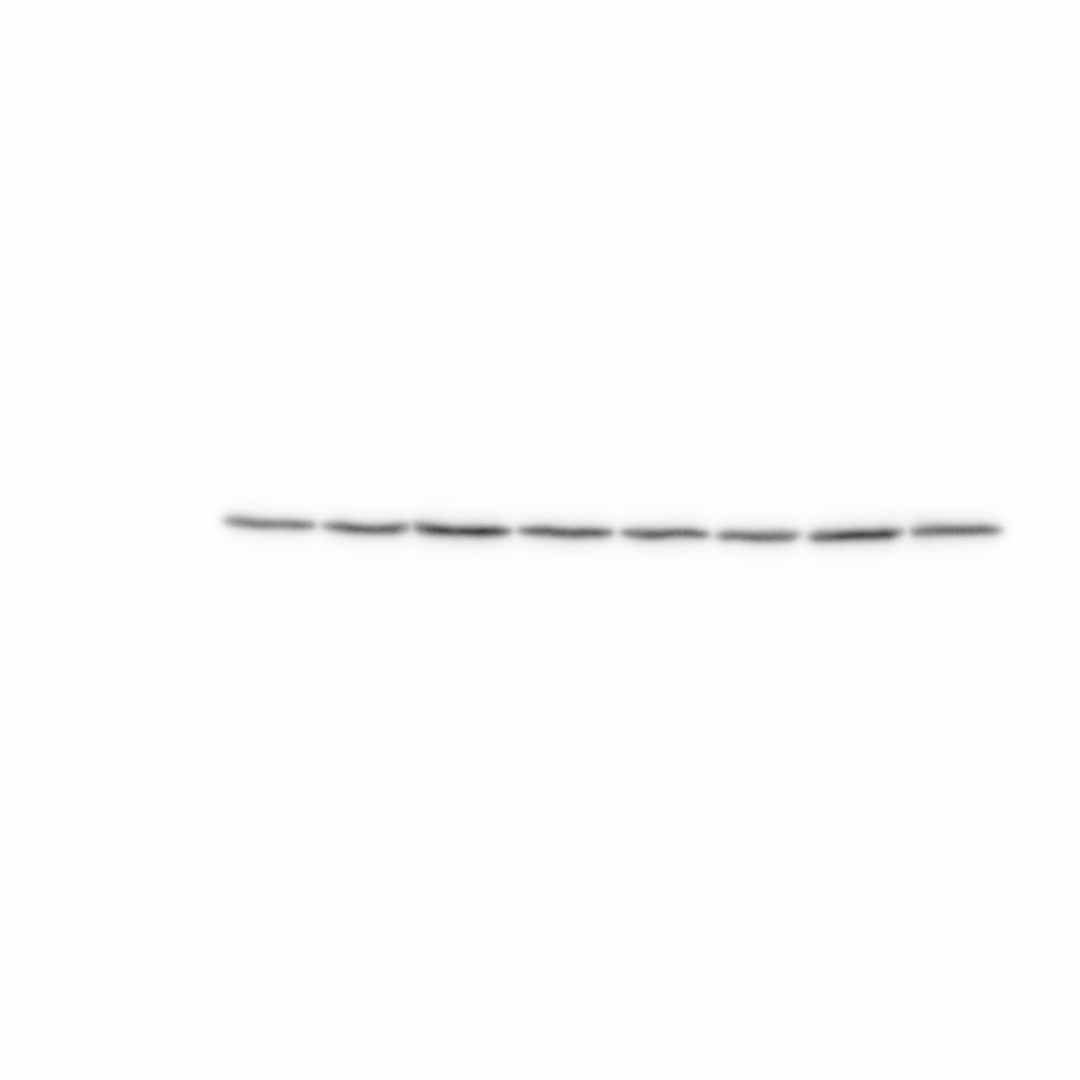

Supplement: Supplementary file 7 — Source data Fig. 4 [file 44318_2025_608_MOESM7_ESM.zip › Figure 4/4B/western GAPDH.tif]

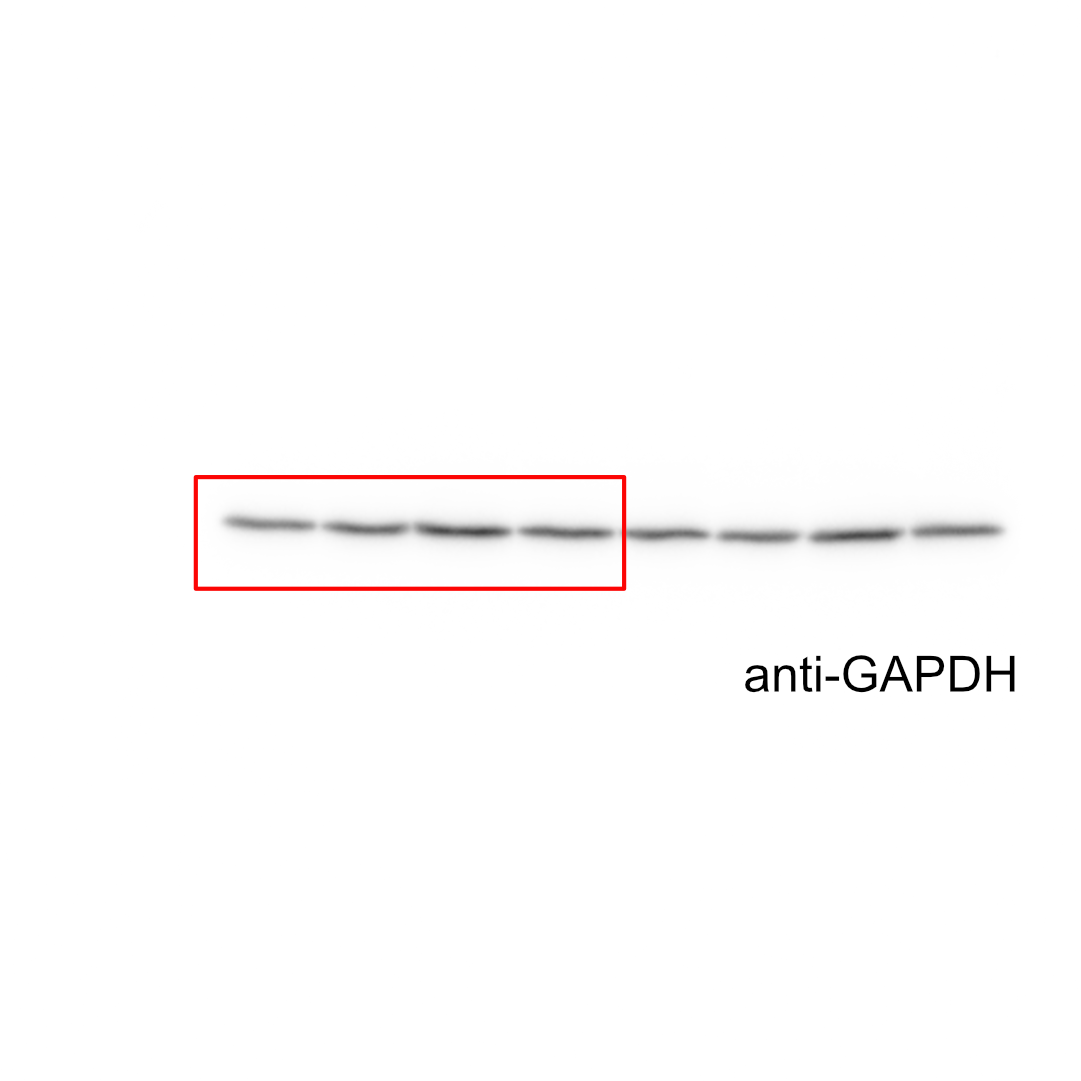

Supplement: Supplementary file 7 — Source data Fig. 4 [file 44318_2025_608_MOESM7_ESM.zip › Figure 4/4B/western GAPDH.tiff]

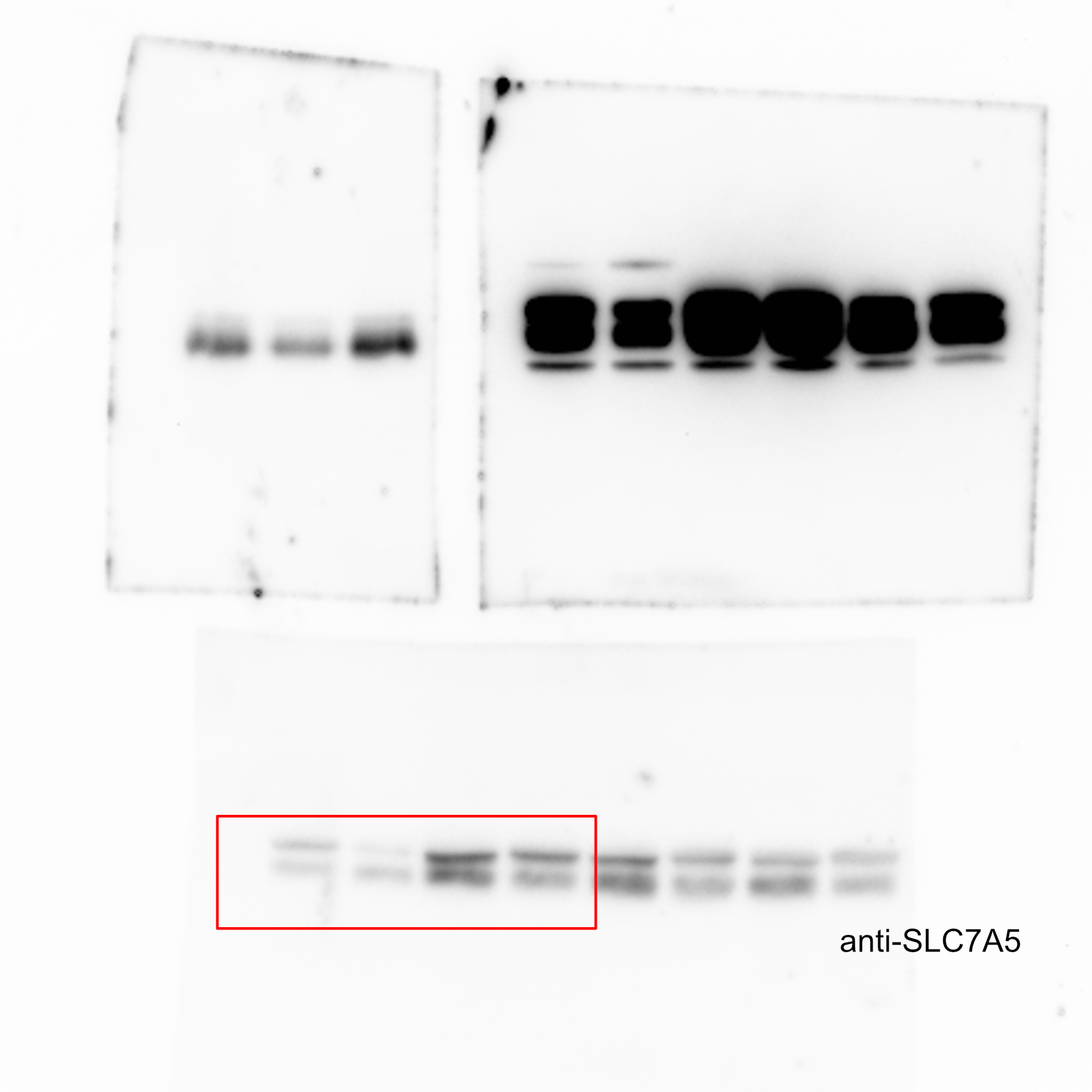

Supplement: Supplementary file 7 — Source data Fig. 4 [file 44318_2025_608_MOESM7_ESM.zip › Figure 4/4B/western SLC7A5.tiff]

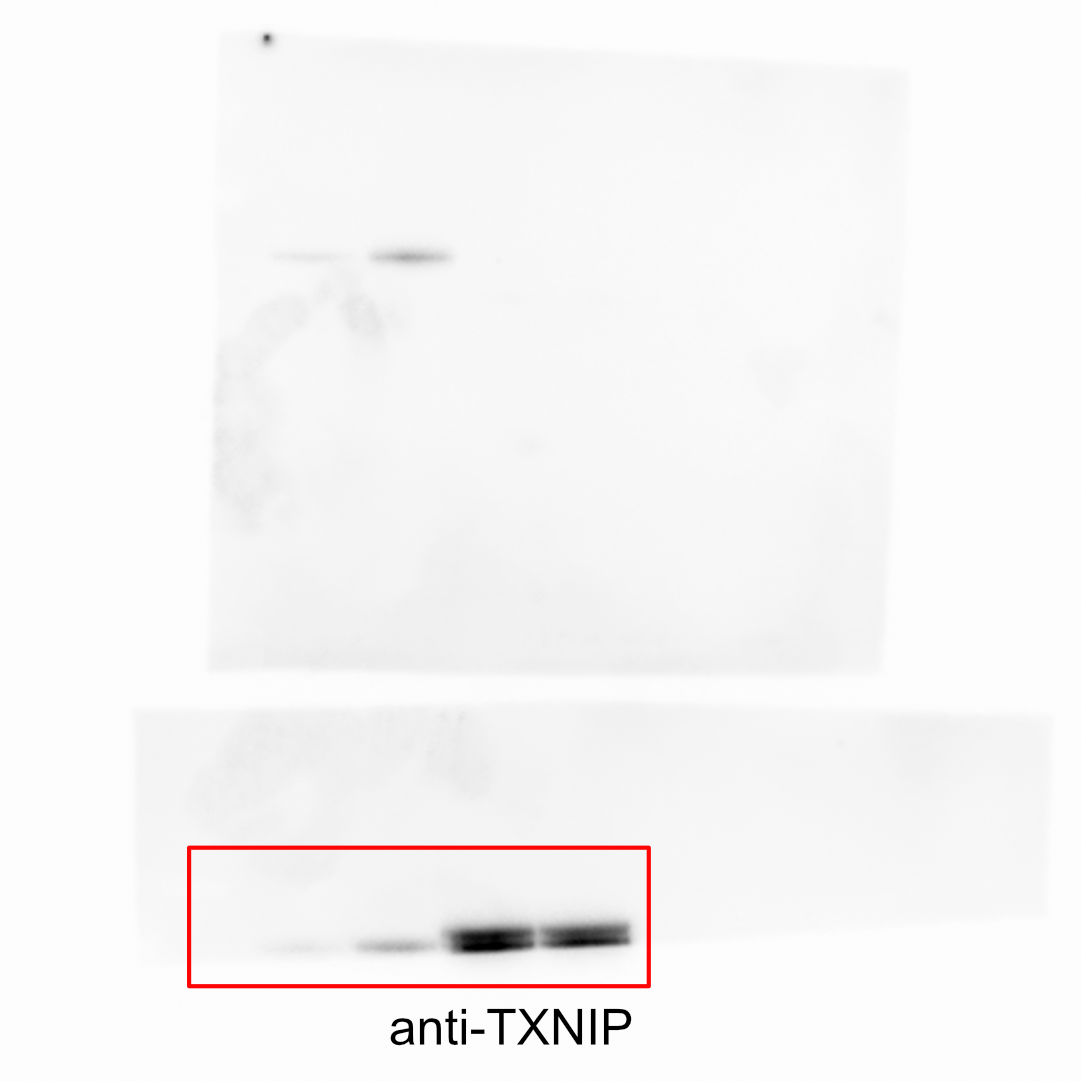

Supplement: Supplementary file 7 — Source data Fig. 4 [file 44318_2025_608_MOESM7_ESM.zip › Figure 4/4B/western TXNIP.tiff]

Figure 4G

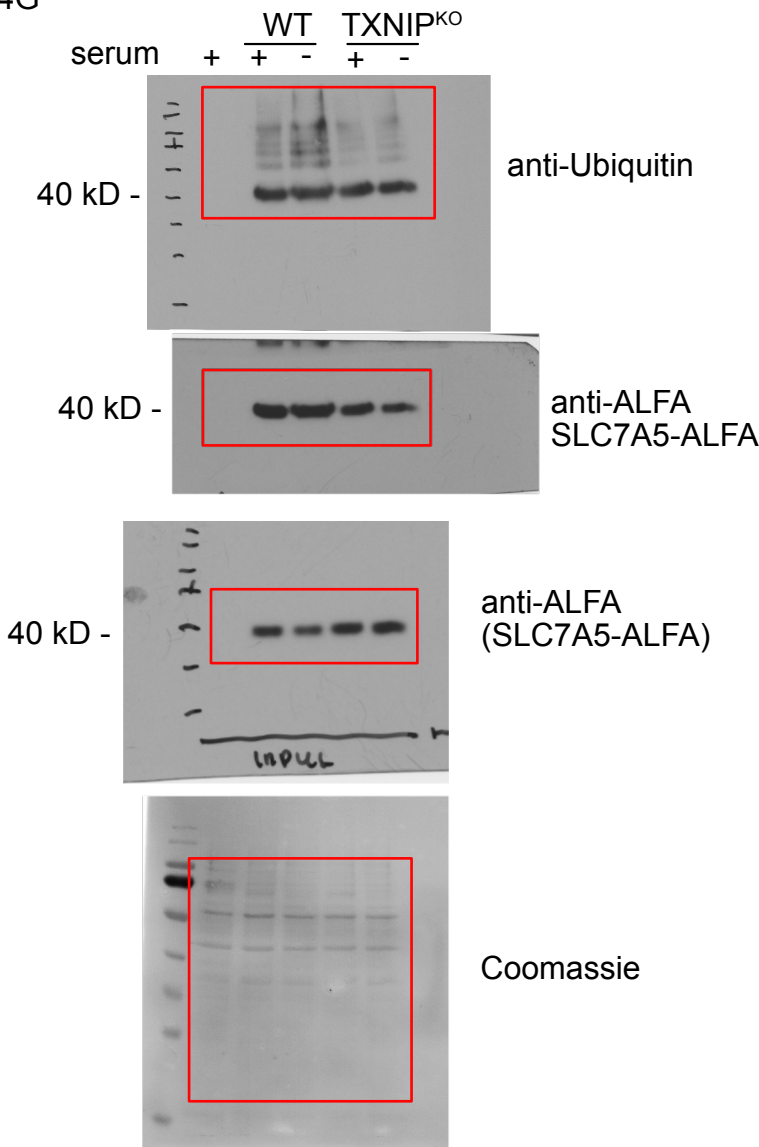

Supplement: Supplementary file 7 — Source data Fig. 4 [file 44318_2025_608_MOESM7_ESM.zip › Figure 4/4G/Figure 4G.pdf]

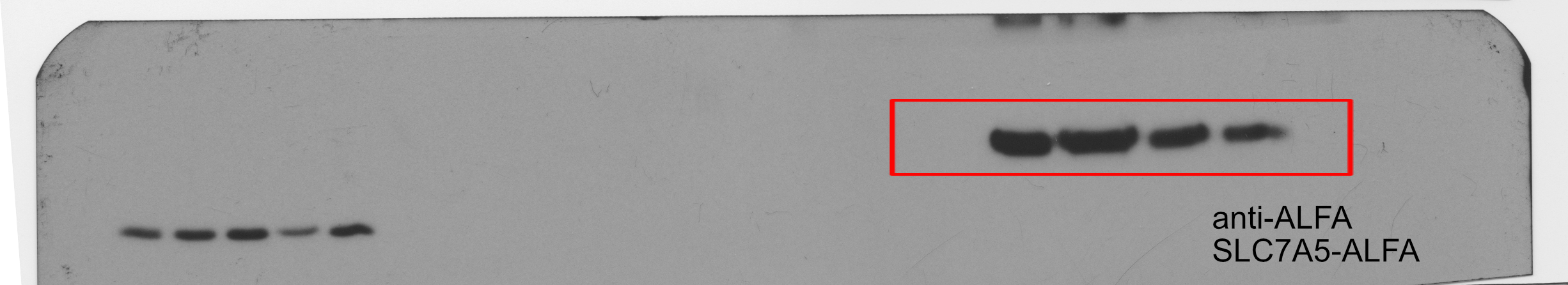

Supplement: Supplementary file 7 — Source data Fig. 4 [file 44318_2025_608_MOESM7_ESM.zip › Figure 4/4G/western ALFA.tiff]

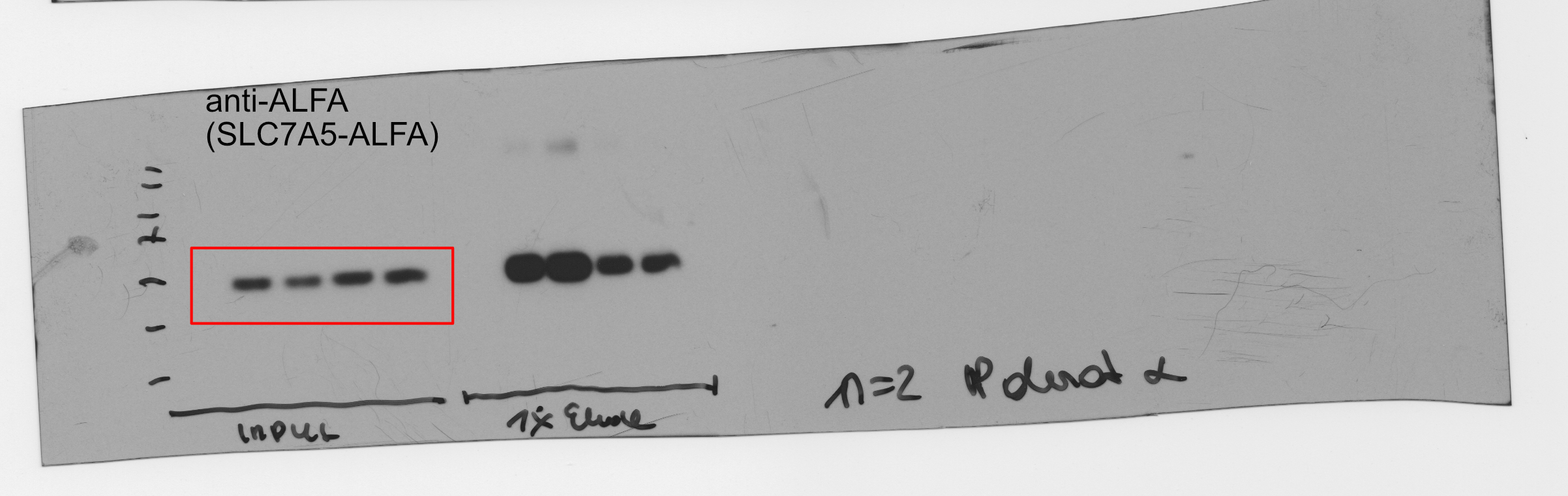

Supplement: Supplementary file 7 — Source data Fig. 4 [file 44318_2025_608_MOESM7_ESM.zip › Figure 4/4G/western ALFA_input.tiff]

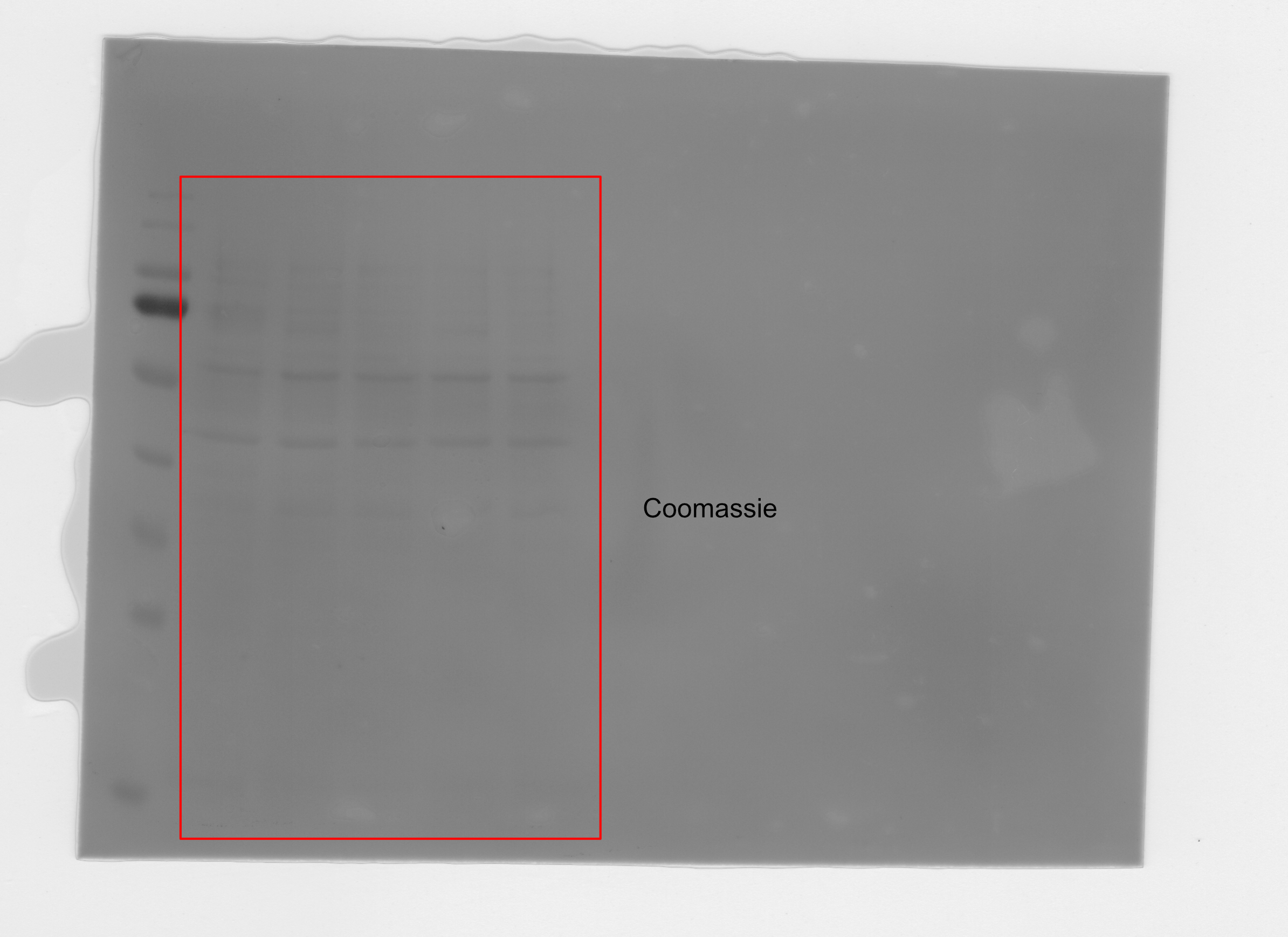

Supplement: Supplementary file 7 — Source data Fig. 4 [file 44318_2025_608_MOESM7_ESM.zip › Figure 4/4G/western Coomassie.tiff]

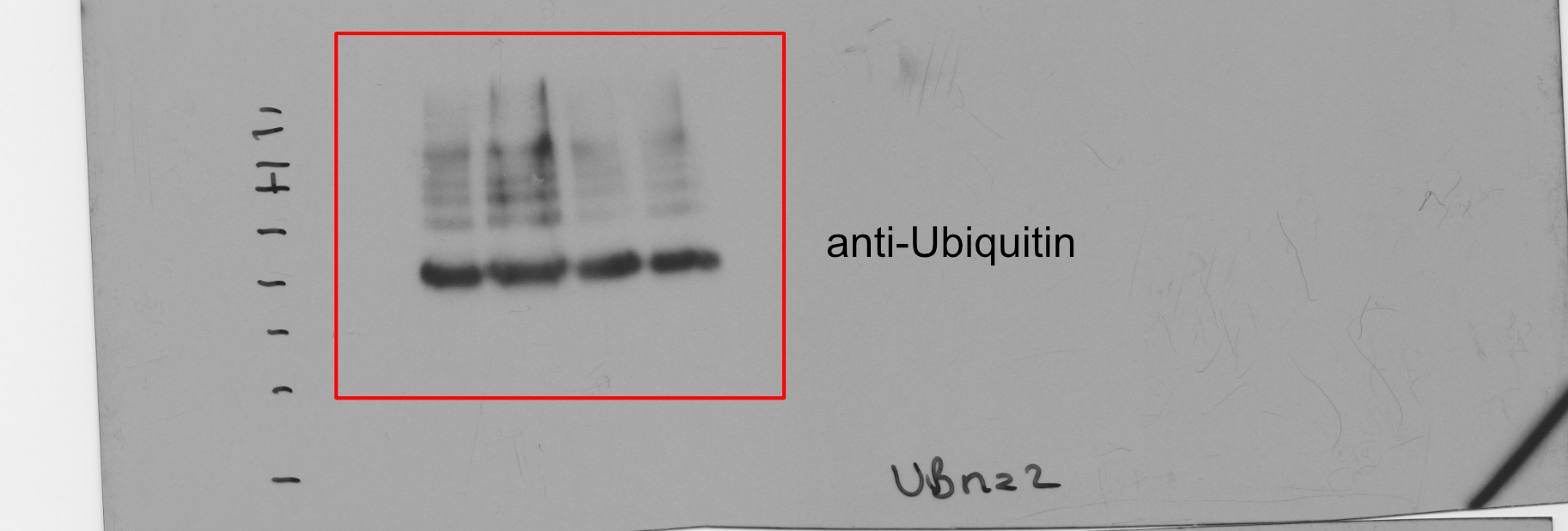

Supplement: Supplementary file 7 — Source data Fig. 4 [file 44318_2025_608_MOESM7_ESM.zip › Figure 4/4G/western ubiquitin.tiff]

Figure 5A

|       |   |   |   |   |
|-------|---|---|---|---|
| MK    | - | - | + | - |
| PD    | - | - | - | + |
| serum | + | - | + | + |

40 kD-

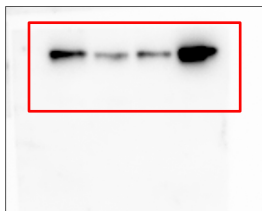

anti-SLC7A5

55 kD-

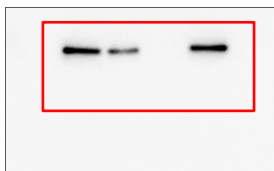

anti-p-AKTS473

55 kD-

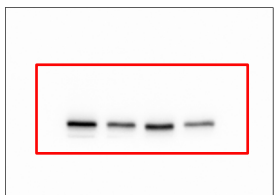

anti-AKT

40 kD-

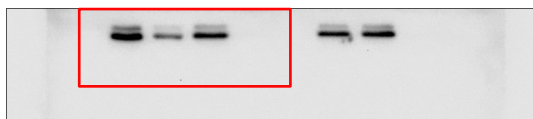

anti-p-ERK1/2

40 kD-

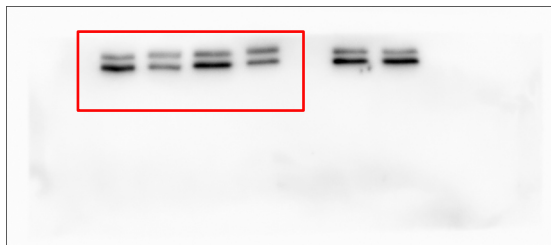

anti-ERK1/2

40 kD-

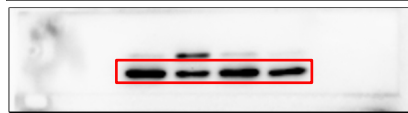

anti-GAPDH

Supplement: Supplementary file 8 — Source data Fig. 5 [file 44318_2025_608_MOESM8_ESM.zip › Figure 5/5A/Figure 5A.pdf]

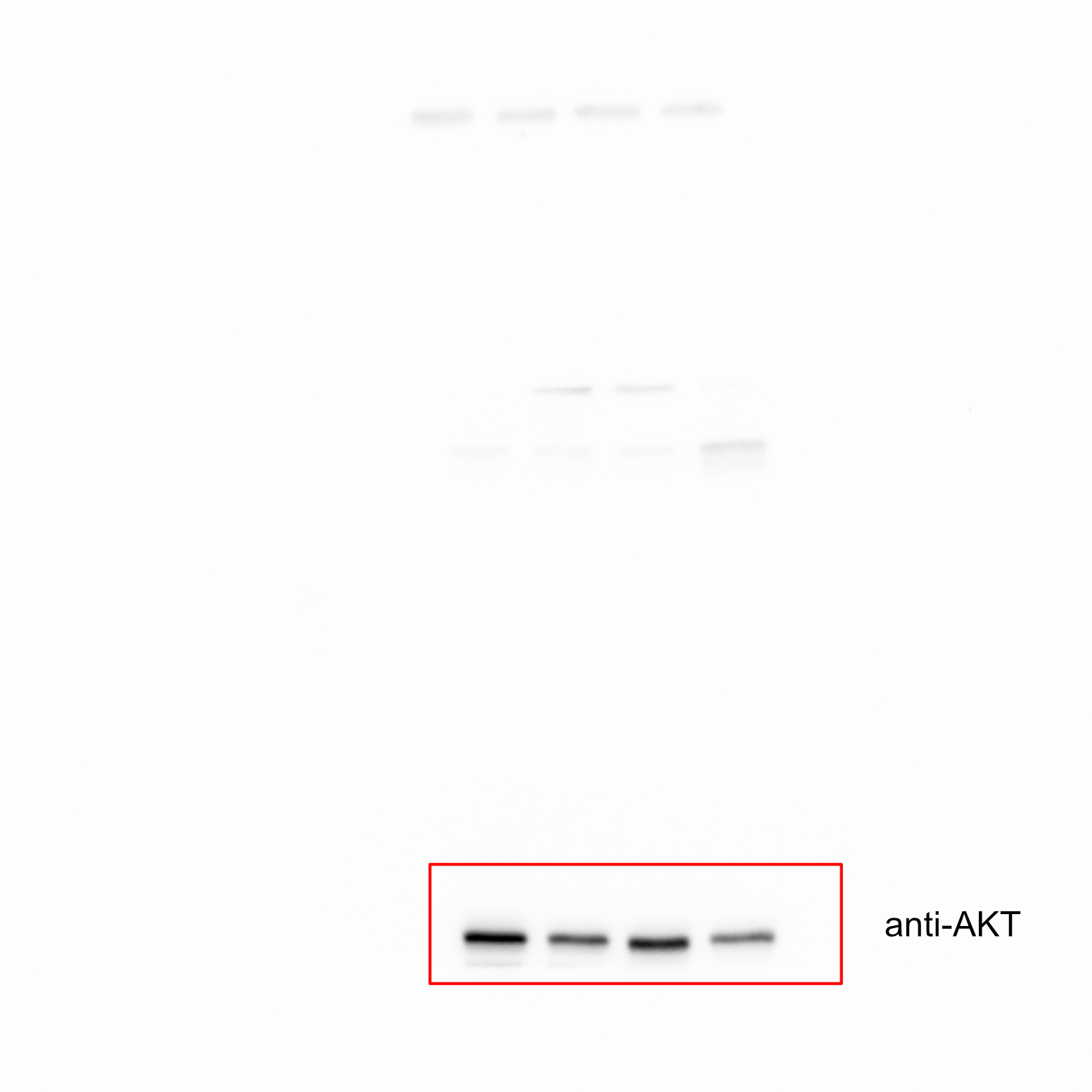

Supplement: Supplementary file 8 — Source data Fig. 5 [file 44318_2025_608_MOESM8_ESM.zip › Figure 5/5A/western AKT.tiff]

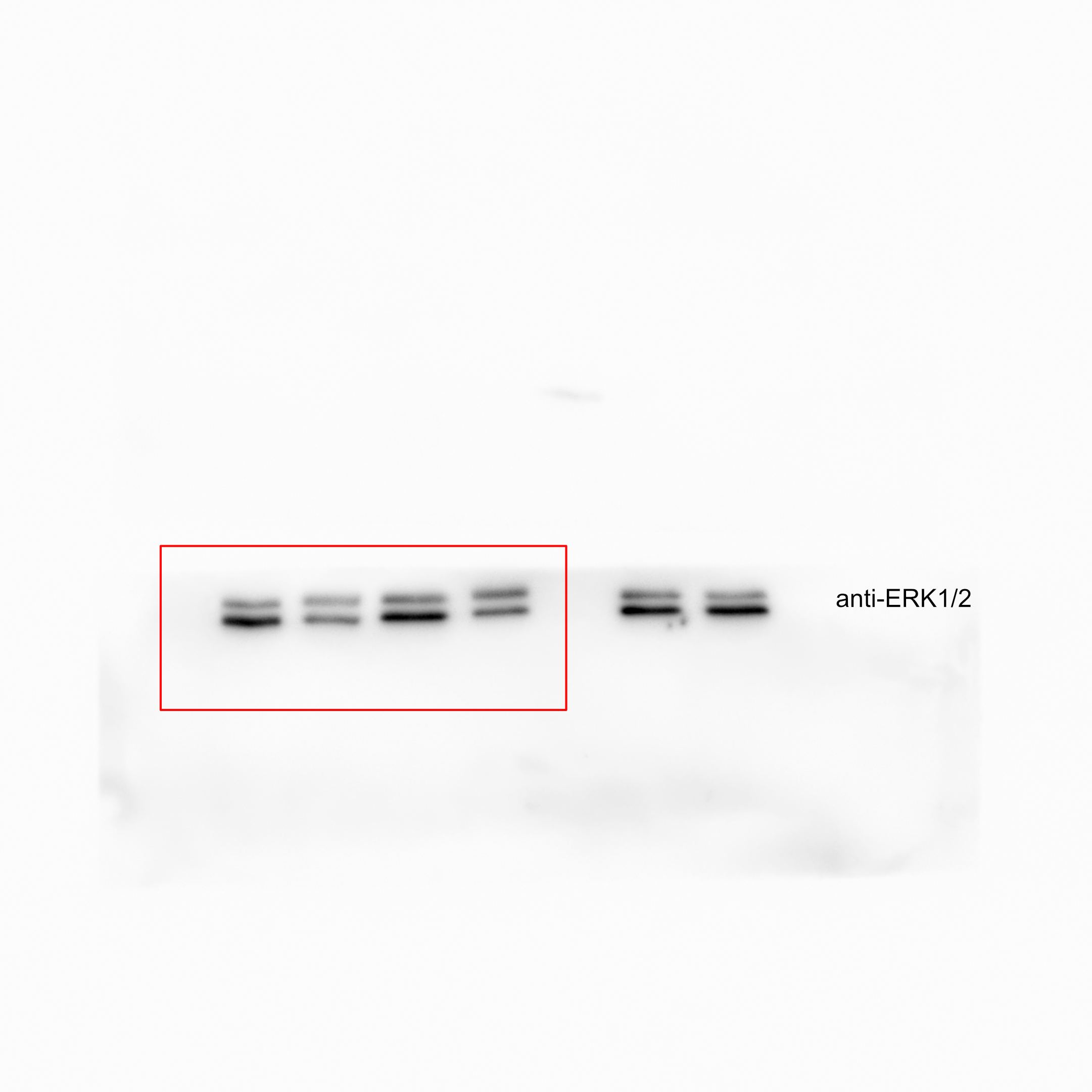

Supplement: Supplementary file 8 — Source data Fig. 5 [file 44318_2025_608_MOESM8_ESM.zip › Figure 5/5A/western ERK.tiff]

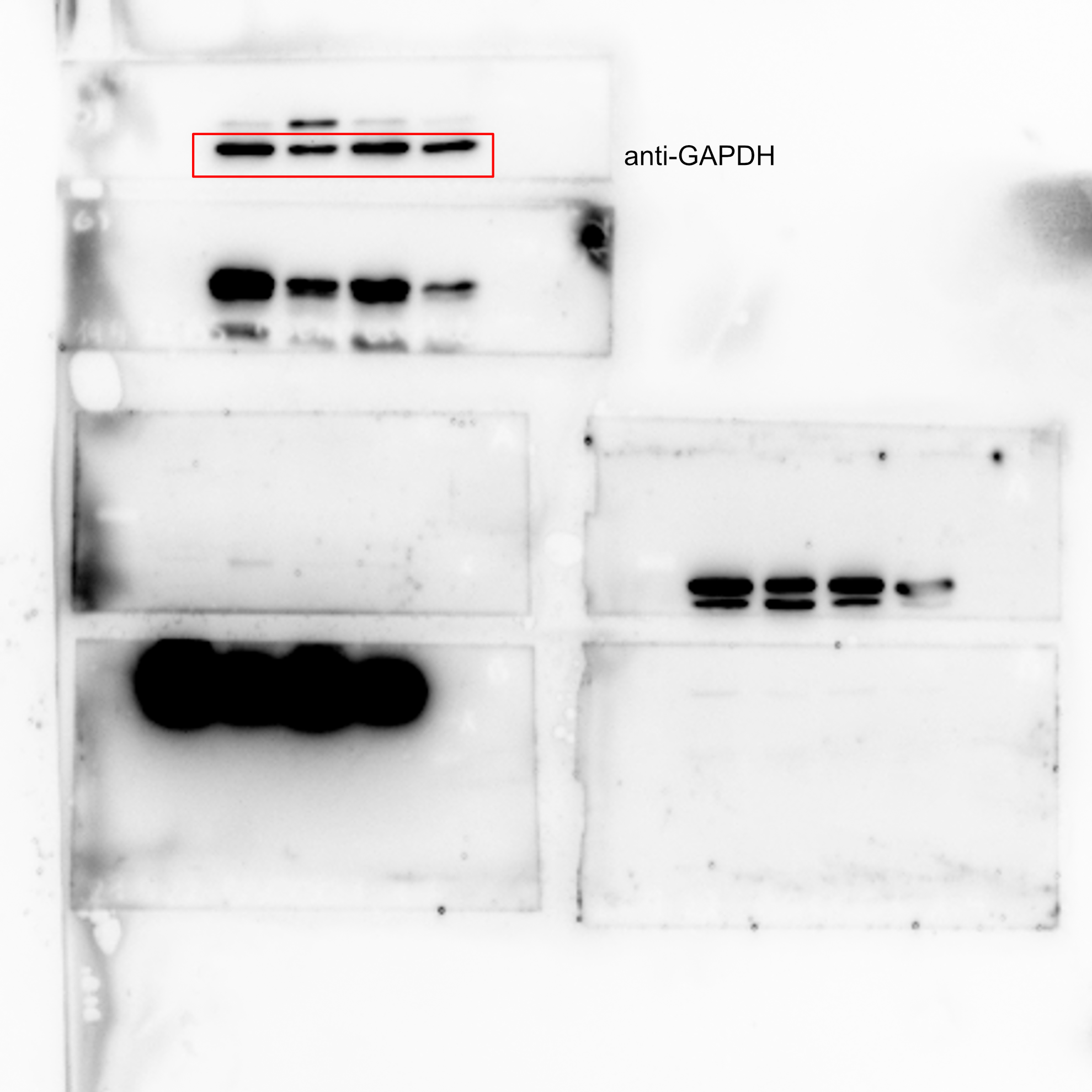

Supplement: Supplementary file 8 — Source data Fig. 5 [file 44318_2025_608_MOESM8_ESM.zip › Figure 5/5A/western GAPDH.tiff]

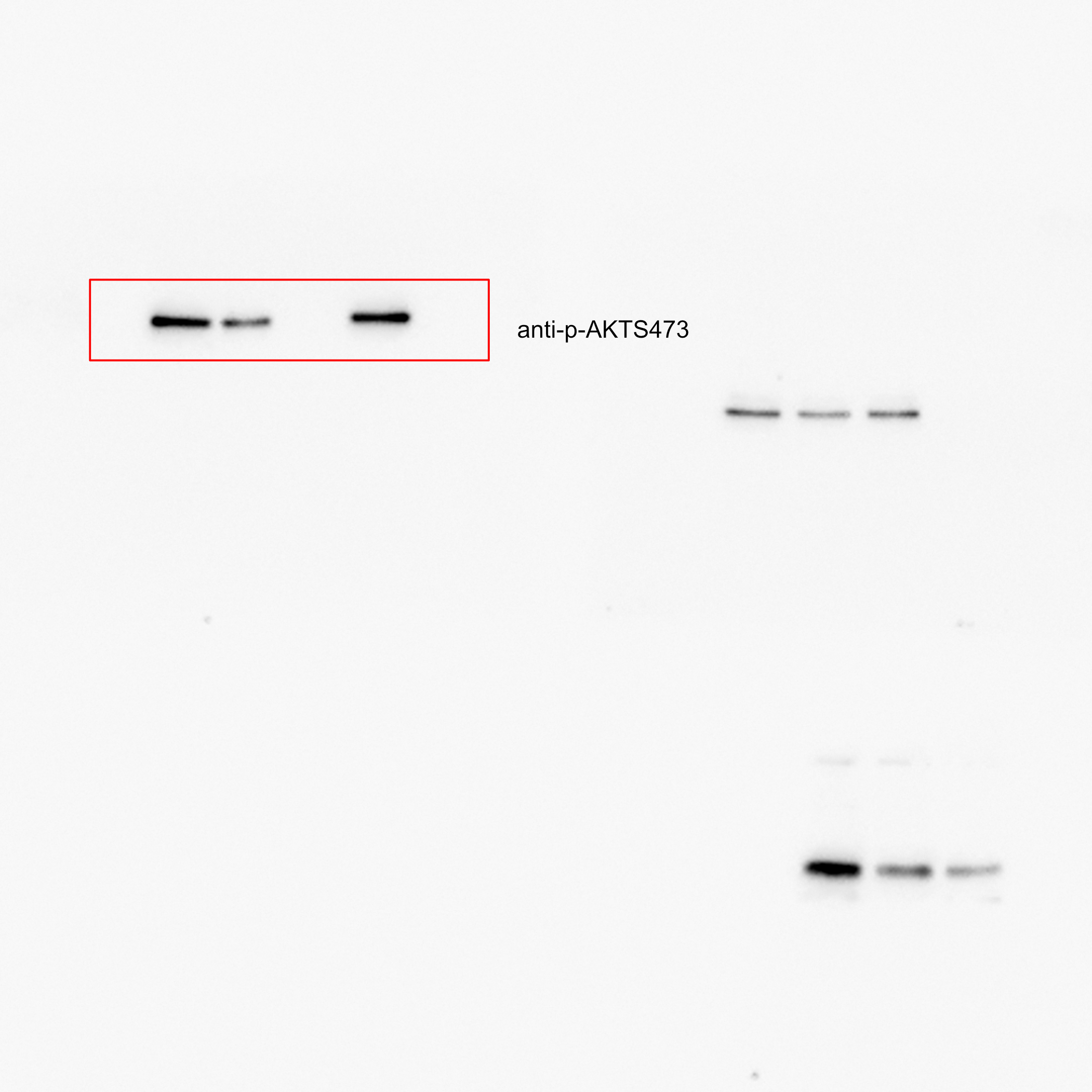

Supplement: Supplementary file 8 — Source data Fig. 5 [file 44318_2025_608_MOESM8_ESM.zip › Figure 5/5A/western pAKT.tiff]

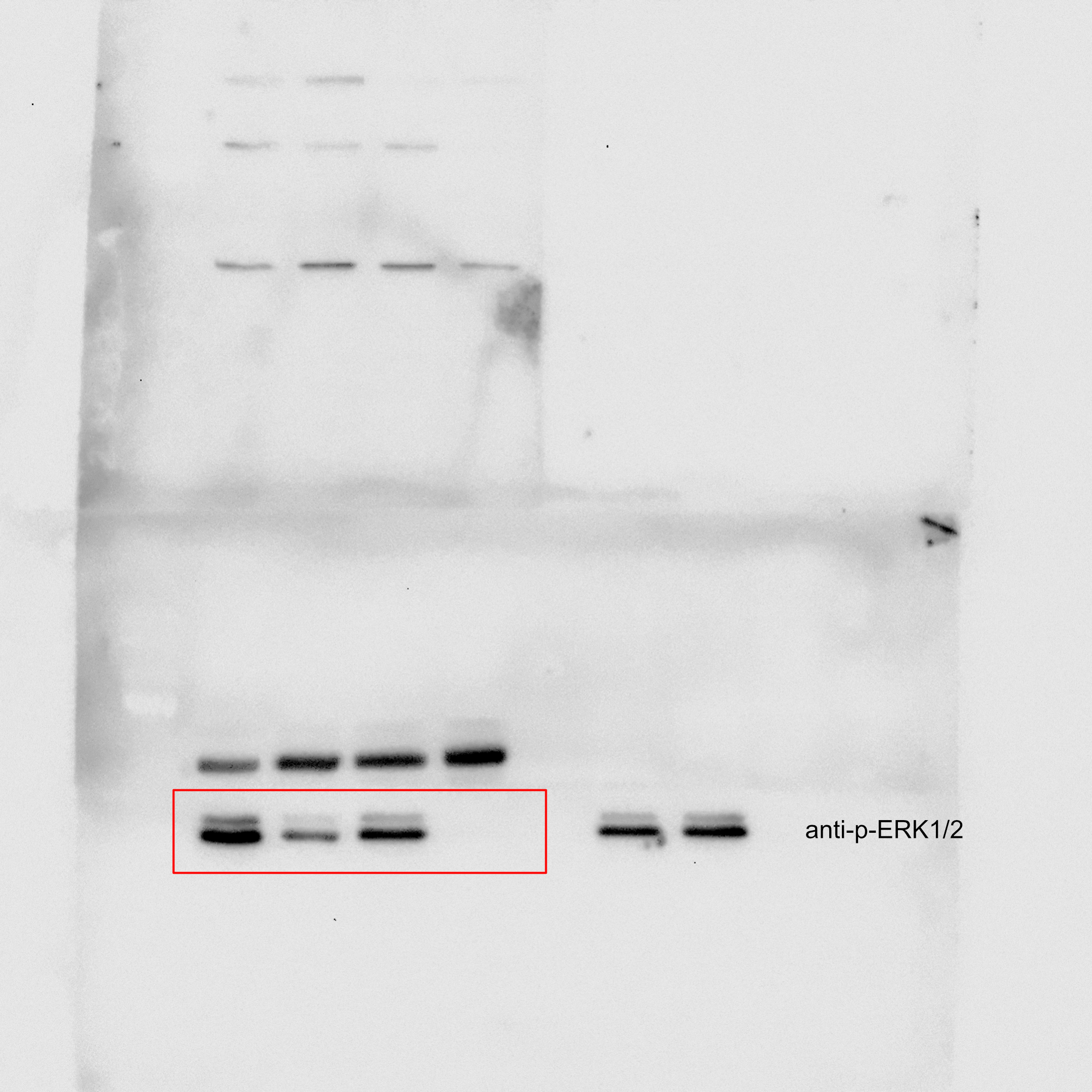

Supplement: Supplementary file 8 — Source data Fig. 5 [file 44318_2025_608_MOESM8_ESM.zip › Figure 5/5A/western pERK.tiff]

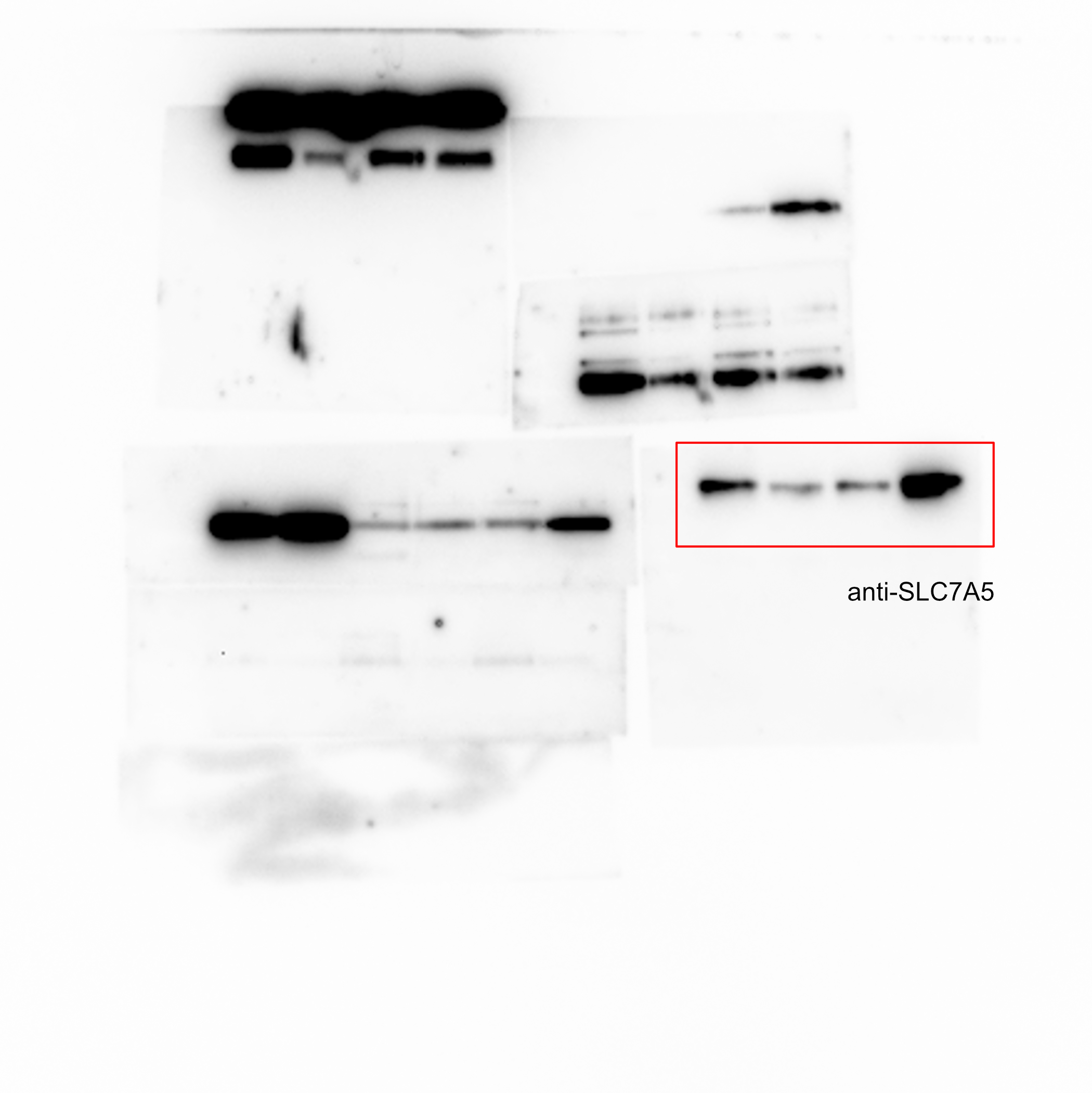

Supplement: Supplementary file 8 — Source data Fig. 5 [file 44318_2025_608_MOESM8_ESM.zip › Figure 5/5A/western SLC7A5.tiff]

Figure 5c

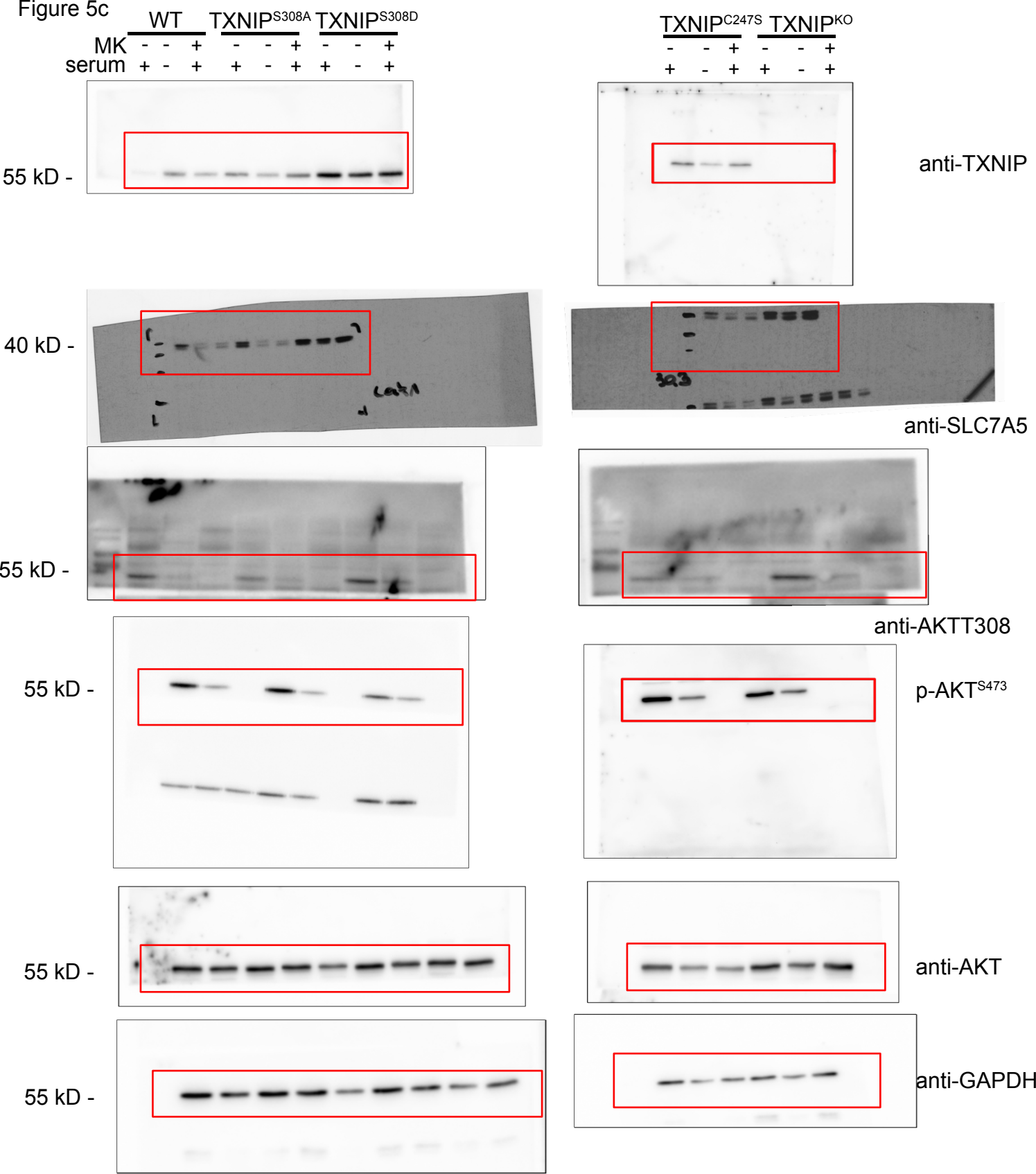

Supplement: Supplementary file 8 — Source data Fig. 5 [file 44318_2025_608_MOESM8_ESM.zip › Figure 5/5C/Figure 5c.pdf]

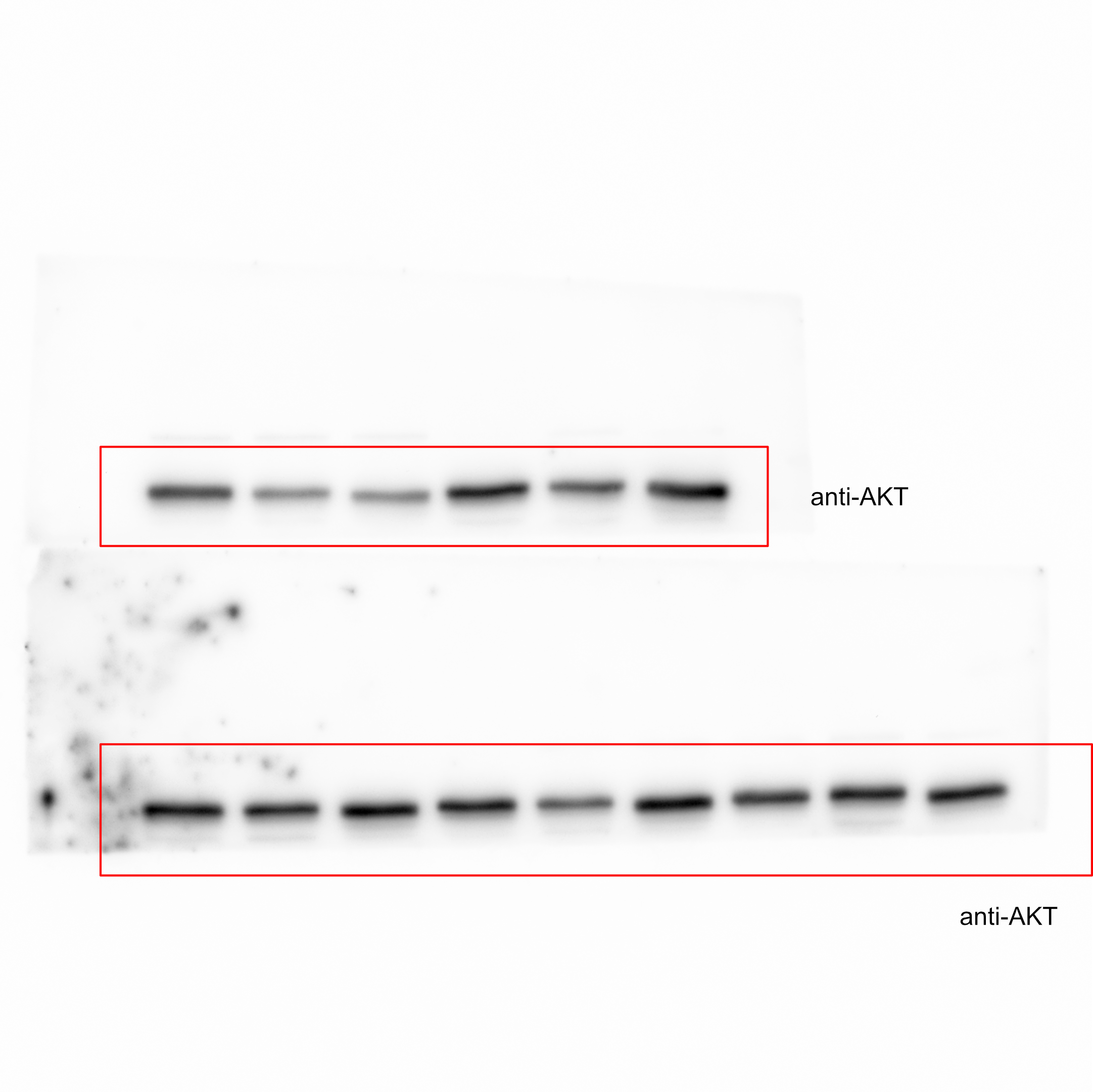

Supplement: Supplementary file 8 — Source data Fig. 5 [file 44318_2025_608_MOESM8_ESM.zip › Figure 5/5C/western AKT.tiff]

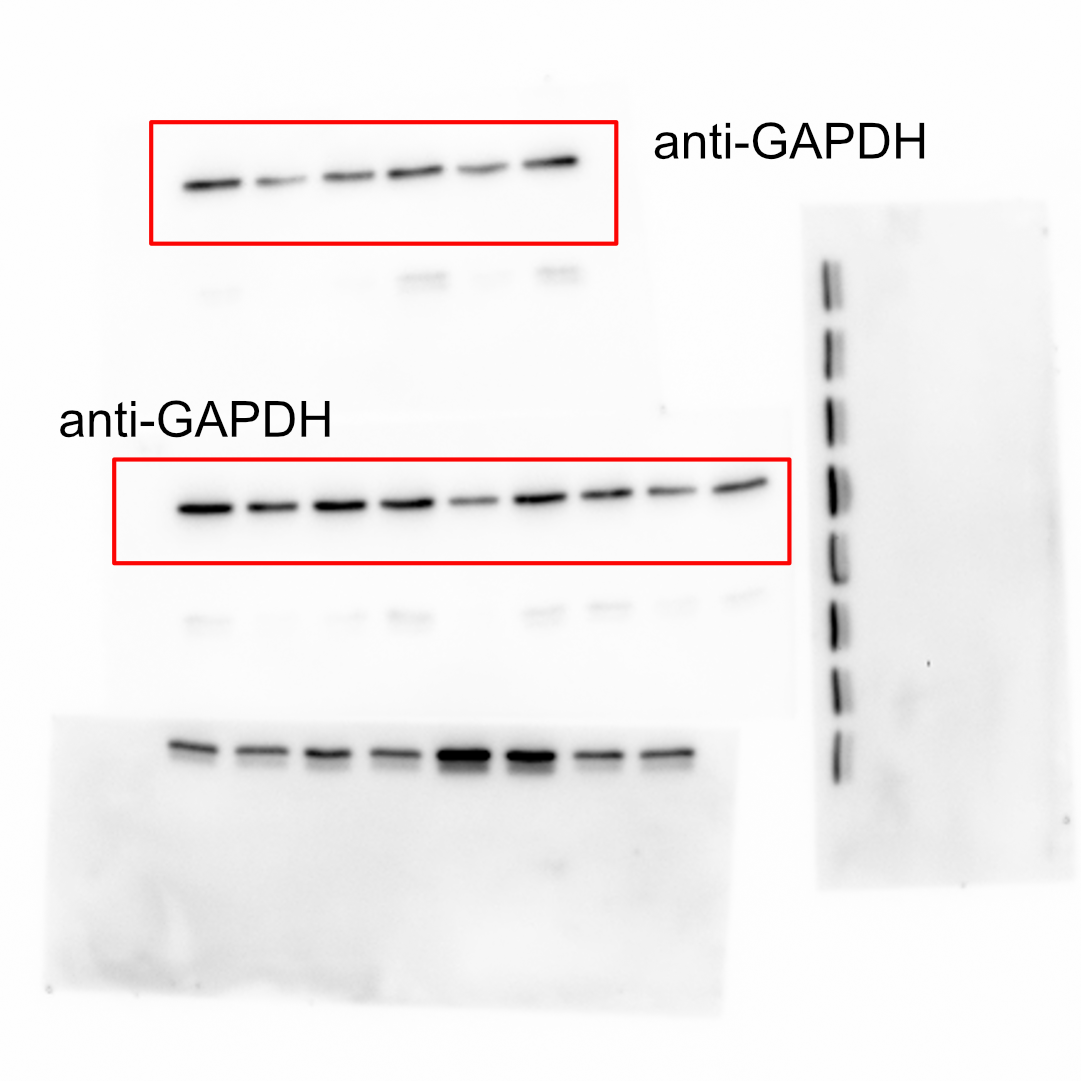

Supplement: Supplementary file 8 — Source data Fig. 5 [file 44318_2025_608_MOESM8_ESM.zip › Figure 5/5C/western GAPDH.tiff]

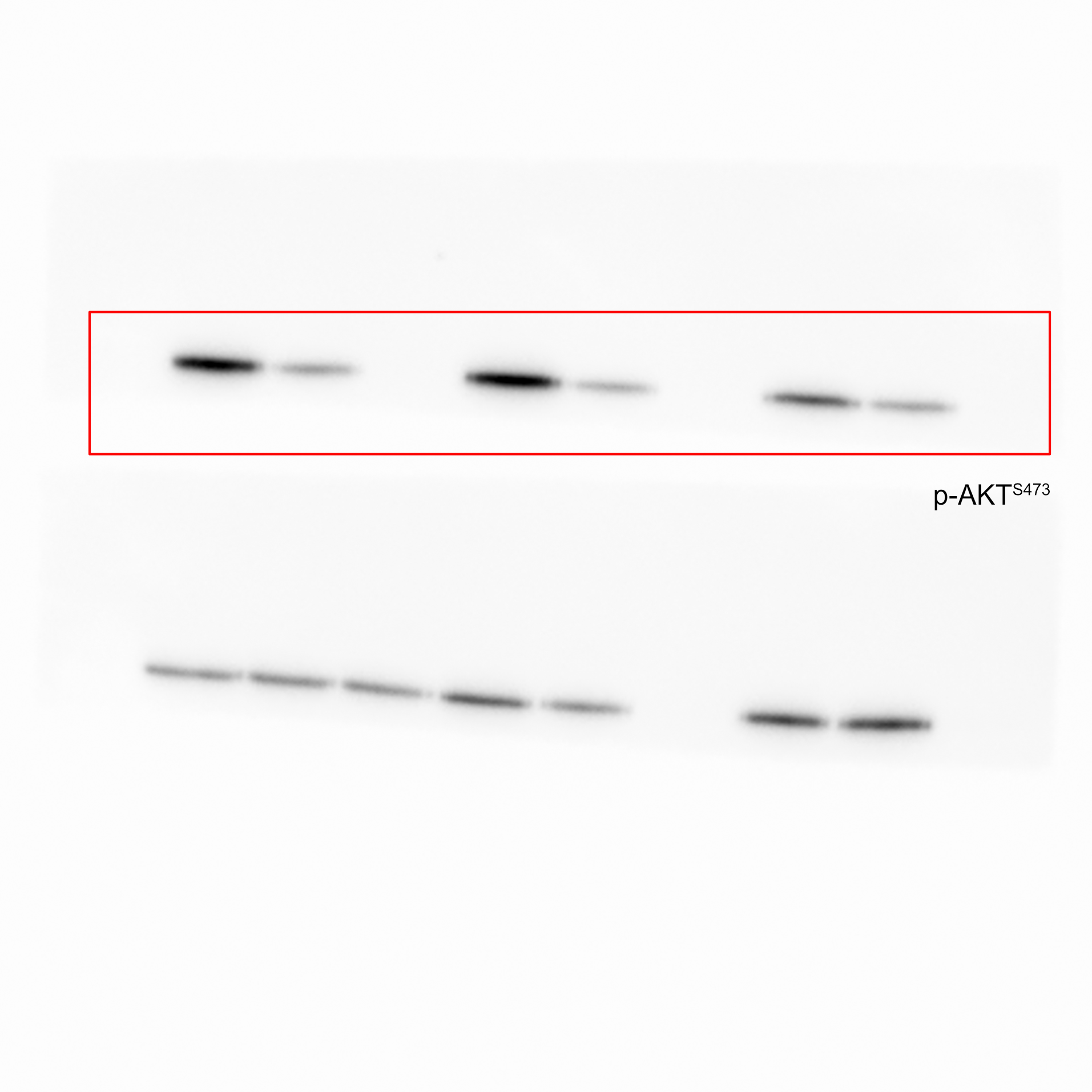

Supplement: Supplementary file 8 — Source data Fig. 5 [file 44318_2025_608_MOESM8_ESM.zip › Figure 5/5C/western pAKTs473.tiff]

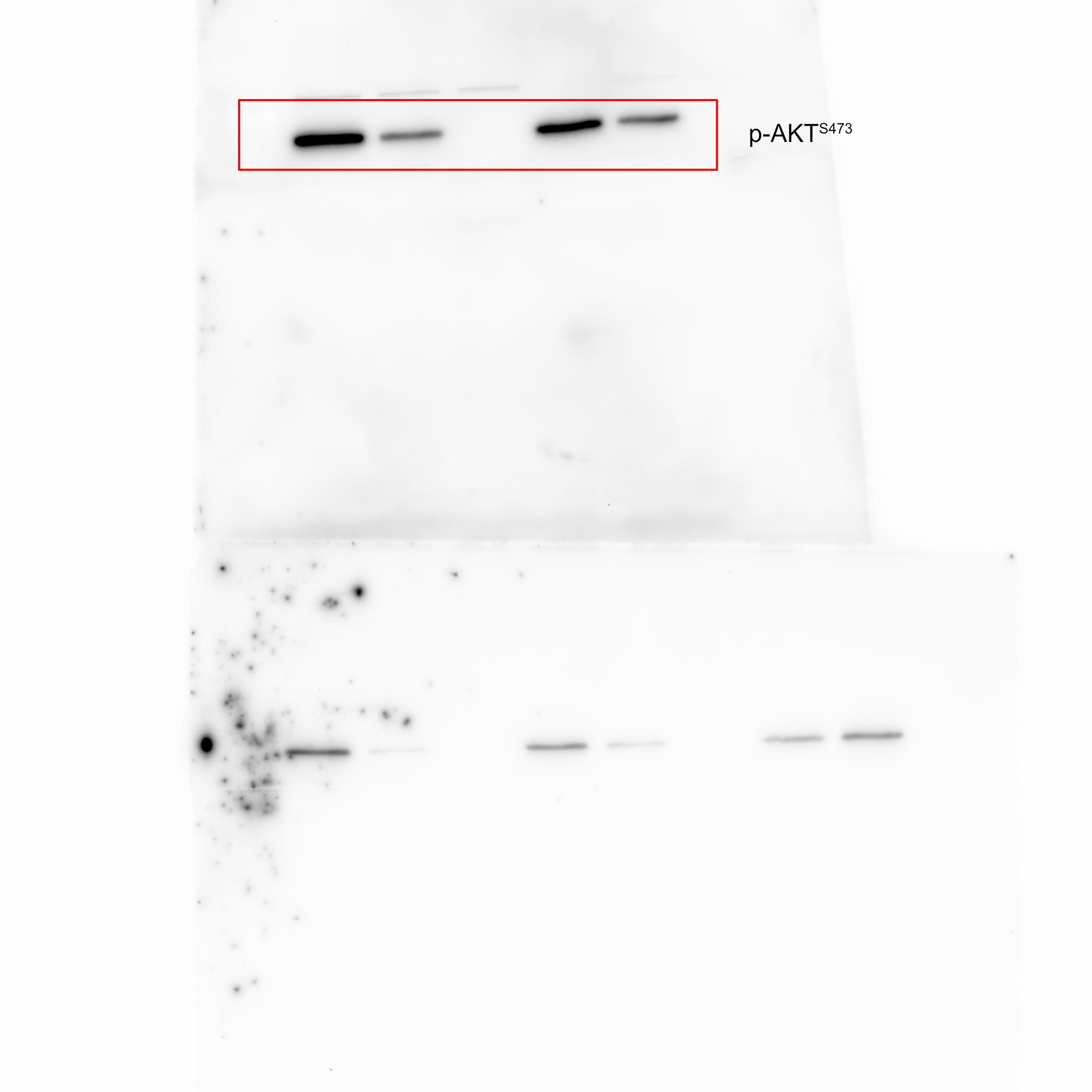

Supplement: Supplementary file 8 — Source data Fig. 5 [file 44318_2025_608_MOESM8_ESM.zip › Figure 5/5C/western pAKTs473_2.tiff]

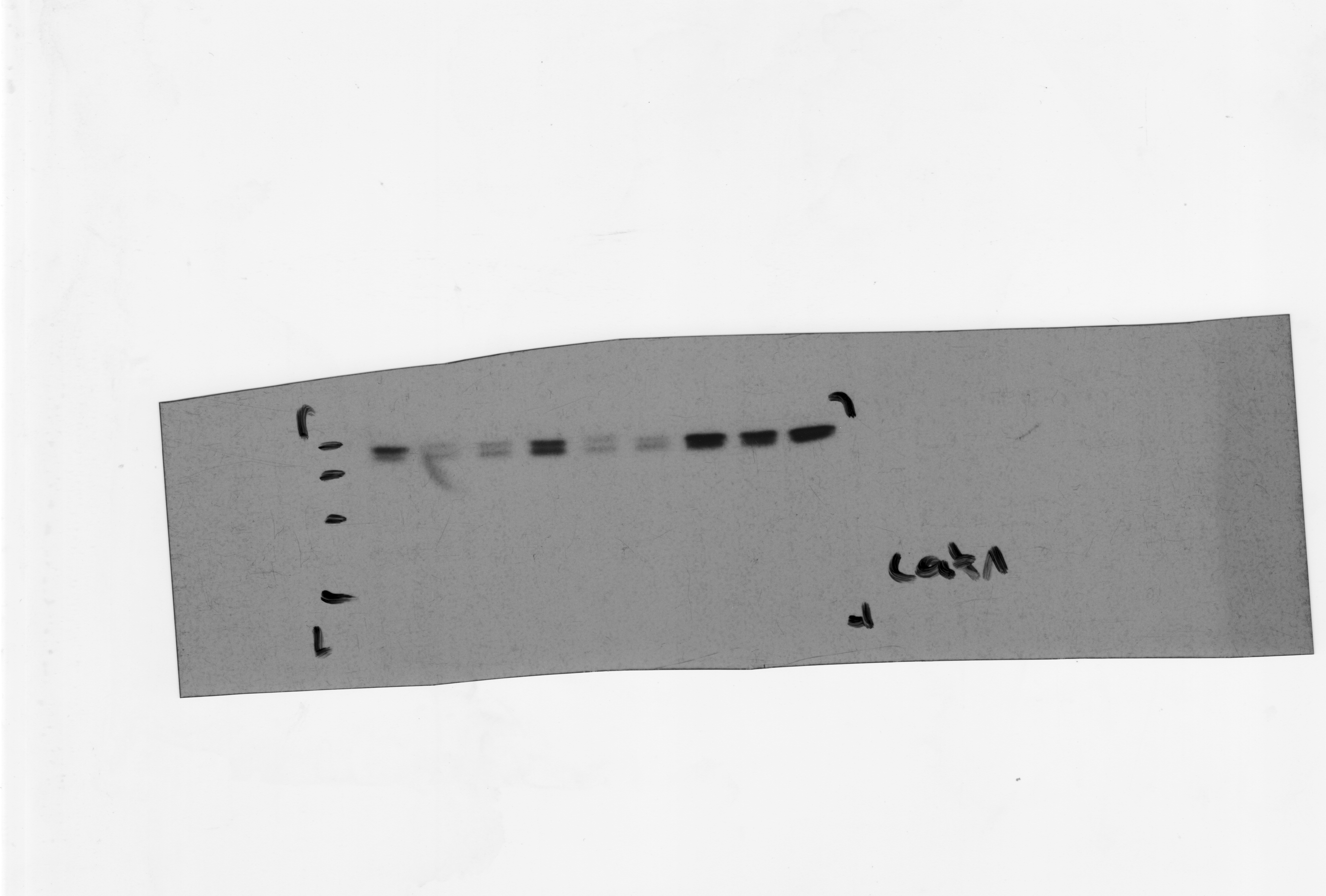

Supplement: Supplementary file 8 — Source data Fig. 5 [file 44318_2025_608_MOESM8_ESM.zip › Figure 5/5C/western SLC7A5.TIF]

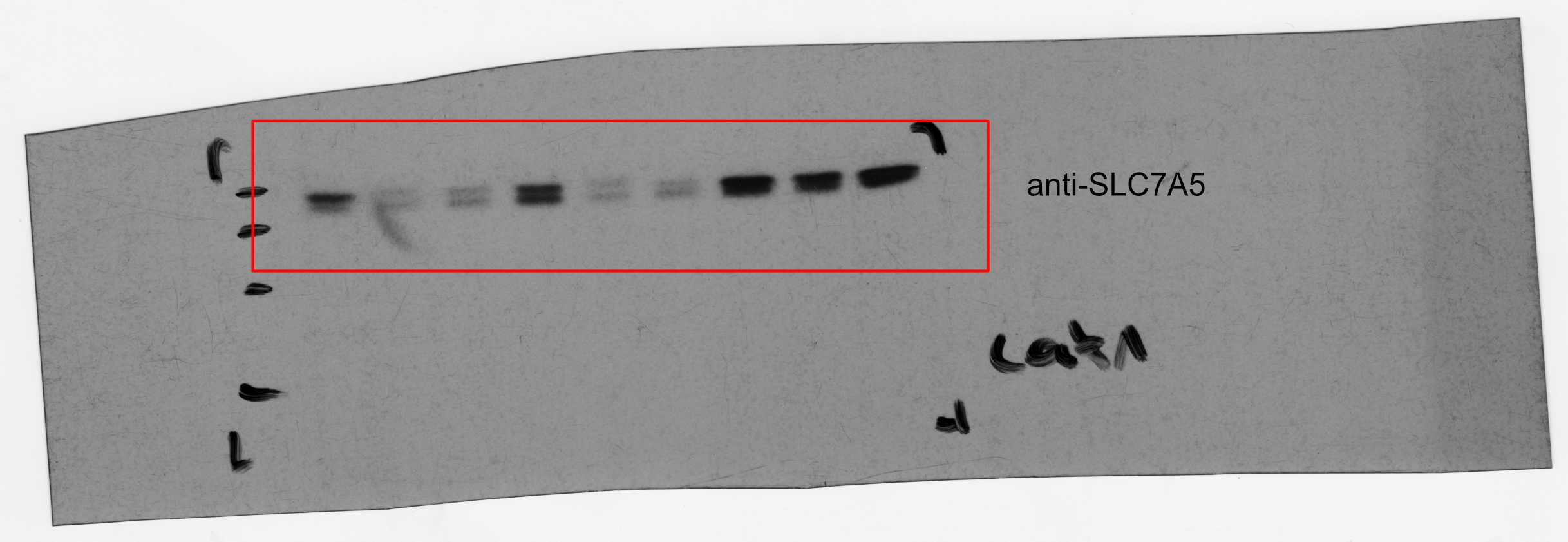

Supplement: Supplementary file 8 — Source data Fig. 5 [file 44318_2025_608_MOESM8_ESM.zip › Figure 5/5C/western SLC7A5.tiff]

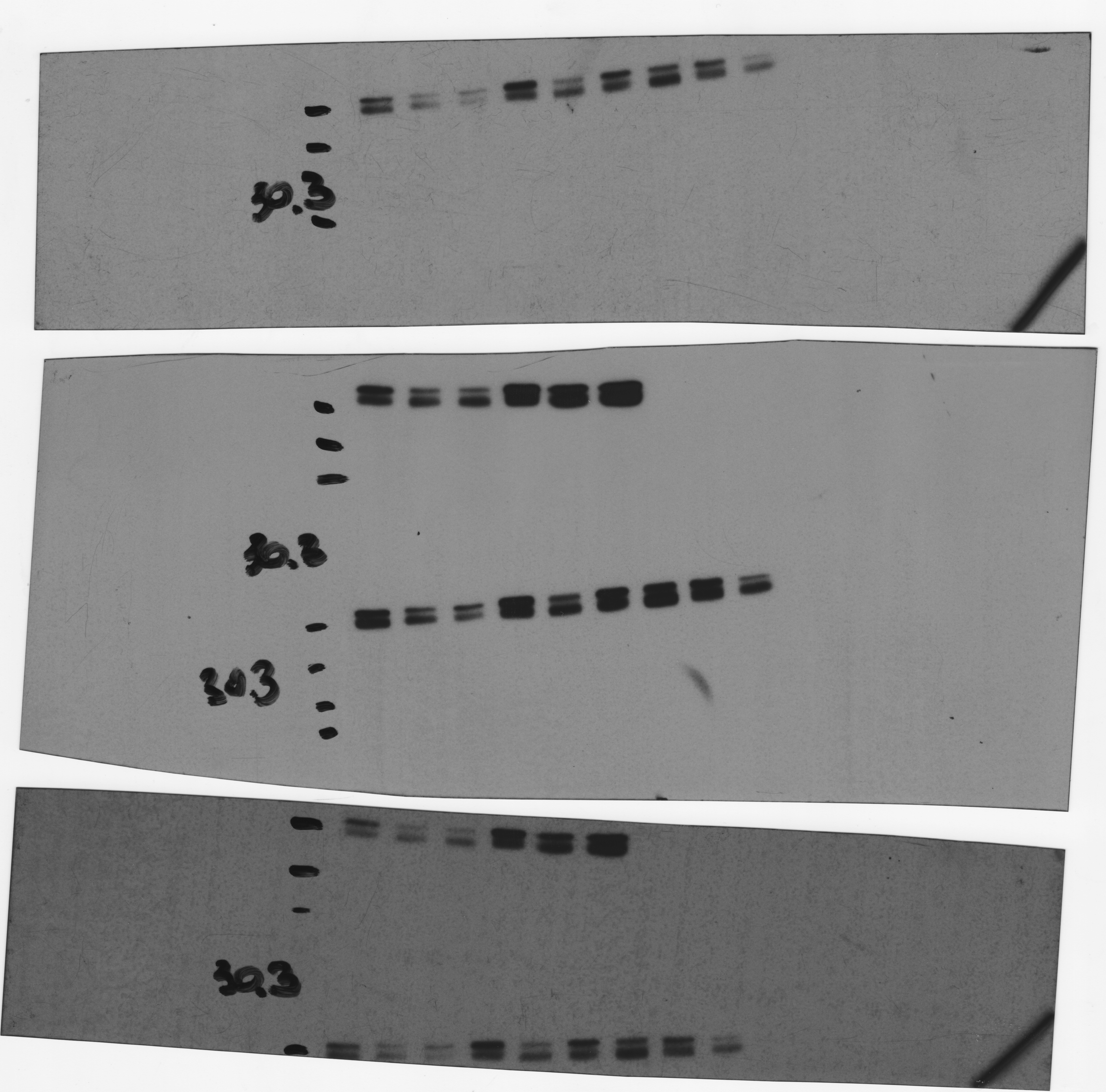

Supplement: Supplementary file 8 — Source data Fig. 5 [file 44318_2025_608_MOESM8_ESM.zip › Figure 5/5C/western SLC7A5_2.TIF]

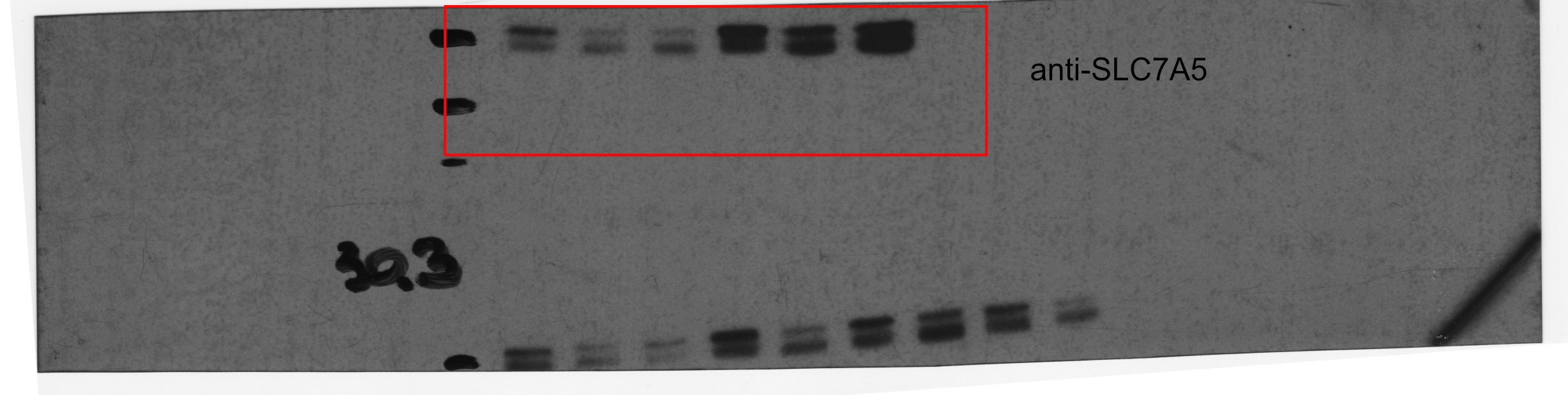

Supplement: Supplementary file 8 — Source data Fig. 5 [file 44318_2025_608_MOESM8_ESM.zip › Figure 5/5C/western SLC7A5_2.tiff]

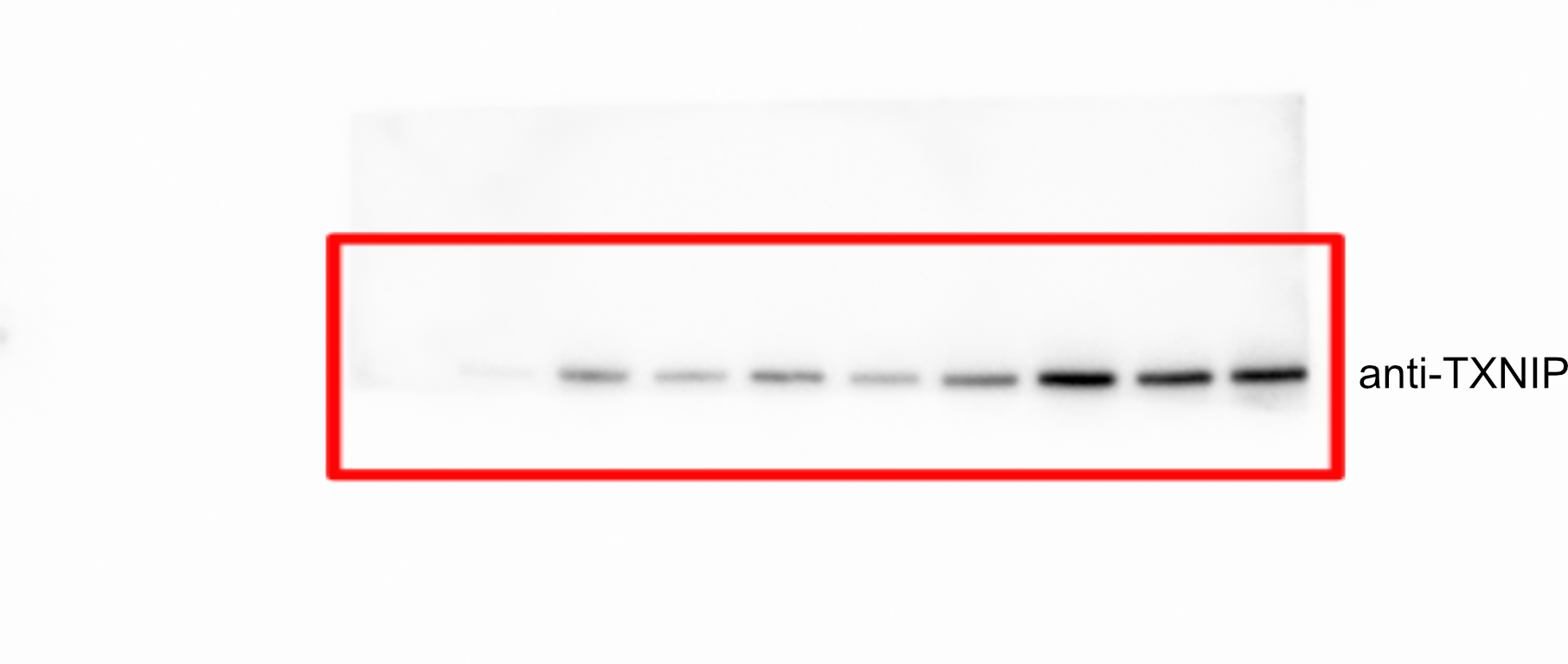

Supplement: Supplementary file 8 — Source data Fig. 5 [file 44318_2025_608_MOESM8_ESM.zip › Figure 5/5C/western TXNIP.tiff]

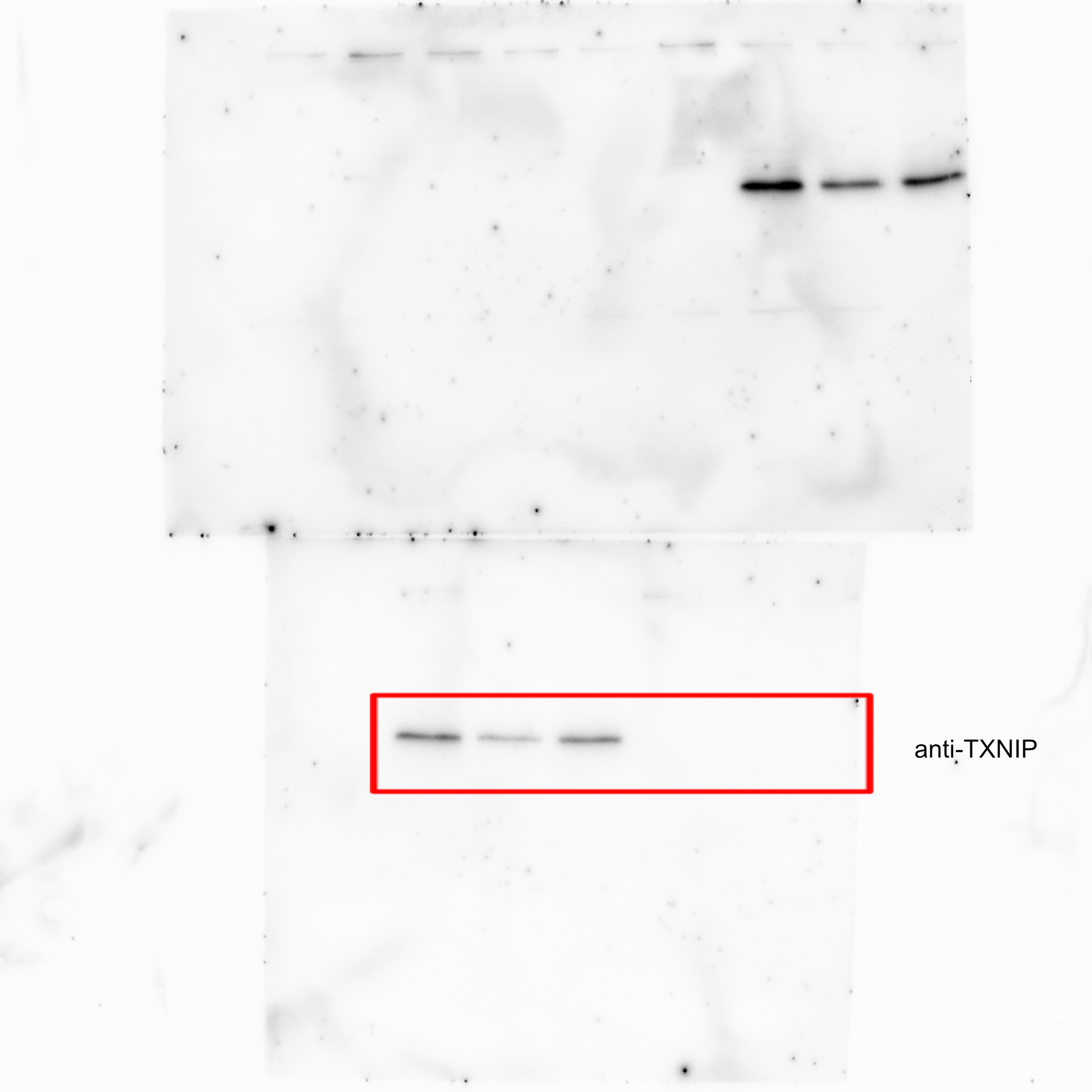

Supplement: Supplementary file 8 — Source data Fig. 5 [file 44318_2025_608_MOESM8_ESM.zip › Figure 5/5C/western TXNIP_2.tiff]

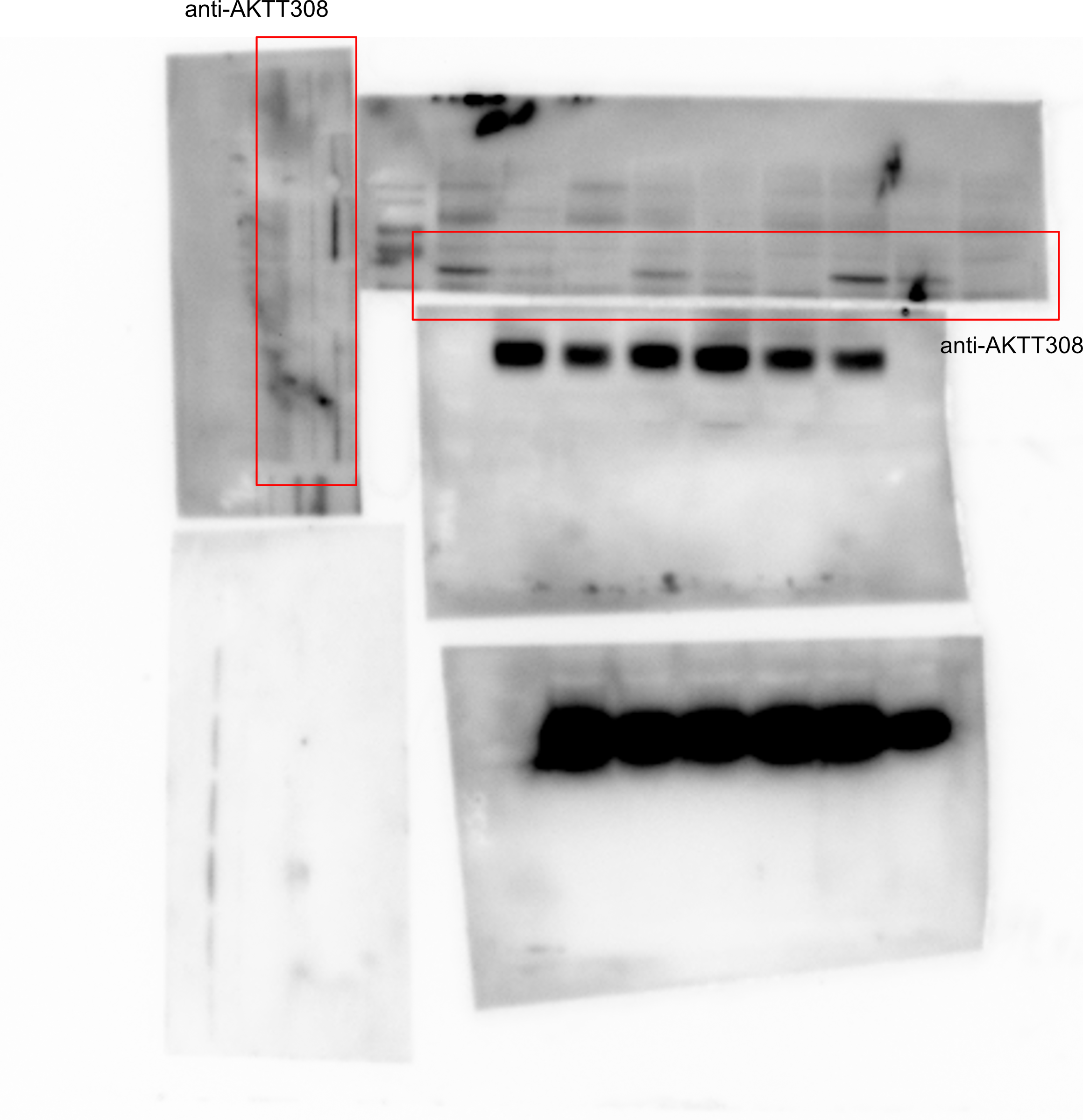

Supplement: Supplementary file 8 — Source data Fig. 5 [file 44318_2025_608_MOESM8_ESM.zip › Figure 5/5C/westernpAKTt308.tiff]

Figure 6C

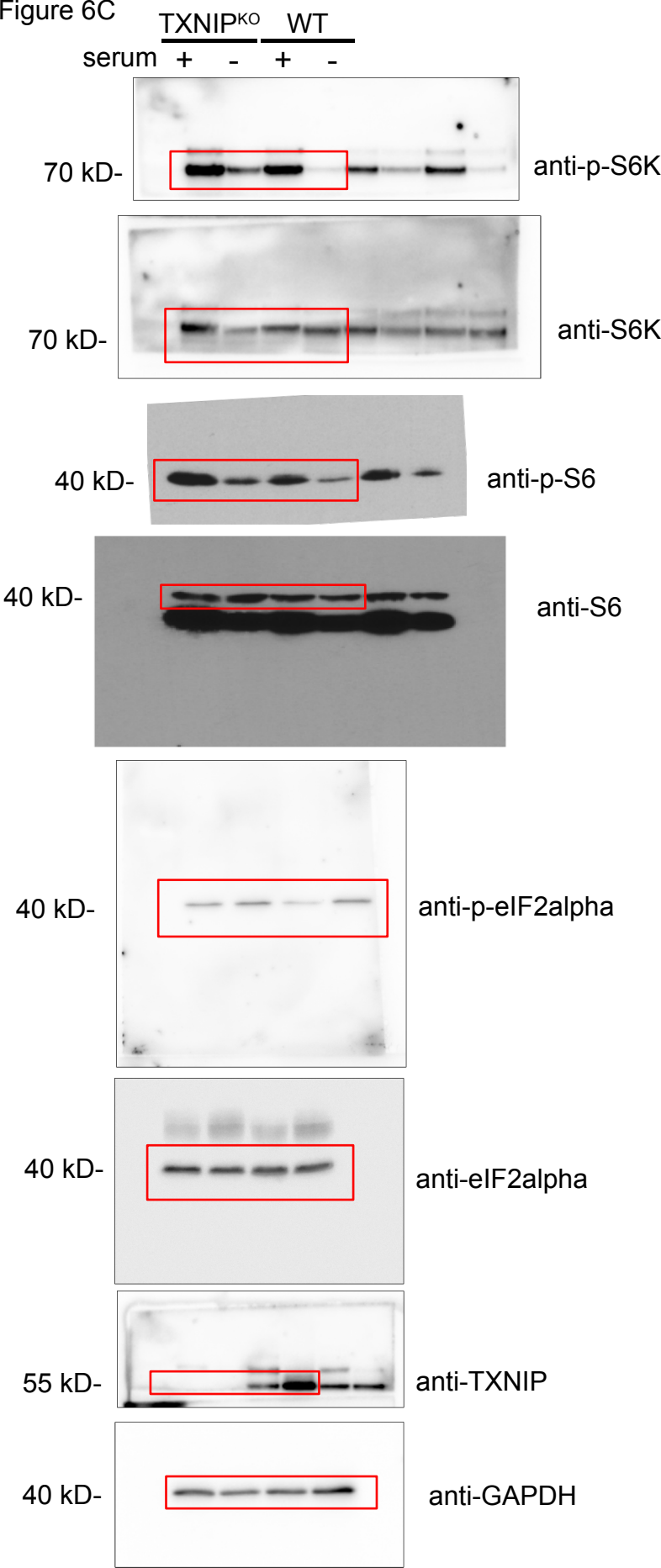

Supplement: Supplementary file 9 — Source data Fig. 6 [file 44318_2025_608_MOESM9_ESM.zip › Figure 6/6C/Figure 6C.pdf]

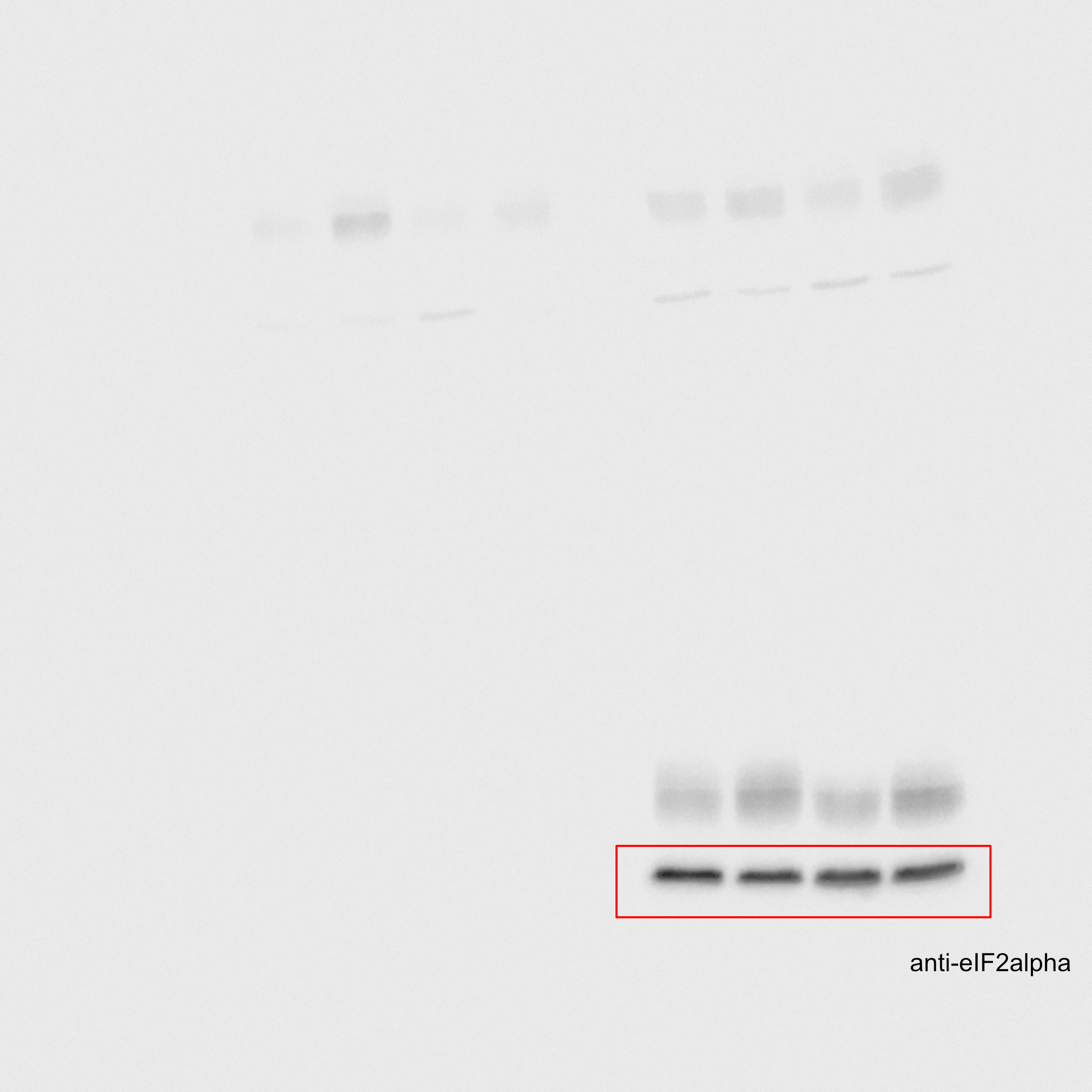

Supplement: Supplementary file 9 — Source data Fig. 6 [file 44318_2025_608_MOESM9_ESM.zip › Figure 6/6C/Western EIF2alpha.tiff]

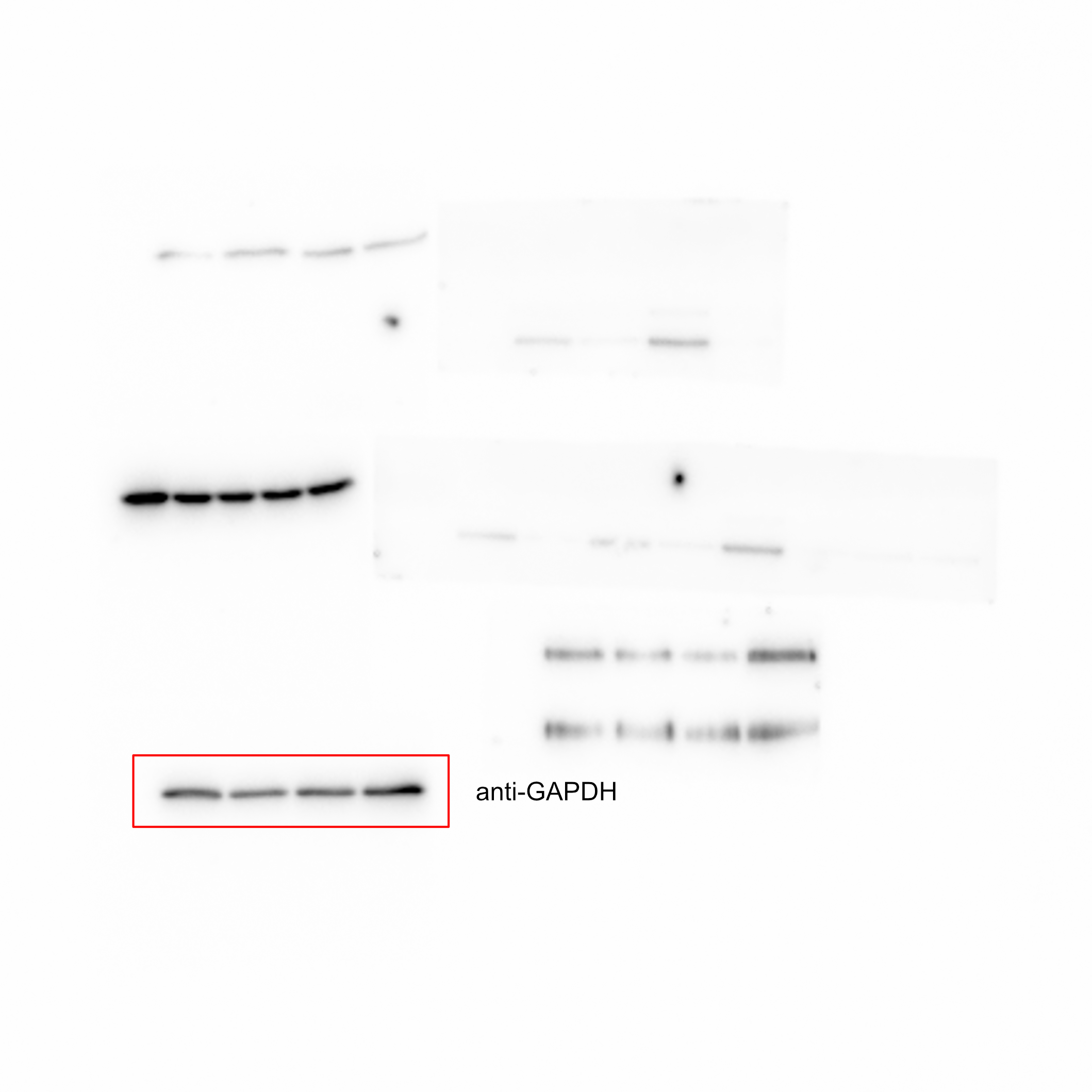

Supplement: Supplementary file 9 — Source data Fig. 6 [file 44318_2025_608_MOESM9_ESM.zip › Figure 6/6C/Western GAPDH.tiff]

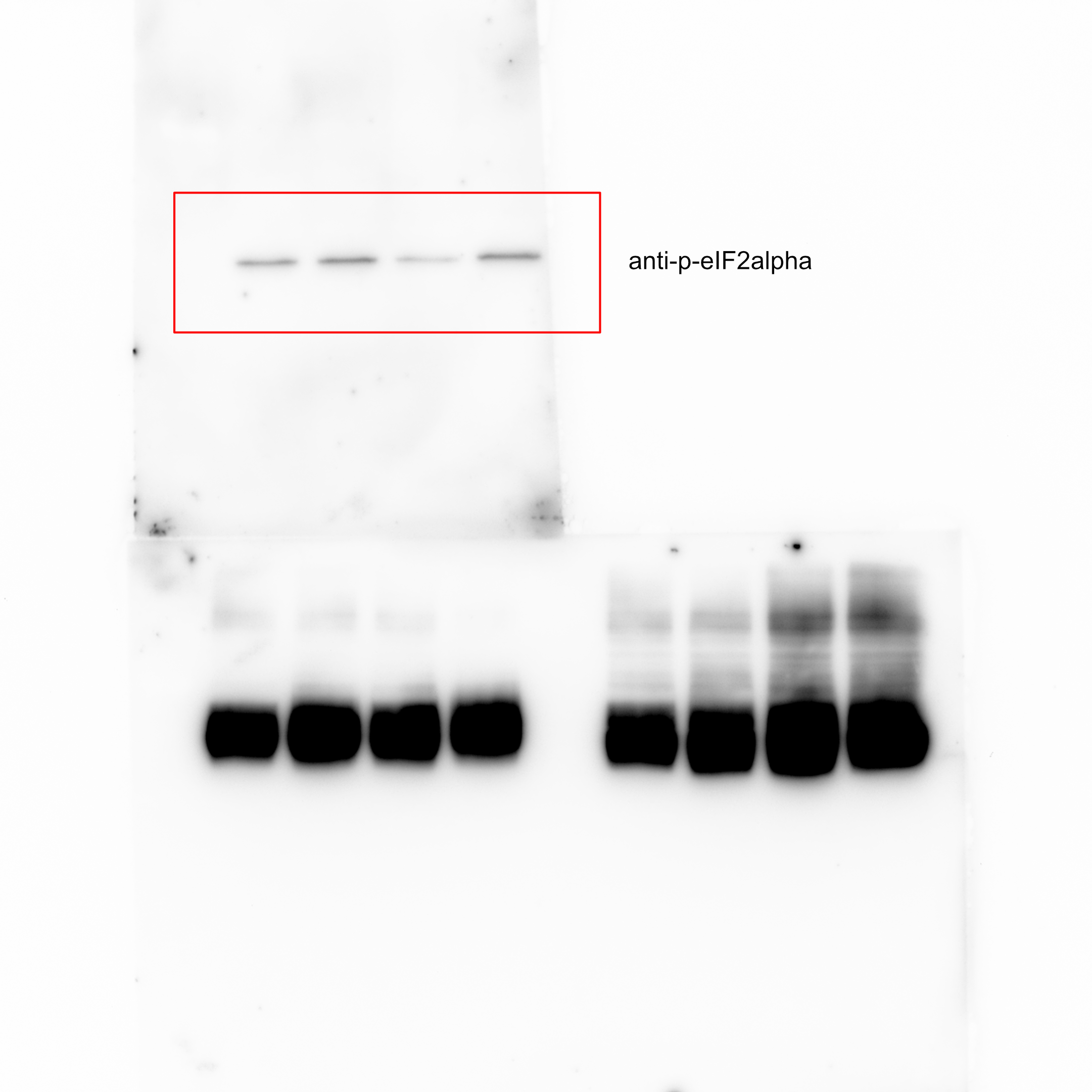

Supplement: Supplementary file 9 — Source data Fig. 6 [file 44318_2025_608_MOESM9_ESM.zip › Figure 6/6C/western pEIF2alpha.tiff]

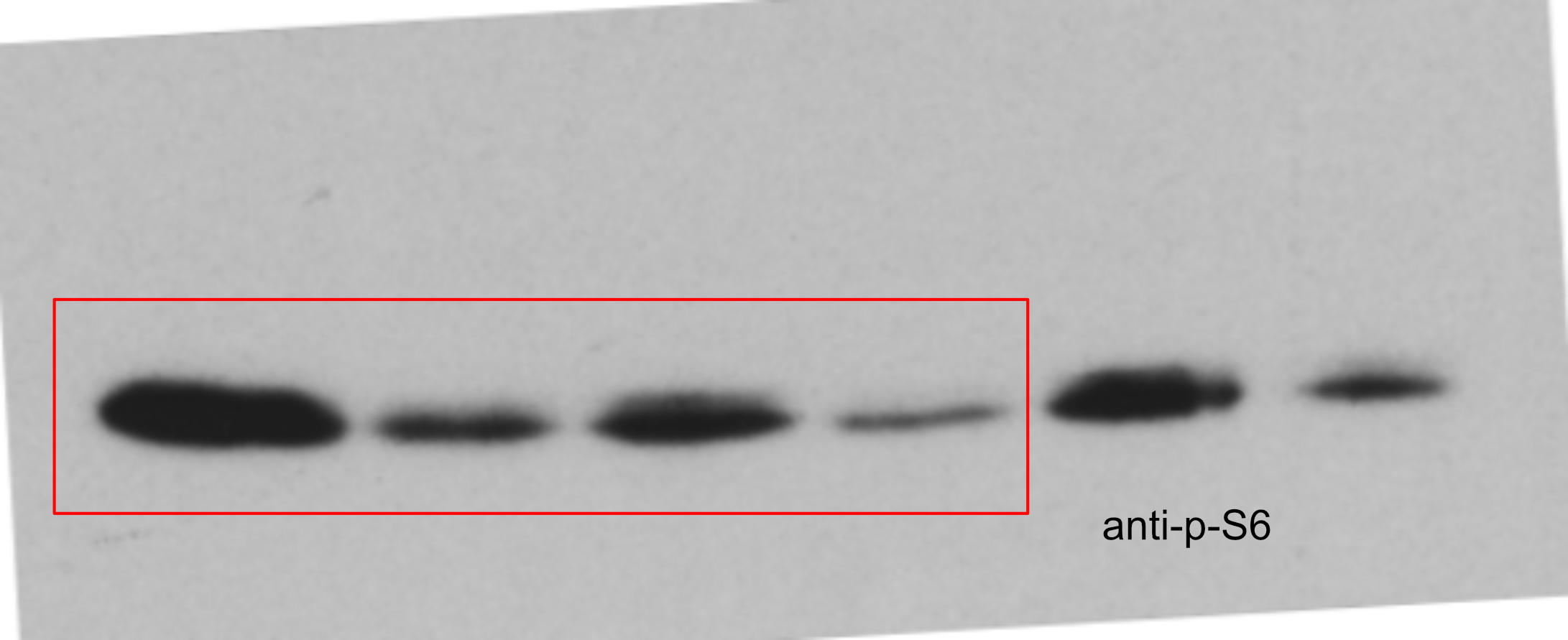

Supplement: Supplementary file 9 — Source data Fig. 6 [file 44318_2025_608_MOESM9_ESM.zip › Figure 6/6C/western pS6.tiff]

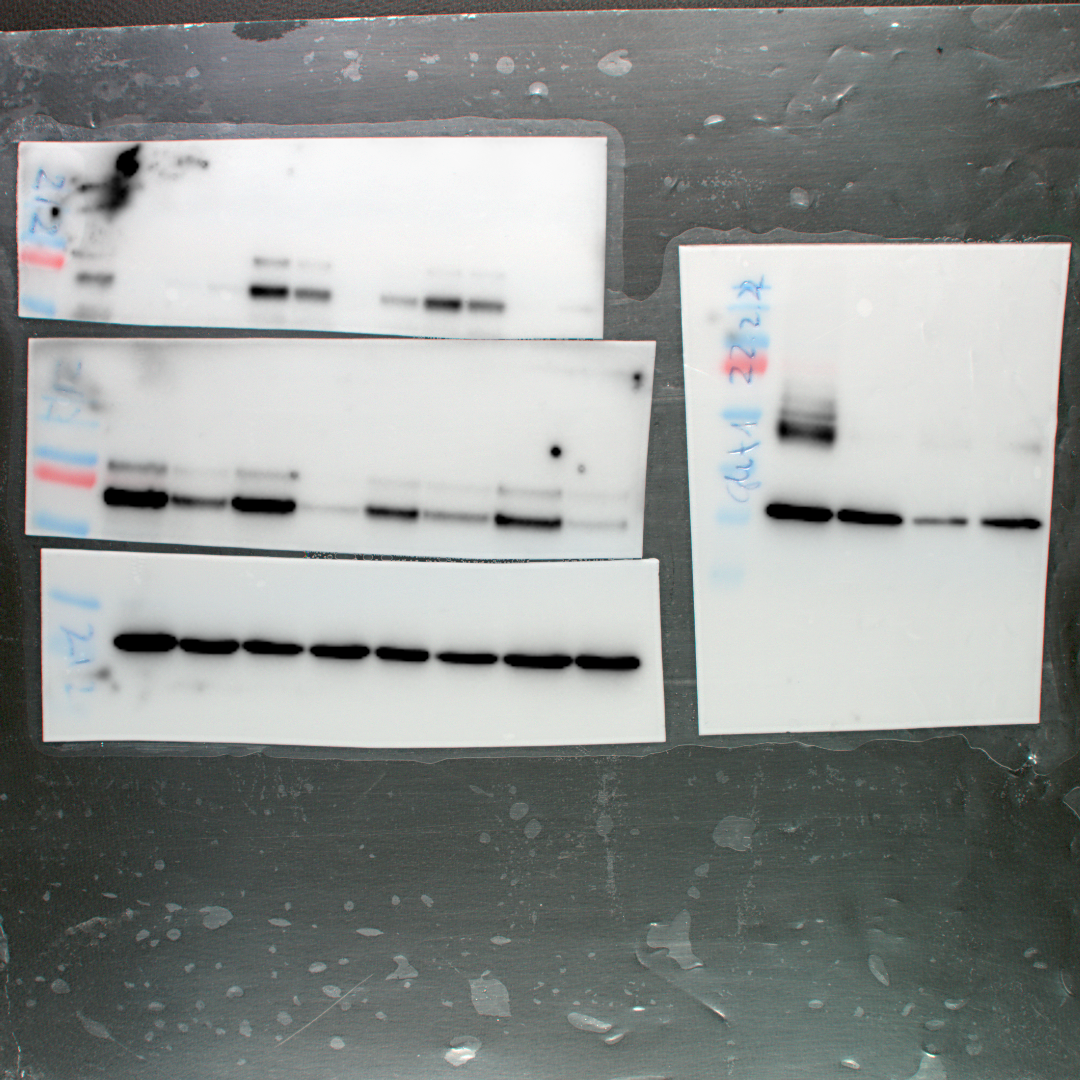

Supplement: Supplementary file 9 — Source data Fig. 6 [file 44318_2025_608_MOESM9_ESM.zip › Figure 6/6C/Western pS6K.Tif]

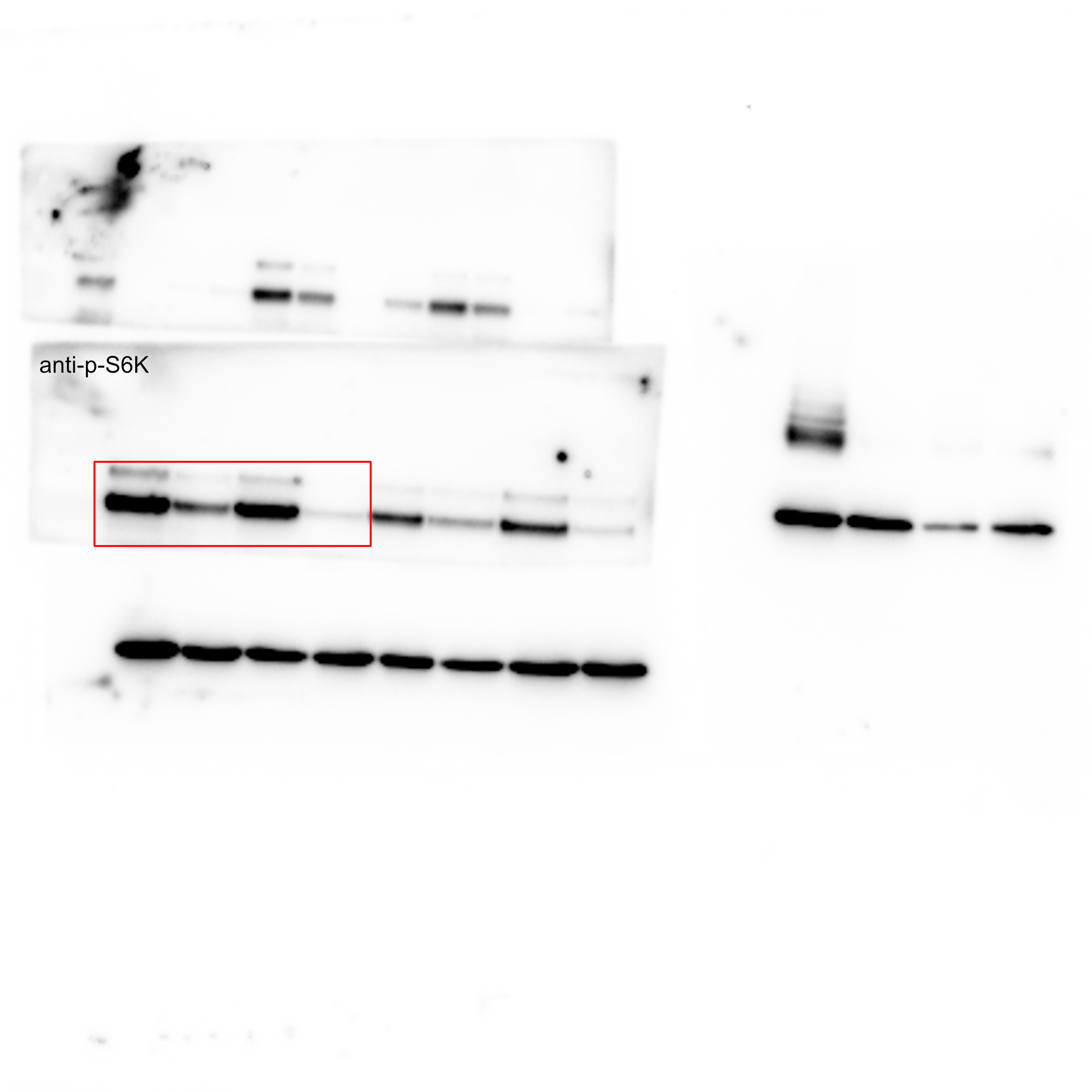

Supplement: Supplementary file 9 — Source data Fig. 6 [file 44318_2025_608_MOESM9_ESM.zip › Figure 6/6C/western p-S6K.tiff]

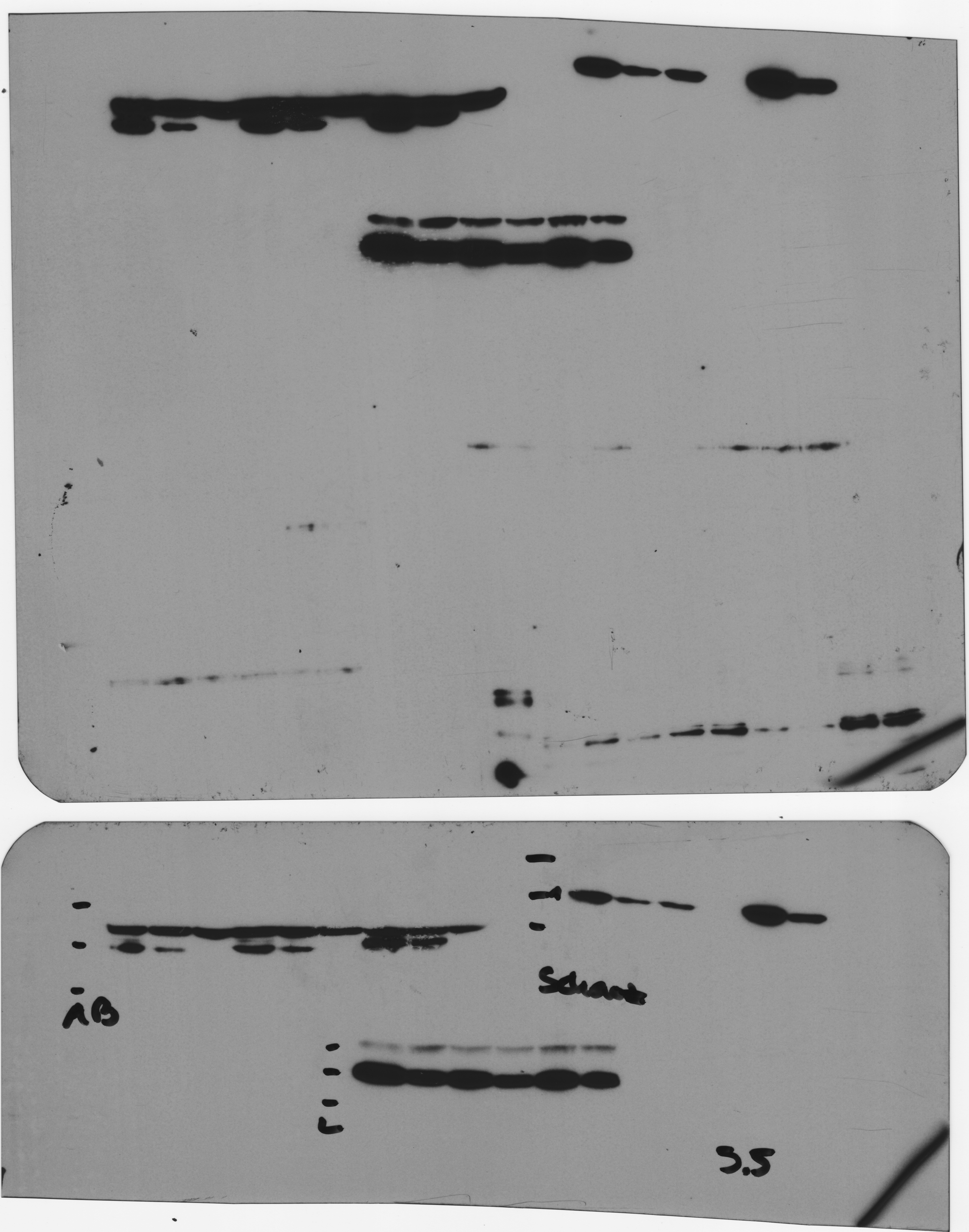

Supplement: Supplementary file 9 — Source data Fig. 6 [file 44318_2025_608_MOESM9_ESM.zip › Figure 6/6C/Western S6.TIF]

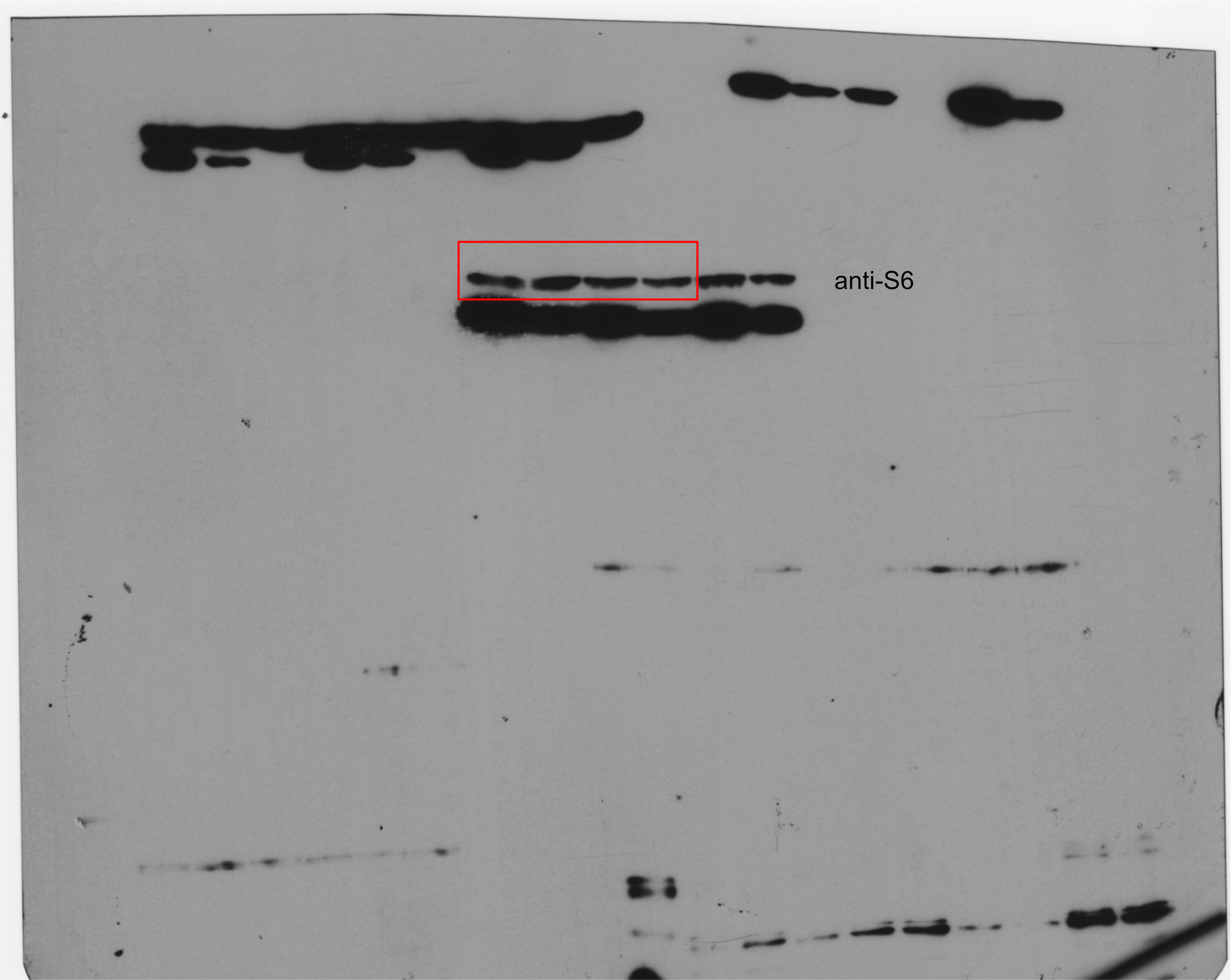

Supplement: Supplementary file 9 — Source data Fig. 6 [file 44318_2025_608_MOESM9_ESM.zip › Figure 6/6C/Western S6.tiff]

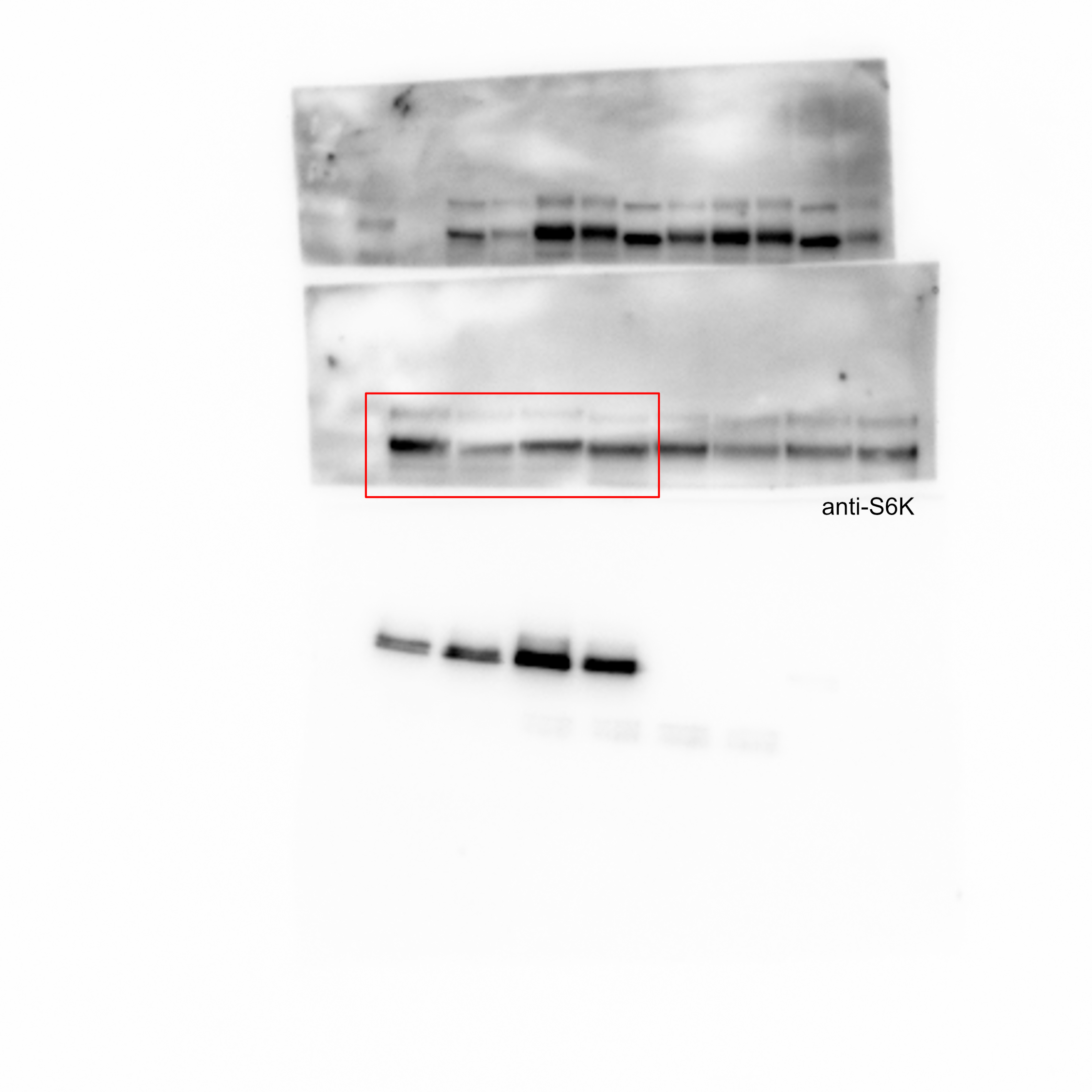

Supplement: Supplementary file 9 — Source data Fig. 6 [file 44318_2025_608_MOESM9_ESM.zip › Figure 6/6C/Western S6K.tiff]

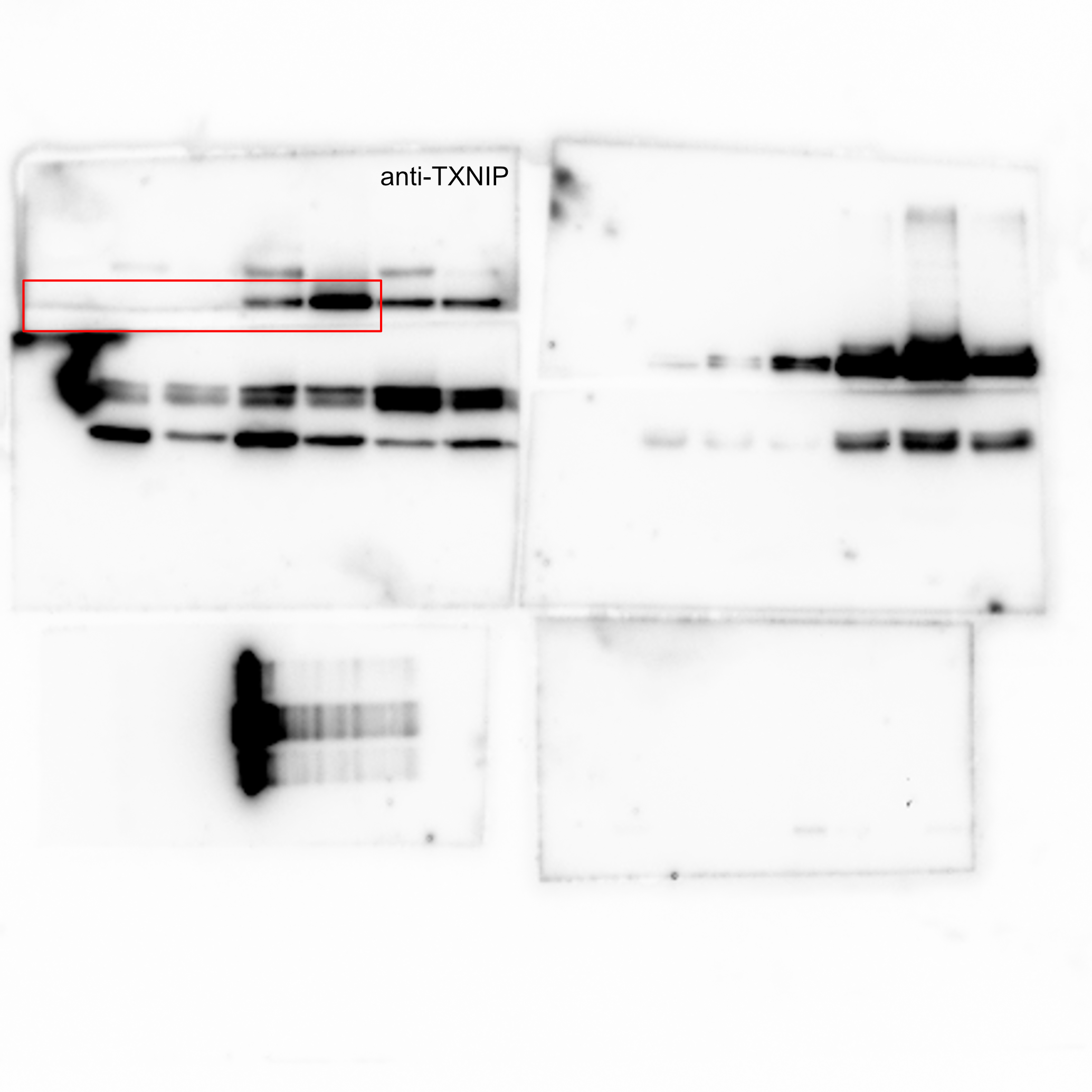

Supplement: Supplementary file 9 — Source data Fig. 6 [file 44318_2025_608_MOESM9_ESM.zip › Figure 6/6C/western TXNIP.tiff]

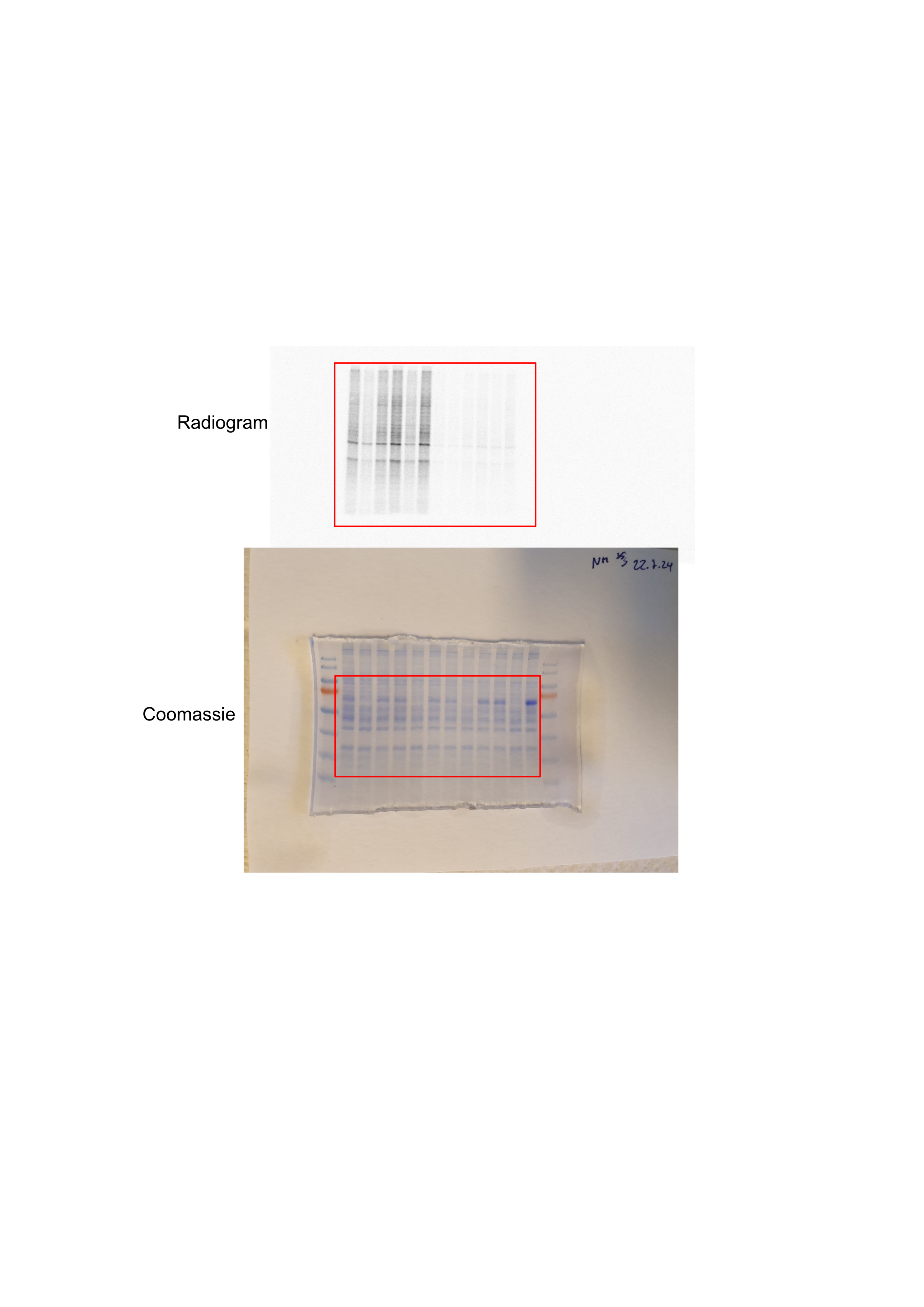

Supplement: Supplementary file 9 — Source data Fig. 6 [file 44318_2025_608_MOESM9_ESM.zip › Figure 6/6G/Figure6G.tiff]
