# Supplementary material for: TXNIP mediates LAT1/SLC7A5 endocytosis to limit amino acid uptake in cells entering quiescence
Source: EMBO J. 2025 Oct 20;44(23):7119–53. doi: 10.1038/s44318-025-00608-9 (PMC12669767; doi:10.1038/s44318-025-00608-9)
Supplement: Supplementary file 5 — Source data Fig. 2 [file 44318_2025_608_MOESM5_ESM.zip › Figure 2/2A/Figure 2A.pdf]

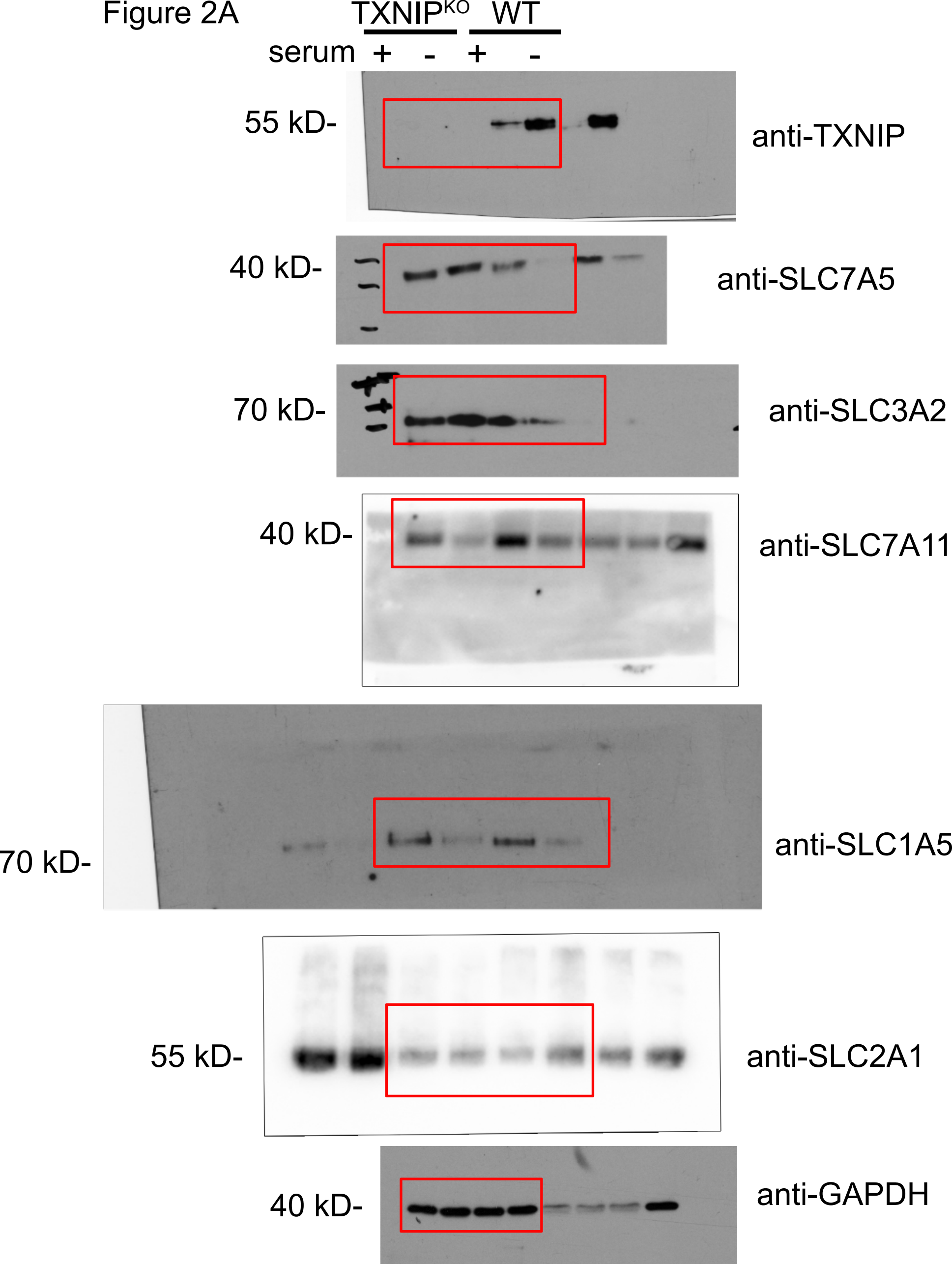

Figure 2A
TXNIP^KO WT
serum + - + -
55 kD-
anti-TXNIP
40 kD-
anti-SLC7A5
70 kD-
anti-SLC3A2
40 kD-
anti-SLC7A11
70 kD-
anti-SLC1A5
55 kD-
anti-SLC2A1
40 kD-
anti-GAPDH
