# Supplementary material for: TXNIP mediates LAT1/SLC7A5 endocytosis to limit amino acid uptake in cells entering quiescence
Source: EMBO J. 2025 Oct 20;44(23):7119–53. doi: 10.1038/s44318-025-00608-9 (PMC12669767; doi:10.1038/s44318-025-00608-9)
Supplement: Supplementary file 11 — Expanded View Figures [file 44318_2025_608_MOESM11_ESM.pdf]

Expanded View Figures

Figure EV1. Cells entering quiescence selectively downregulate amino acid transporters.

(**A**) WT cells were grown in growth medium (+ serum) or serum starved for 2 h, 4 h, 6 h and 24 h (− serum). Total cell lysates were analyzed by SDS-PAGE and WB with the indicated antibodies. WB quantification of SLC7A5, TAX1BP1 normalized to Vinculin and NBR1, NDP52, LC3 normalized to Ponceau ($n = 3$, SEM, paired *t* test). (**B**, **E**) Indirect IF of PFA fixed cells stained for SLC1A5 (yellow) LAMP1 (magenta) and DAPI (cyan) were analyzed by confocal microscopy. The merged images show a single plane of a Z-stack. Incubation with 12,5 µM chloroquine (CQ) in absence of serum for 14 h (− serum, + CQ). Scale bar=10 µm) (**C**) Quantification of SLC3A2 IF signal at the plasma membrane under serum-supplemented (+ serum) and starved (-serum) conditions with chloroquine (+ CQ) or without it. Box plots represent the median (centre line) and the interquartile range (25th to 75th percentile box). The whiskers show the minimum and maximum values. ($n = 35$, one way anova, Tukey's multiple comparison test, +serum –CQ vs –serum –CQ: $P = 5.7E\text{-}14$, +serum –CQ vs –serum +CQ: $P = 8.5E\text{-}13$). (**D**) WB quantification of SLC7A5 under serum-supplemented (+ serum) and starved (-serum) conditions starved (-serum) conditions with chloroquine (+ CQ) or without it. Box plots represent the median (centre line) and the interquartile range (25th to 75th percentile box). The whiskers show the minimum and maximum values. ($n = 3$, one way ANOVA, Tukey's multiple comparison test) (**E**) Incubation with 20 µM dynasore (dyna) in absence of serum for 14 h (− serum, + dyna). Scale bar=10 µm. (**F**) Quantification of SLC3A2 IF signal at the plasma membrane under serum-supplemented (+ serum) and starved (-serum) conditions with dynasore (+ dyna) or without it. Box plots represent the median (centre line) and the interquartile range (25th to 75th percentile box). The whiskers show the minimum and maximum values. ($n = 50$, one-way anova, Tukey's multiple comparison test, +serum –dyna vs –serum –dyna: $P = 8.7E\text{-}14$, +serum –dyna vs –serum +dyna: $P = 7.6E\text{-}14$). (**G**) WB quantification of SLC7A5 under serum-supplemented (+ serum) and starved (−serum) conditions in the presence (+ dyna) or absence (−dyna) of dynasore. Box plots represent the median (centre line) and the interquartile range (25th to 75th percentile box). The whiskers show the minimum and maximum values ($n = 3$, one-way ANOVA, Tukey's multiple comparison test). (**H**) Cells were incubated with[14 C]-glutamine for 15 min, washed and lysed. Total cell lysates were analyzed by scintillation counting. Cpm values were normalized to total protein content. Box plots represent the median (centre line) and the interquartile range (25th to 75th percentile box). The whiskers show the minimum and maximum values. (cpm/µg protein, $n = 4$, paired *t* test).

 © The Author(s)

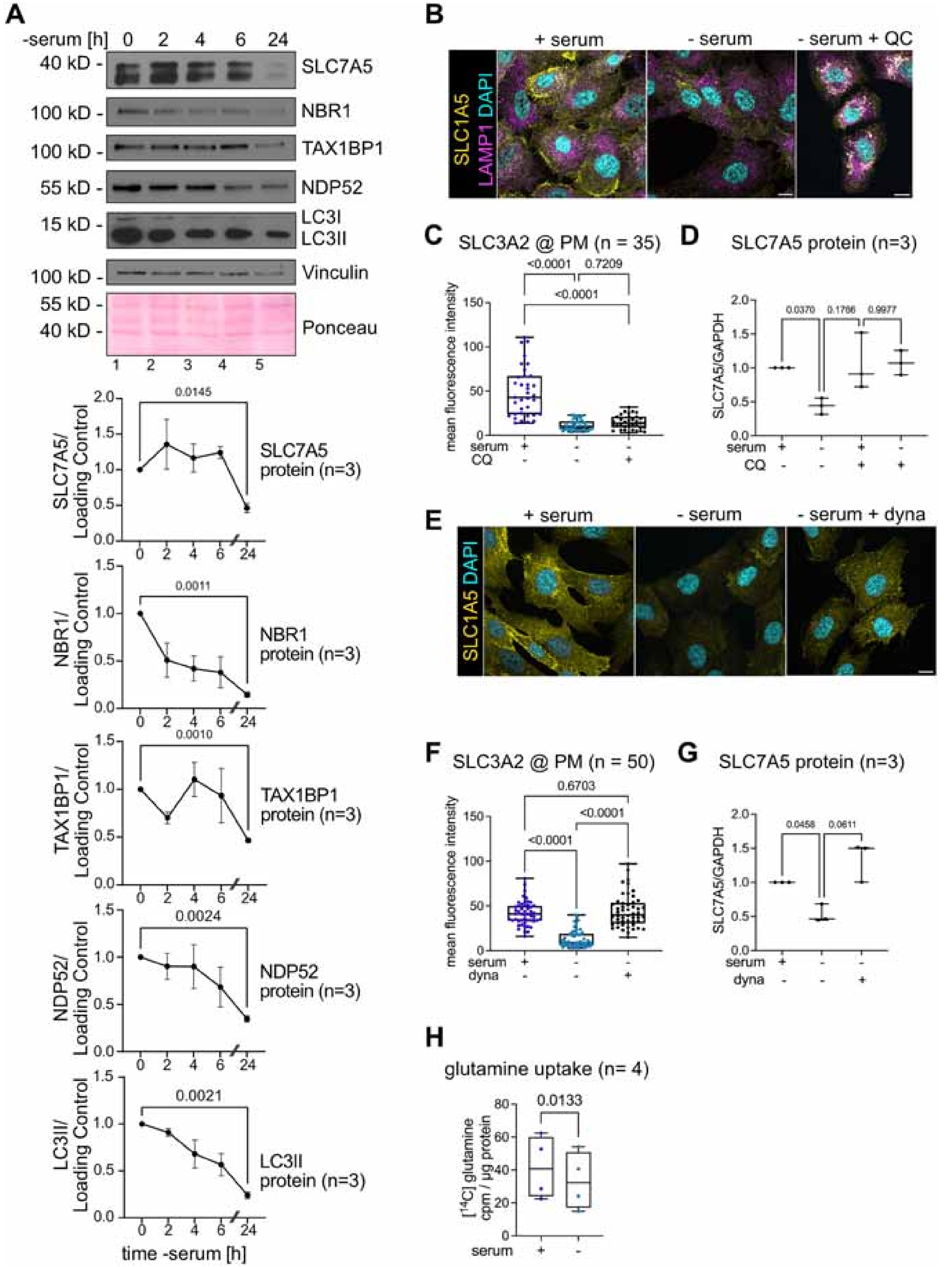
A
-serum [h] 0 2 4 6 24
40 kD - SLC7A5
100 kD - NBR1
100 kD - TAX1BP1
55 kD - NDP52
15 kD - LC3I LC3II
100 kD - Vinculin
55 kD - 40 kD - Ponceau
1 2 3 4 5
SLC7A5/ Loading Control
0.0145
SLC7A5 protein (n=3)
NBR1/ Loading Control
0.0011
NBR1 protein (n=3)
TAX1BP1/ Loading Control
0.0010
TAX1BP1 protein (n=3)
NDP52/ Loading Control
0.0024
NDP52 protein (n=3)
LC3II/ Loading Control
0.0021
LC3II protein (n=3)
time -serum [h]
B
+ serum
- serum
- serum + QC
SLC1A5 LAMP1 DAPI
C
SLC3A2 @ PM (n = 35)
<0.0001 0.7209
<0.0001
mean fluorescence intensity
serum
CQ
D
SLC7A5 protein (n=3)
0.0370 0.1786 0.9977
SLC7A5/GAPDH
serum
CQ
E
+ serum
- serum
- serum + dyna
SLC1A5 DAPI
F
SLC3A2 @ PM (n = 50)
0.6703
<0.0001
<0.0001
mean fluorescence intensity
serum
dyna
G
SLC7A5 protein (n=3)
0.0458 0.0611
SLC7A5/GAPDH
serum
dyna
H
glutamine uptake (n= 4)
0.0133
[14C] glutamine cpm / µg protein
serum

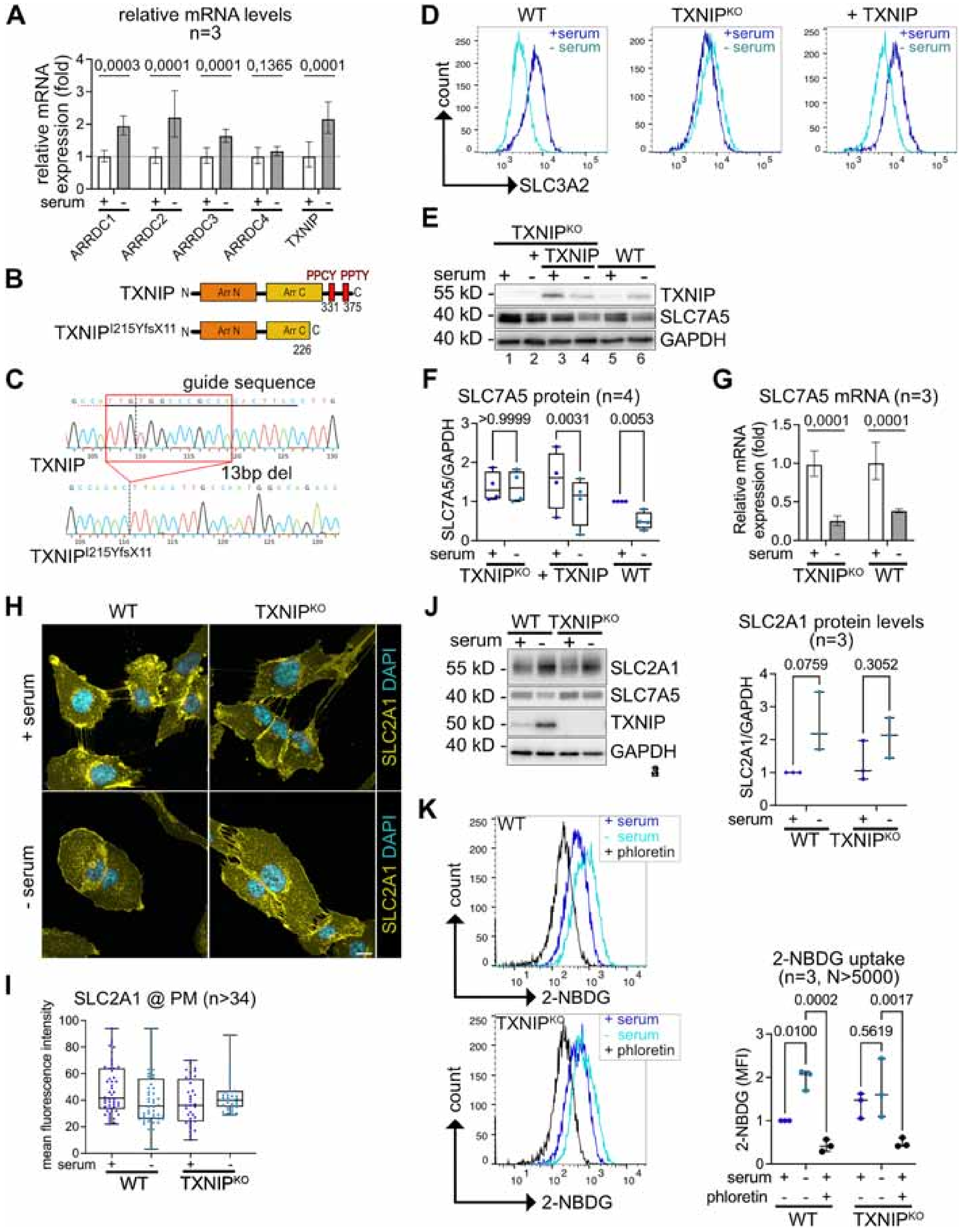
A
relative mRNA levels
n=3
relative mRNA expression (fold)
0,0003 0,0001 0,0001 0,1365 0,0001
serum
ARRDC1 ARRDC2 ARRDC3 ARRDC4 TXNIP
B
TXNIP
PPCY PPTY
Arr N Arr C
331 375
TXNIPI215YfsX11
226
C
guide sequence
TXNIP
13bp del
TXNIPI215YfsX11
D
WT TXNIPKO + TXNIP
+serum
- serum
count
SLC3A2
E
TXNIPKO
+ TXNIP WT
serum
55 kD TXNIP
40 kD SLC7A5
40 kD GAPDH
1 2 3 4 5 6
F
SLC7A5 protein (n=4)
SLC7A5/GAPDH
>0.9999 0.0031 0.0053
serum
TXNIPKO + TXNIP WT
G
SLC7A5 mRNA (n=3)
Relative mRNA expression (fold)
0,0001 0,0001
serum
TXNIPKO WT
H
WT TXNIPKO
+ serum
- serum
SLC2A1 DAPI
I
SLC2A1 @ PM (n>34)
mean fluorescence intensity
serum
WT TXNIPKO
J
WT TXNIPKO
serum
55 kD SLC2A1
40 kD SLC7A5
50 kD TXNIP
40 kD GAPDH
SLC2A1 protein levels
(n=3)
SLC2A1/GAPDH
0.0759 0.3052
serum
WT TXNIPKO
K
WT
TXNIPKO
+ serum
- serum
+ phloretin
count
2-NBDG
2-NBDG uptake
(n=3, N>5000)
2-NBDG (MFI)
0.0002 0.0017
0.0100 0.5619
serum
phloretin
WT TXNIPKO

 © The Author(s)

Figure EV2. TXNIP is required for the endocytosis of SLC7A5.

qPCR analysis of (**A**) α-arrestins mRNA in growing (+ serum) or serum starved cells (− serum). Expression levels were normalized to the housekeeping gene *RPLP0* ($n = 3$, paired *t* test). (**B**) Schematic presentation of the mutation introduced into TXNIP by gene editing. (**C**) Representative chromatogram of Sanger sequencing from the genome amplicon of WT TXNIP and gene edited TXNIP cells. The guide RNA and the protospacer adjacent motif (PAM) sequence are indicated. The vertical lane shows the Cas9 editing site causing a 13 base pair deletion in *TXNIP* (red box). Image was generated by the ICE Analysis online tool (Synthego, v2.0; Synthego, USA). (**D**) Representative histograms of SLC3A2 cell surface FACS from WT, $TXNIP^{KO}$ and $TXNIP^{KO}$ reconstituted with TXNIP cells. (**E**) WT, $TXNIP^{KO}$ and $TXNIP^{KO}$ reconstituted with TXNIP were grown in growth medium (+ serum) or serum starved for 24 h (− serum). Total cell lysates were analysed by SDS-PAGE and WB with the indicated antibodies. (**F**) WB quantification of SLC7A5 protein levels, normalized to GAPDH. Box plots represent the median (centre line) and the interquartile range (25th to 75th percentile box). The whiskers show the minimum and maximum values. ($n = 4$, two-way ANOVA, Sidak's multiple comparisons test). (**G**) qPCR analysis of SLC7A5 mRNA in growing (+ serum) or serum starved cells (- serum). Expression levels were normalized to the housekeeping gene *RPLP0*. The bar charts show mean values ($n = 3$, paired *t* test). (**H**) Indirect of PFA fixed WT and $TXNIP^{KO}$ cells stained for SLC2A1 (yellow) and DAPI (cyan) was analysed by confocal microscopy. The merged images show a single plane of a Z-stack. Scale bar = 10 μm. (**I**) Quantification of SLC2A1 IF signal at the PM. Box plots represent the median (centre line) and the interquartile range (25th to 75th percentile box). The whiskers show the minimum and maximum values. ($n > 34$). (**J**) Total cell lysates were analysed by SDS-PAGE and WB with the indicated antibodies. For WB quantification, SLC2A1 protein levels were normalized to GAPDH. Box plots represent the median (centre line) and the interquartile range (25th to 75th percentile box). The whiskers show the minimum and maximum values. ($n = 3$, two-way ANOVA, Sidak's multiple comparisons test). (**K**) WT and $TXNIP^{KO}$ cells were assessed for glucose uptake by incorporation of 100 μM 2-NBDG for 45 min. Simultaneously, cells were treated with 1 mM phloretin. The fluorescence intensity was analysed by FACS. Box plots represent the median (centre line) and the interquartile range (25th to 75th percentile box). The whiskers show the minimum and maximum values ($n = 3$, $N > 5000$ cells, two-way ANOVA, Sidak's multiple comparisons test).

© The Author(s)

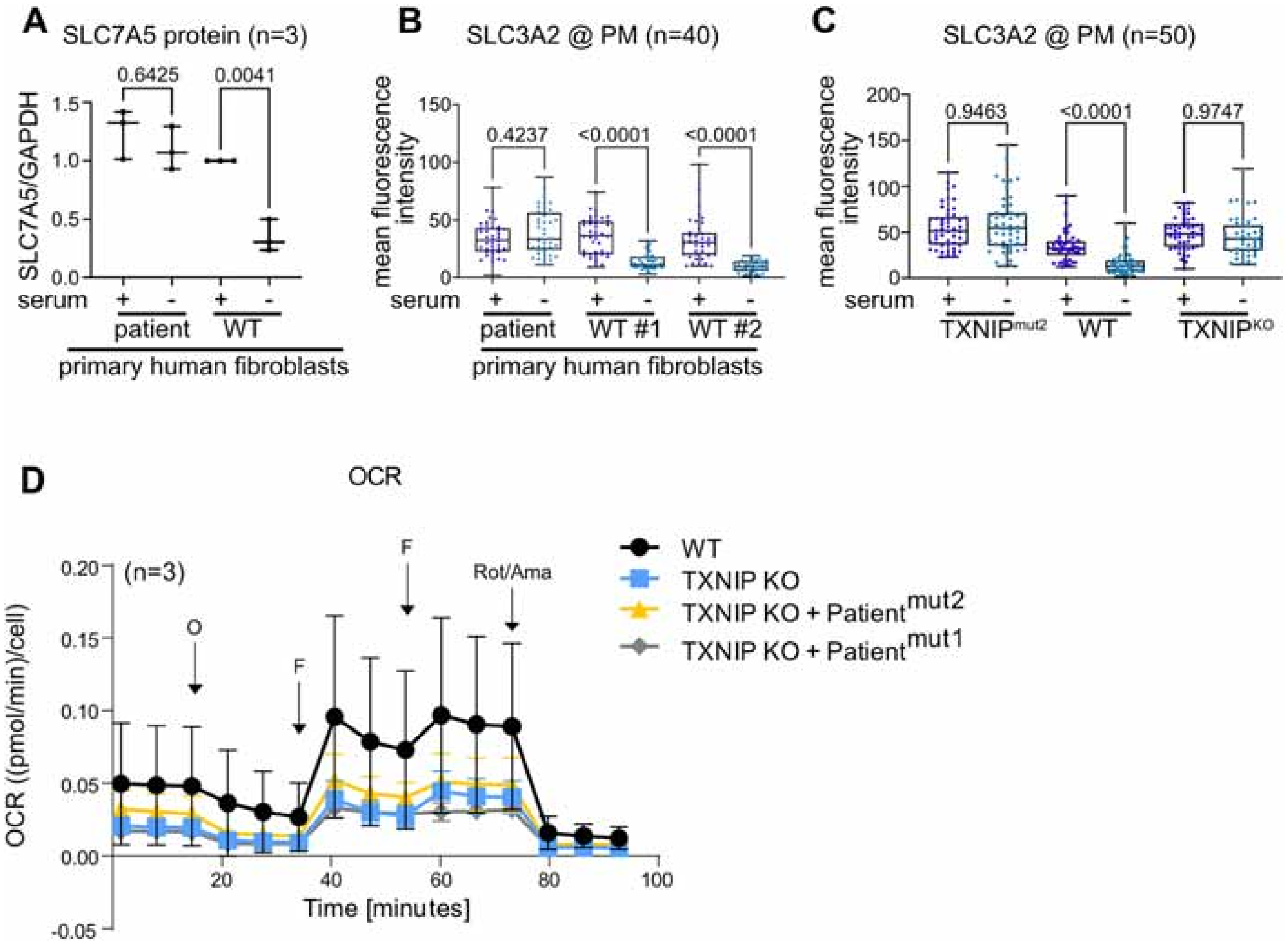

Figure EV3. The role of TXNIP in SLC7A5 endocytosis and mitochondrial function.

(**A**) WB quantification of SCL7A5 from primary human fibroblast normalized to GAPDH Box plots represent the median (centre line) and the interquartile range (25th to 75th percentile box). The whiskers show the minimum and maximum values ($n = 3$, one-way anova, Tukey's multiple comparison test). (**B**) Quantification of SLC3A2 IF signal at the PM of primary human fibroblast. Box plots represent the median (centre line) and the interquartile range (25th to 75th percentile box). The whiskers show the minimum and maximum values. ($n = 40$). (**C**) Quantification of SLC3A2 IF signal at the PM of the indicated RPE1 cells. Box plots represent the median (centre line) and the interquartile range (25th to 75th percentile box). The whiskers show the minimum and maximum values. ($n = 40$). (**D**) Mitochondrial oxygene consumption rate (OCR) of the indicated RPE1 cells using a Seahorse XF HS Mini Analyzer. The inhibitor injection time points are indicated by O (Oligomycin), F (FCCP), Rot/Ama (Rotenone/Antimycin A). Dots represent mean values ($n = 3$, SD).

© The Author(s)

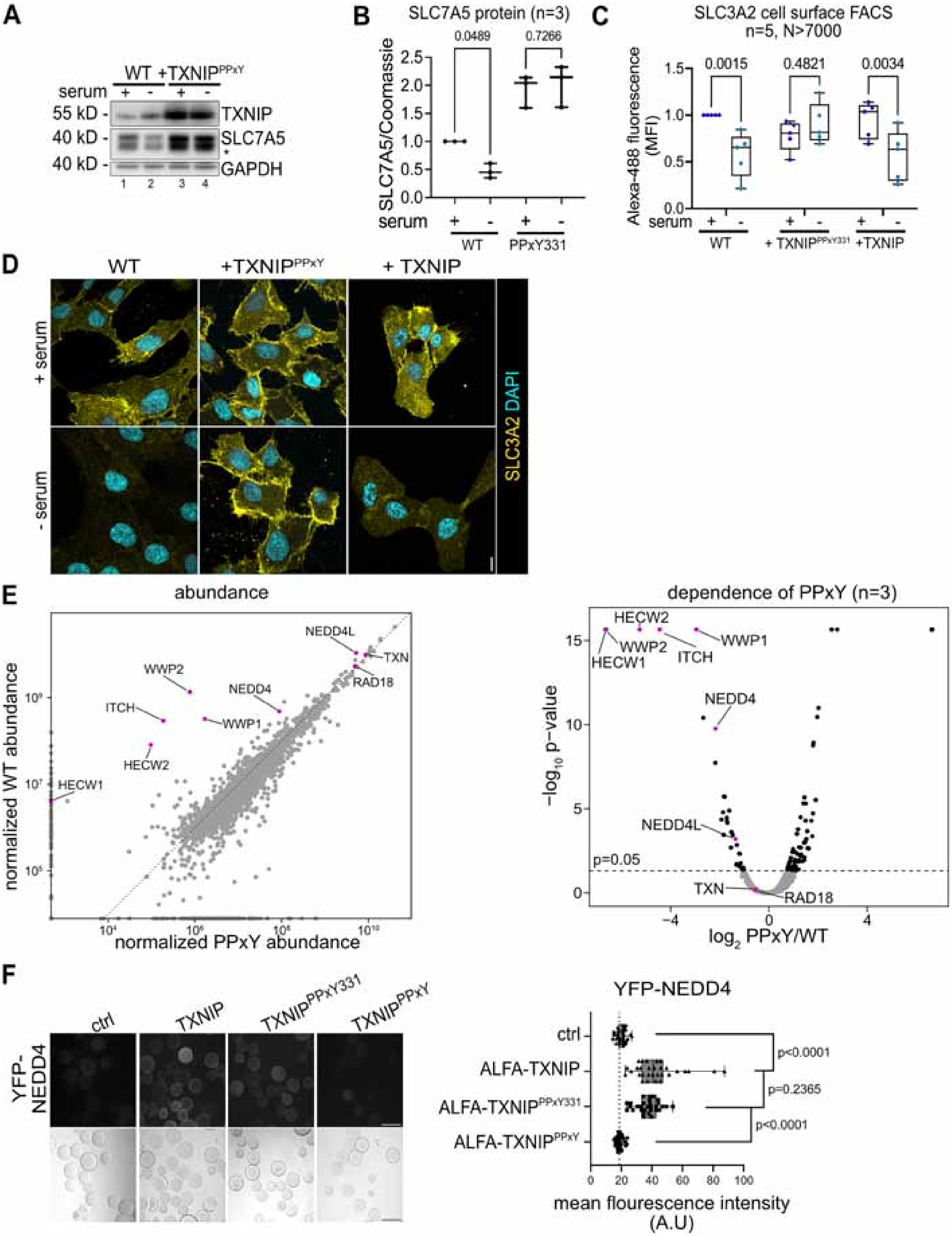
A
WT +TXNIP^PPxY
serum + - + -
55 kD - TXNIP
40 kD - SLC7A5
40 kD - GAPDH
1 2 3 4
B
SLC7A5 protein (n=3)
0.0489
0.7266
SLC7A5/Coomassie
serum
WT
PPxY331
C
SLC3A2 cell surface FACS
n=5, N>7000
0.0015
0.4821
0.0034
Alexa-488 fluorescence (MFI)
serum
WT
+ TXNIP^PPxY331
+TXNIP
D
WT
+TXNIP^PPxY
+ TXNIP
+ serum
- serum
SLC3A2 DAPI
E
abundance
normalized WT abundance
normalized PPxY abundance
NEDD4L
TXN
RAD18
WWP2
NEDD4
ITCH
WWP1
HECW2
HECW1
dependence of PPxY (n=3)
−log10 p−value
log2 PPxY/WT
p=0.05
F
ctrl
TXNIP
TXNIP^PPxY331
TXNIP^PPxY
YFP-NEDD4
ALFA-TXNIP
ALFA-TXNIP^PPxY331
ALFA-TXNIP^PPxY
p<0.0001
p=0.2365
p<0.0001
mean flourescence intensity (A.U)

Figure EV4. TXNIP interacts with HECT type ubiquitin ligases via its PPxY motif.

(**A**) Total cell lysates of RPE1 WT and TXNIP^{PPxY} cells that were grown in growth medium (+ serum) or were serum starved for 24 h (− serum) were analyzed by SDS-PAGE and WB with the indicated antibodies. (**B**) WB quantification of SLC7A5 normalized to the Coomassie-stained membrane as loading control Box plots represent the median (centre line) and the interquartile range (25th to 75th percentile box). The whiskers show the minimum and maximum values. ($n = 3$, one-way ANOVA, Tukey's multiple comparison test). (**C**) Quantification of SLC3A2 cell surface FACS of the indicated cells, normalized to proliferating WT cells. Box plots represent the median (centre line) and the interquartile range (25th to 75th percentile box). The whiskers show the minimum and maximum values. ($n = 5$, $N > 7000$ cells, two-way ANOVA, Sidak's multiple comparisons test). (**D**) IF of PFA fixed cells (WT, TXNIP^{KO} reconstituted with TXNIP^{PPxY} or TXNIP) under + serum or – serum conditions stained for SLC3A2 (yellow) and DAPI (cyan) were analyzed by confocal microscopy. The images show a single plane of a Z-stack. Scale bar = 10 µm. (**E**) Scatter plot comparing interactomes of ALFA-TXNIP with ALFA-TXNIPP^{PPxY} ($n = 3$). The HECT type ubiquitin ligases are highlighted in magenta. The abundances of all proteins were normalized to unspecific background binding to empty beads. Volcano plot of log2 transformed abundances. (**F**) Representative bright-field and fluorescence microscopy images of YFP-NEDD4 bound to ALFA-TXNIP (and the indicated PPxY mutants) immobilized to beads and image quantifications of the YFP-NEDD4 fluorescence signal. Box plots represent the median (centre line) and the interquartile range (25th to 75th percentile box). The whiskers show the minimum and maximum values. ($n = 40$ beads).

 © The Author(s)

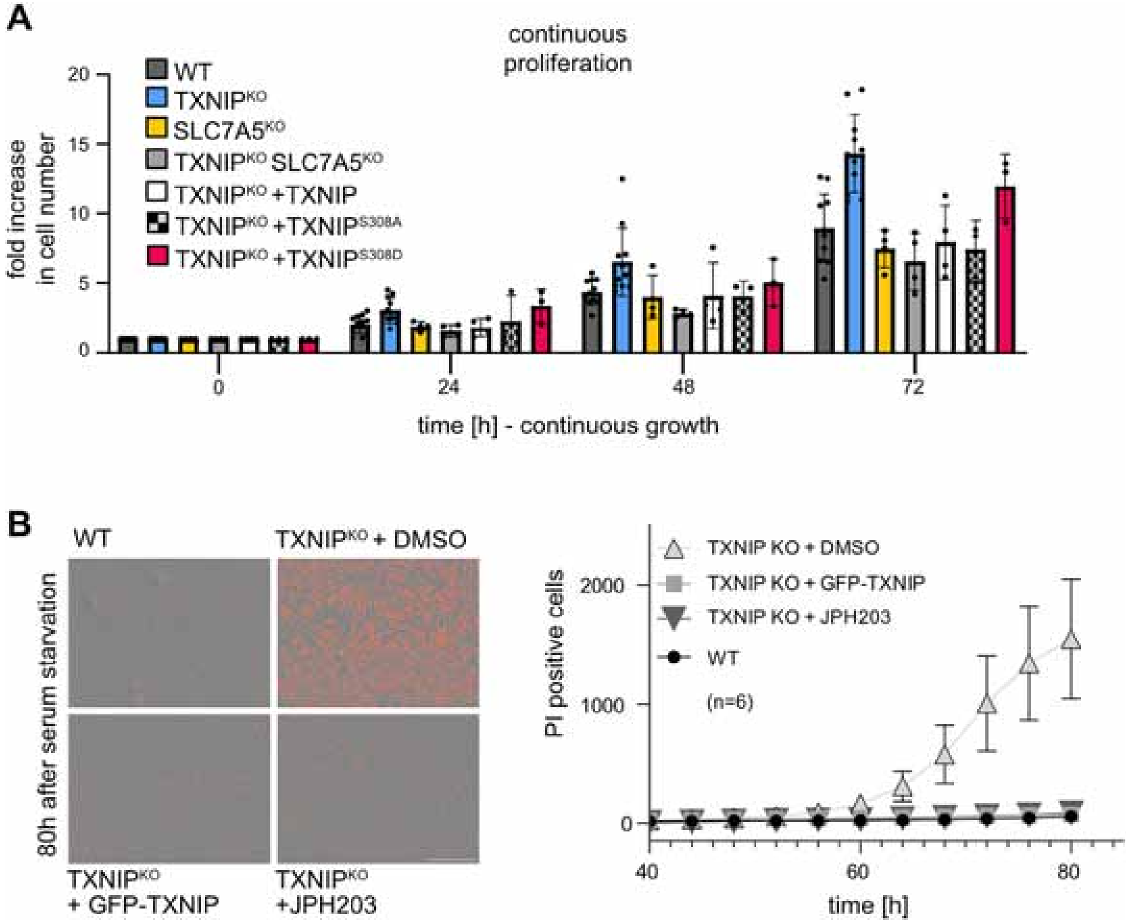

Figure EV5. TXNIP helps to control cell growth and survival during quiescence.

(**A**) The proliferation of the indicated cells was determined using CASY cell counting during the indicated time. The relative increase in cell number is normalized to 0 h and show as fold increase. Bar charts represent the mean value ($n > 3$, SD). (**B**) Analysis of cell death during quiescence of WT, TXNIP^{KO} TXNIP^{KO} (+ DMSO), TXNIP^{KO} reconstituted with GFP-TXNIP and TXNIP^{KO} treated with JPH203 (10 µM). 100.000 cells were seeded in growth medium (+ serum). After 24 h the medium was changed to starvation medium (-serum) containing propidium iodide (1 µg/ml). At the same time JPH203 (10 µM) was added. Cells and PI positive cells were continuously monitored for 80 h using live cell microscopy in an Incucyte incubator. Scale bar = 400 µm ($n = 6$, SEM).

© The Author(s)
